# Trillion-scale integrated framework for high-throughput materials databases and seamless sharing

**DOI:** 10.1038/s41467-026-78223-3

**Published:** 2026-10-07

**Authors:** Xiaoya Huang, Yu Liu, Shuo Shi, Yuanyuan Zhang, Zengzeng Liang, Miao Zhou, Hanwei Fu, Lei Zheng, Peng Kang

**Affiliations:** 1 Tianmushan Laboratory, Beihang University, Hangzhou, China; 2 School of Physics, Beihang University, Beijing, China; 3 State Key Laboratory of Artificial Intelligence for Materials Science, Beihang University, Beijing, China; 4 School of Materials Science and Engineering, Beihang University, Beijing, China; 5 Testing and Evaluation Center for High-Performance Fibers, Beihang University, Beijing, China; 6 Beihang Hangzhou Innovation Institute Yuhang, Hangzhou, China

**Keywords:** Computational methods, Metals and alloys, Software

## Abstract

Artificial intelligence is accelerating materials discovery, yet for complex systems such as high-entropy alloys, exhaustive exploration of their immense composition spaces is computationally intractable. Here we propose an integrated framework that enables large-scale exploration with reduced resource demands. Our framework uses stateless parallel computing with message queues and containerization, and a lakehouse architecture with columnar storage and metadata-based cloud sharing. Using this framework, we constructed a ten-trillion-scale database of six-principal-element high-entropy alloys in one week, totaling 17.5 terabytes and encompassing compositions, descriptors, and predicted properties. Compared with distributed frameworks, our approach cuts runtime-memory cost by 45%, storage by 90% versus row-based formats, and accelerates subset queries up to 180 times. A full database search completes in under eight minutes using less than four gigabytes of memory. Screening over 50 billion compositions with experimental validation across two alloy families confirms the practical utility of this framework for data-driven materials discovery.

## Abstract (web summary)

A new computing framework builds large-scale materials databases under resource constraints, with major gains in efficiency, storage, and query speed plus edge-device deployment. A ten-trillion-scale HEA database was built in one week.

## Introduction

Designing new materials has traditionally relied on empirical trial-and-error and mechanism-driven insight supported by theoretical and computational approaches, such as integrated computational materials engineering frameworks built for advanced alloy development^1,2^. In recent years, artificial intelligence (AI) has emerged as a data-driven methodology that offers a valuable complement to these traditional approaches^3–13^. By substituting physical experiments with rapid predictions, AI reduces the cost of iterative experimentation. It also mitigates the inherent limitations of mechanism-based paradigms, which frequently suffer from incomplete theoretical understanding or prohibitive computational costs when applied to complex systems. For instance, machine-learning interatomic potentials can accelerate atomistic simulations by orders of magnitude while maintaining near-first-principles accuracy^14,15^. The rapid evolution of large language models (LLMs), intelligent agents, and robotics further inspires great interest, illustrating a promising future^16–19^. Currently, significant achievements in AI for Materials Science have been realized across various classes of materials, such as metals^20–24^, ceramics^25–29^, and polymers^30–35^. These achievements encompass property optimization, novel materials design, and knowledge exploration. For example, the design of new SARS-CoV-2 nanobodies was facilitated through a virtual lab, which was supported by a team of LLM agents, with the entire workflow completed in just a few days^36^. Three key frontiers in AI for Materials Science are developing more comprehensive datasets, building more efficient and powerful AI models, and constructing autonomous generation-validation-optimization platforms. Progress along each frontier relies not only on advances in hardware and software, but also critically on the development of solutions or frameworks tailored to materials science. However, the significant knowledge barrier between computer science and materials science has led to inefficient progress in this regard, presenting a notable bottleneck for the advancement of AI for Materials Science.

Developing an efficient database framework is critical for the generation, management, and utilization of data in the AI-driven paradigm. This is particularly advantageous for complex material systems like high-entropy alloys (HEAs). HEAs are advanced multi-principal element alloys with excellent properties and exhibit a vast composition space that includes hundreds of billion potential compositions^37–39^. This vast composition space has encouraged significant interest in AI-driven research, with many achievements accomplished in phase prediction^40–42^, property optimization^43–46^, novel material design^47–49^, and knowledge exploration^50–52^. However, the vast composition space also renders the cost of theoretical calculations and AI predictions prohibitively high for exhaustive exploration under realistic resource constraints. Current studies have employed several alternative local optimization strategies, such as enlarging the step size of the component proportion or focusing on specific component families^42,44^. This may sacrifice the opportunity to discover extraordinary materials in unexplored compositional regions. In addition, the efficiency of materials design is always limited by small training datasets, which impairs the generalizability of AI models^53–55^. Bayesian methods are commonly used to optimize AI models through iteratively incorporating new experimental data^56–59^. For example, researchers proposed a Bayesian framework to discover novel copper alloys that break the trade-off relationship between electrical conductivity and hardness in four iterations^59^. However, the application of Bayesian approaches to complex material systems, such as HEAs, is challenging. It is because that as the iterations progress, the computational workload accumulates substantially. Consequently, it can be known that exploration in large-scale data space is a prohibitive computational challenge that demands a specialized technical framework to make it tractable.

The fulfillment of large-scale materials tasks needs to address key challenges in computing, data storage, and sharing. However, few existing solutions are specifically designed to tackle these challenges in an integrated manner. In terms of large-scale computations, the enormous computational and memory pressure far exceeds the processing capabilities of a single computer. Therefore, parallel computing across computational clusters is commonly used to accelerate the computation process. However, conventional parallel computing systems applied to materials science, such as the Simple Linux Utility for Resource Management (Slurm)^60^, suffer from massive input/output (I/O) overhead between computing nodes, significantly increasing computational costs. It can be effectively mitigated by modern distributed computing architectures like Ray^61^. Nevertheless, these architectures introduce a considerable technical barrier, as their effective applications require developers to manage low-level details, including state management and task orchestration. With regard to data storage, it is important to optimize storage costs and data querying efficiency. The comma-separated values (CSV) format is widely adopted for material data, but its application for large-scale datasets is limited by substantial storage overhead, slow transmission, and the inability to perform real-time queries^62^. In addition, relational databases based on row storage are generally utilized in computer fields, such as PostgreSQL and MySQL^63^, but exhibit low query efficiency for handling materials data with column characteristics. A database sharing chain is vital for scientific communication as it ensures that data adhere to the FAIR principles (findable, accessible, interoperable, and reusable). Furthermore, researchers demand not only basic data availability but also low-cost access and user-friendly mechanisms to integrate and repurpose data for custom applications, especially when dealing with large-scale datasets. Currently, the community lacks a cohesive architecture to address these problems. Therefore, it is necessary to develop an efficient framework tailored to the scale and structure of complex material systems, which can serve as an essential infrastructure to support comprehensive exploration across composition spaces.

In this work, we proposed an integrated framework for the efficient construction and management of large-scale material databases. Our framework focuses on addressing three key challenges: minimizing the complexity and overhead of parallel computing, optimizing the storage costs and the querying performance of the database, and improving the fluency of data sharing among researchers. The effectiveness of our framework is demonstrated through a trillion-scale database of HEAs, achieving a significant reduction in computational and storage costs while enabling rapid data retrieval. The case study also showcases how the framework serves as a powerful infrastructure for AI-driven research by accomplishing the design of high-performance HEAs.

## Results

### Framework overview and validation strategy

**Fig. 1:**
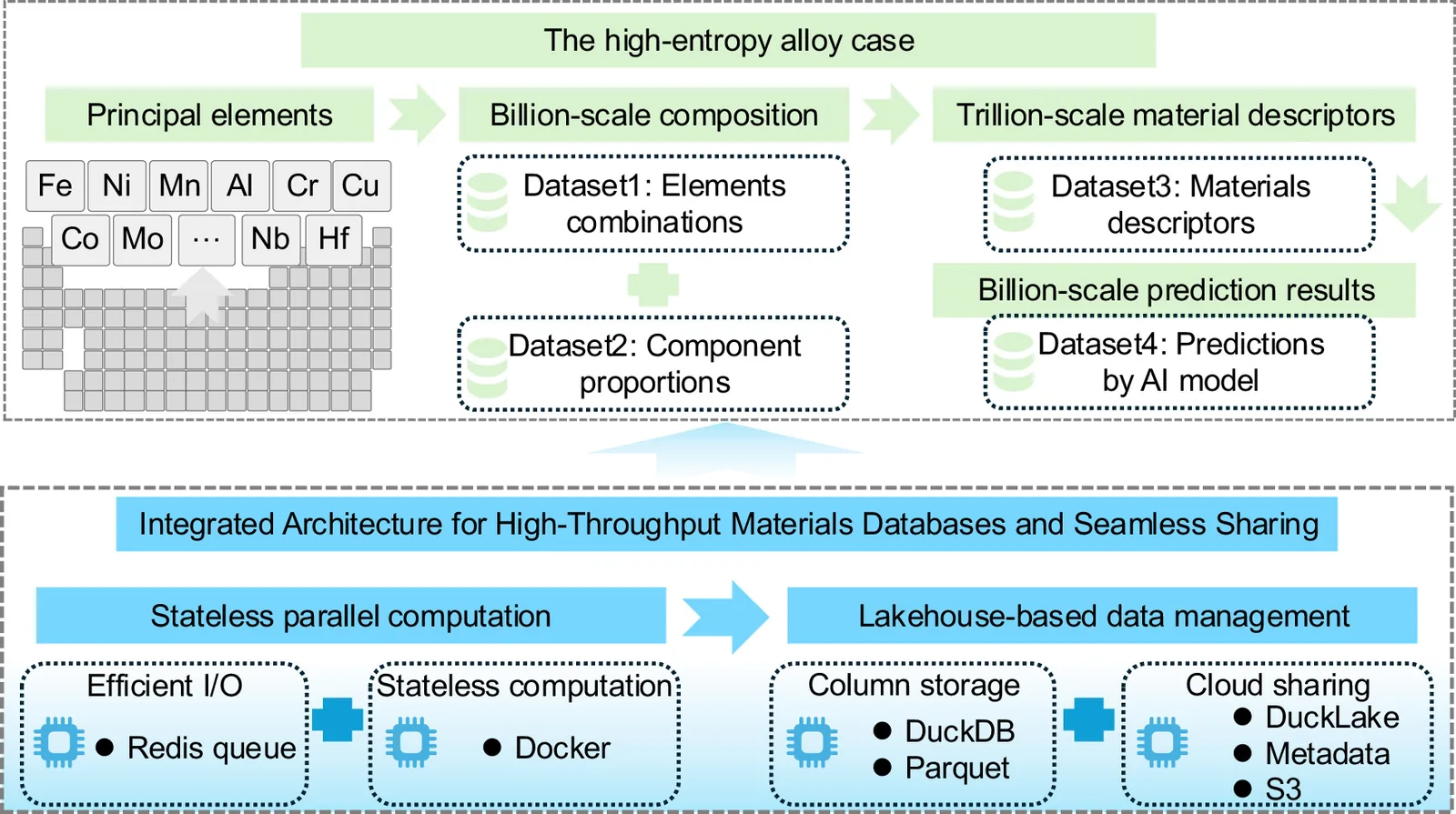
A schematic overview of this work. The upper part shows the high-entropy alloy case-study workflow from element selection to AI-predicted properties; the lower part shows the integrated framework comprising stateless parallel computation and lakehouse-based data management. I/O, input/output; S3, object storage compatible with the S3 protocol.

Figure 1 provides a schematic overview of our integrated technical framework for large-scale databases, using HEAs as a case study. The framework consists of two core modules, which are a stateless parallel computing architecture and a lakehouse-based data management architecture. Stateless computing enables each worker to process tasks independently, without retaining intermediate state between them. This design minimizes inter-node dependencies and I/O overhead, thereby facilitating elastic scaling and fault tolerance across distributed resources. Lakehouse architecture combines the low-cost scalable storage of data lakes with the structured querying capabilities of data warehouses^64^. It therefore supports efficient columnar storage, rapid analytical queries, and seamless cloud sharing of large scientific datasets. To address key challenges in computational efficiency, data management cost, and seamless data sharing, we integrate four categories of technologies, including efficient I/O, stateless computation, columnar storage, and cloud-native sharing. A series of HEAs with six principal elements is selected to validate the performance of our framework. The workflow of the HEA case study encompasses composition dataset generation, descriptor construction, and AI-based property prediction. Their vast composition space, which covers more than tens of billion potential compositions, offers an ideal platform for stress testing. The stateless parallel architecture is tested using workloads involving billions of compositions, while the lakehouse architecture is evaluated based on its ability to store, manage, and share the resulting large-scale data. The role of this framework in accelerating scientific discovery is demonstrated through a complete AI-driven workflow. This process spanned from composition space generation to high-throughput property prediction, culminating in the successful identification of novel HEA candidates with superior properties that are subsequently experimentally validated.

### Stateless parallel computing architecture

**Fig. 2:**
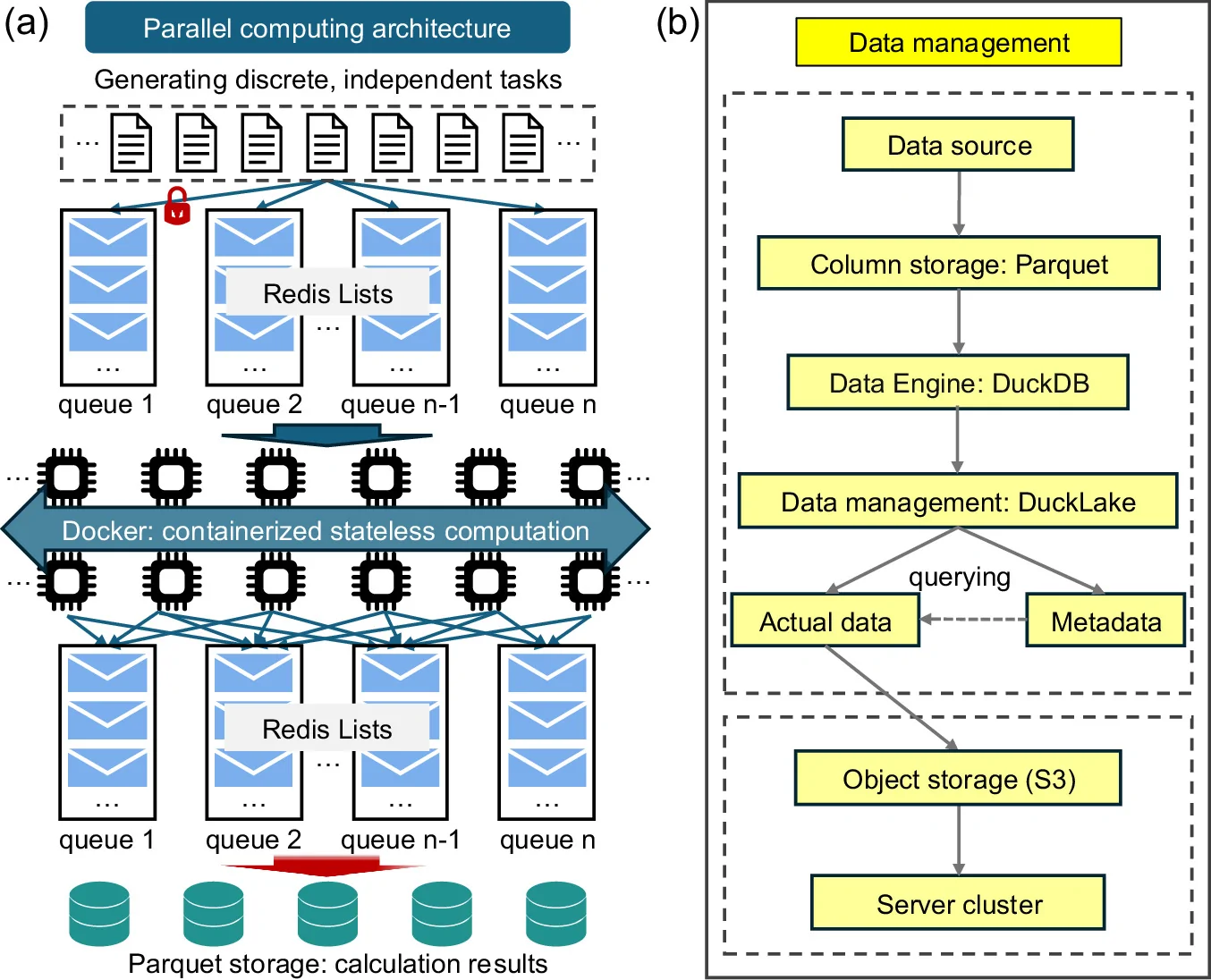
The integrated framework for large-scale databases. **a** Stateless parallel computing architecture utilizing message queues and containerization. Redis List message queues are deployed both upstream and downstream of the Docker containers for task generation and result collection. These containers are deployed across multiple compute nodes. **b** Lakehouse-based data management architecture. Raw data are stored in Parquet files. DuckDB is an embedded analytical engine. Object storage compatible with the S3 protocol is applied for cloud sharing. The metadata catalog can be downloaded to access the large-scale database.

This stateless parallel computing architecture is designed to efficiently execute large-scale materials computations across distributed computing resources. As shown in Fig. 2a, the architecture works by decoupling the core processes of task generation, task execution, and result collection. In this architecture, a large-scale computational task is first decomposed into independent units during the task generation process. These units are then distributed across available computing resources for parallel execution. Finally, the results are collected and aggregated in the result collection process. Redis List and Docker serve as the core enabling technologies. The task generation and result collection processes are implemented using Redis List. Redis List is a lightweight in-memory message queue that provides exceptionally fast read and write performance. It follows a producer-consumer pattern that decouples task generation from task processing^65^. Producers simply push jobs into the list, while consumers pull them from the other end. In our framework, Redis serves as the central task queue. Workers retrieve tasks from the queue independently, with no inter-worker communication or shared memory, making each worker stateless. Any available worker can perform any task, and the worker pool can be dynamically scaled without disrupting the workflow, ensuring optimal resource utilization across all computing nodes. In addition, to address congestion arising from faster write operations overwhelming the system, write locks are introduced to regulate task flow. These locks will be automatically released when message queues are depleted.

Computation execution occurs within isolated Docker containers that serve as stateless workers. Docker is an open-source containerization platform that encapsulates applications and their dependencies into portable containers, ensuring consistent execution across different environments^66^. This containerization guarantees computational reproducibility and environment consistency across distributed nodes. Following the computation process, the results are channeled into downstream message queues, where data are extracted and transformed into databases using dedicated management processes. This decoupled architecture offers a streamlined approach that straightforwardly resolves the inherent I/O overhead and state management complexities of large-scale parallel computing. A detailed description of the core operational workflow of the framework is provided in the Supplementary Information.

A standalone implementation of this framework is available at https://github.com/Wolido/idm-gridcore, which includes user guidance and a complete working example. To further lower the adoption barrier, an AI-agent skill that automates deployment and task execution via natural-language instructions is provided at https://github.com/Wolido/agent-idm-gridcore. Detailed descriptions of both repositories are also provided in the Supplementary Information.

### Lakehouse-based data management architecture

We proposed a data management chain based on a lakehouse architecture to enable efficient storage, utilization, and seamless sharing of scientific data between researchers (Fig. 2b). The workflow begins as computed results are ingested from downstream message queues. To optimize performance for subsequent analytical queries, the raw data is transformed and stored in Parquet, a columnar storage format that enables efficient selective column reads and achieves high compression ratios. These batch-generated Parquet files are then managed by a scalable, S3-compatible object storage system. It provides the foundation for durable, cost-effective cloud storage and seamless data sharing. To bridge the gap between low-level file storage and high-level data interaction, the architecture incorporates DuckDB as an analytical engine. DuckDB is an embeddable analytical database management system that executes SQL queries directly on Parquet files without requiring heavy-weight database servers^67^. The integration of these components forms a cohesive lakehouse architecture. Metadata can be automatically generated and used to manage large-scale data by offering a unified logical view of the entire database. It enables users to perform queries by downloading a small metadata catalog, eliminating the requirement for full database transfers. This architecture provides a solution to the challenges in managing large-scale databases and can also support the FAIR principle, ensuring that data are findable, accessible, interoperable, and reusable. A comprehensive glossary of the key technical terms used in this work, along with their definitions and roles in the framework, is provided in Supplementary Table 1.

### The large-scale database of HEAs

**Fig. 3:**
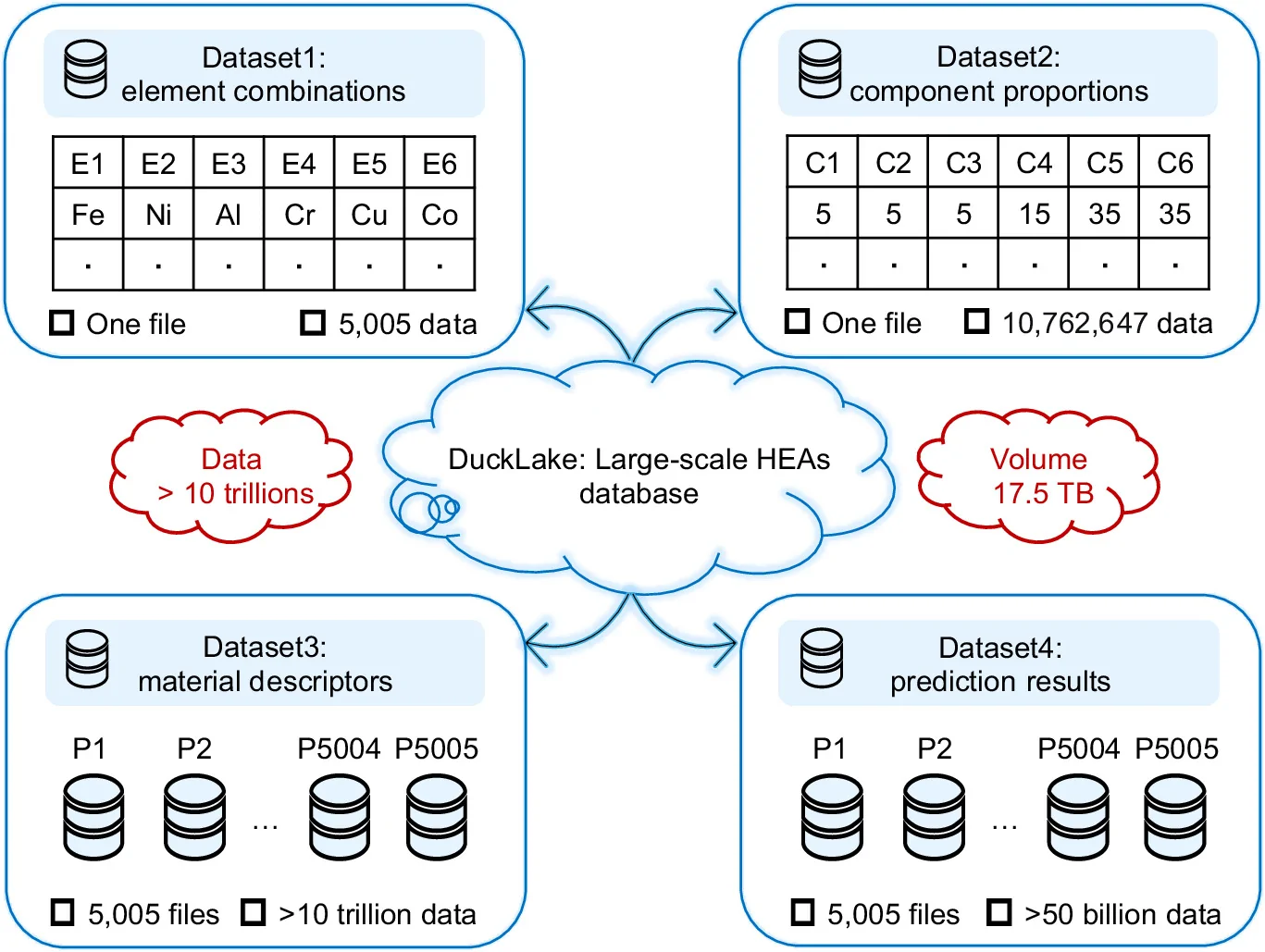
The schema diagram of the large-scale database of HEAs. It comprises four interconnected datasets managed by DuckLake. The element combinations dataset consists of 5005 six-element HEA families stored in one Parquet file (E: Element). Each HEA family covers 10,762,647 distinct component proportions stored in one Parquet file (C: Component proportion). The materials descriptor dataset includes more than 10 trillion data points and is further stored in 5005 Parquet files according to the HEA family (P: Parquet). The dataset 4 involves more than 500 billion compressive plasticity labels predicted by an AI model. They are also stored in 5005 Parquet files. The dataset has a volume of 17.5 TB.

A large-scale data lakehouse was established using the proposed integrated framework to manage six-principal element HEAs. Its schema architecture is illustrated in Fig. 3. The lakehouse integrates four specialized datasets containing elemental combinations, component proportions, material descriptors, and predicted properties. The first two datasets collectively define the vast compositional search space. We selected a pool of 15 elements commonly used in HEAs and enumerated all possible six-element combinations, yielding 5005 distinct HEA families. For each family, we systematically varied the component proportions to construct the composition space, with each family containing 10,762,647 individual compositions and over 50 billion HEAs in total. For each composition, we computed 194 material descriptors covering thermodynamic, electronic, atomic-size, mechanical, and crystallographic properties. Using our framework, these high-throughput calculations were completed in 1 week. These descriptors form a comprehensive and reusable feature library that maps the composition space to a set of physically meaningful feature vectors to facilitate AI-assisted materials design. The library supports multiple HEA design tasks, such as the phase and property predictions presented in Section “AI-driven Design of HEAs.” We then used a gradient boosting (GB) model to predict the room-temperature compressive plastic strain (*ε*_p_) for each alloy composition. This model can divide HEAs into three categories, low plasticity (*ε*_p_ < 10%), moderate plasticity (10% ≤ *ε*_p_ < 40%), and high plasticity (*ε*_p_ ≥ 40%). The predictions across the full composition space were accomplished in approximately two hours. This dataset enables the identification of promising HEAs candidates with excellent structural properties. The whole process produced a repository of more than 10 trillion data points with a total volume of 17.5 TB. The data is organized following an optimized storage scheme. The elemental combinations and the component proportions are stored as consolidated Parquet files, while material descriptors and plasticity predictions are partitioned into 5005 individual Parquet files organized by HEA families. This partitioned architecture significantly enhances query performance by reducing data scanning during retrieval operations. Additionally, it enables efficient caching mechanisms for frequently accessed parameters, such as material descriptors, substantially reducing computational overhead in iterative optimization workflows. The overall pictures of these datasets are provided in the Supplementary Information.

### Performance advantages of the framework

**Fig. 4:**
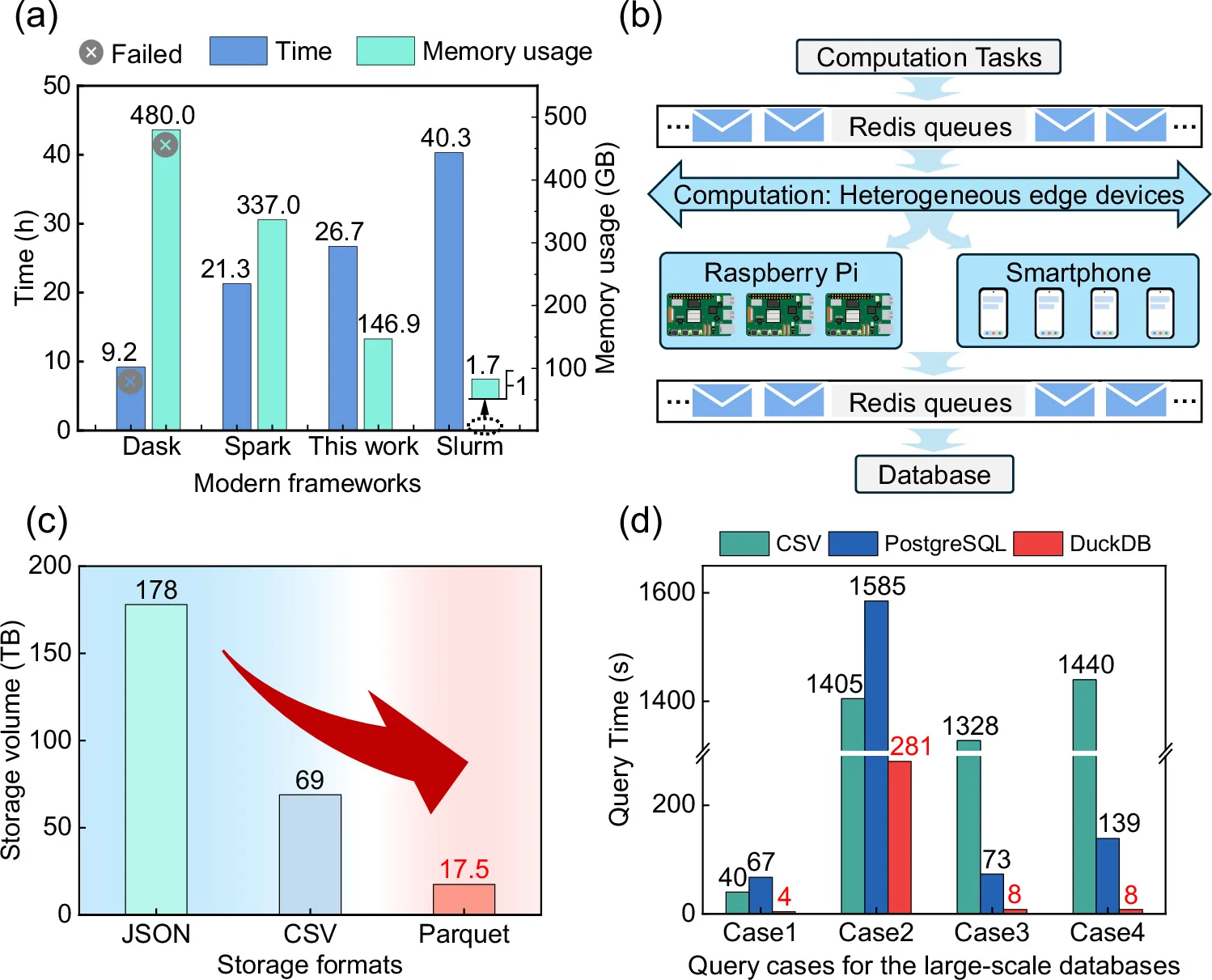
Performance benchmark of distributed frameworks and data management architectures for large-scale materials data. **a** Runtime and per-node peak memory usage for Spark, Dask, Slurm, and our framework on the HEA descriptor computation. **b** Schematic illustration of the framework architecture with heterogeneous edge devices as compute nodes. **c** Storage volume comparison between columnar Parquet and row-based formats (CSV and JSON). **d** Query speeds for four HEA cases using CSV, PostgreSQL, and DuckDB. The four query cases are defined in Table 1. Source data are provided as a Source Data file.

The proposed framework for the large-scale database has significant advantages in parallel computing acceleration and system scalability. To further quantify these advantages, we evaluated the performance of our framework against several representative distributed platforms, including Spark and Dask as execution-layer frameworks, and Slurm as a resource scheduler. Slurm in particular was selected as a baseline, given its widespread adoption in academic and research computing environments. We executed Spark, Dask, and our framework to compute materials descriptors for over 50 billion compositions across 5005 six-element HEA families. For Slurm, we restricted the test to a single family of 10,762,647 compositions to avoid excessive wear on storage media, as Slurm relies primarily on disk-based I/O rather than in-memory processing. As shown in Fig. 4a, Dask failed to accomplish the task. Memory usage on each worker grew at a quasi-linear rate of about 52.2 GB per hour, and the configured limit of 480 GB was reached after roughly 9.2 h, resulting in out-of-memory errors (Supplementary Fig. 6). Slurm finished a single HEA family of 10.7 million compositions in about 29 s, with a peak memory footprint of approximately 1.7 GB. Extrapolated to all 5005 families, the estimated runtime would be around 40.3 h. Spark completed the computation in approximately 21.3 h with a peak memory usage of 337.0 GB. For our framework, we evaluated two task configurations. The first configuration, in which each Docker worker pulled one composition at a time, was used for the initial database construction. Its memory consumption was only 30.1 MB per worker, totaling approximately 1.7 GB across a CPU node (56 cores). While this setup minimizes memory usage and is viable on resource-constrained edge devices, the Redis communication overhead becomes significant at the scale of billions of compositions. As a result, the full HEA database was completed within 1 week under this configuration. To increase throughput and enable a direct comparison with Spark, we adopted an alternative configuration in which each worker pulled 200,000 HEA compositions per batch. This approach reduced the Redis overhead and completed the computation in about 26.7 h, with a peak cluster-wide memory usage of 146.9 GB. These two configurations illustrate the tunable balance between memory footprint and computational speed that our framework offers.

The Spark result was optimized by shifting composition generation from the driver to the executors, such that the driver only handles element combinations instead of billions of individual compositions. This is consistent with Spark’s coarse-grained design and offers a fair performance baseline. By contrast, if the driver were to manage fine-grained tasks involving individual HEA compositions, the task count would climb to billions and push memory usage well beyond the 512 GB node limit, eventually causing job failure. Overall, our framework outperformed Slurm and Dask in efficiency and achieved a runtime comparable to that of Spark while using considerably less memory. To provide a quantitative comparison of overall resource efficiency, we used the product of runtime and peak memory usage as a combined metric. Dask failed to complete the computation due to memory exhaustion, and Slurm was excluded from this metric because its performance is dominated by disk I/O rather than memory usage. The time-memory product for Spark was approximately 7.18 × 10^3^ GB h, while our framework achieved approximately 3.97 × 10^3^ GB h, representing a 45% reduction. As shown in Fig. 4b, we tested two edge devices as compute nodes, a Raspberry Pi and a smartphone. Both devices successfully connected to the same Redis queue, pulled subtask data, and completed the descriptor calculations. For most edge devices, we recommend running computations inside Docker containers to ensure fast deployment and environment consistency. For devices that cannot run Docker, such as the smartphone used in this test, the computation code can be executed directly. Our framework can also be integrated into existing workflow orchestration systems, such as Nextflow, though it remains fully functional and deployable independently without such orchestration. These results confirm that the framework can effectively utilize a wide range of computing resources, from dedicated cluster nodes to edge devices.

The proposed framework also exhibits excellent performance in reducing storage volume, rapid data retrieval, and seamless data sharing. Materials data exhibit several distinct characteristics in practical applications. They are typically utilized in the form of column vectors for tasks, such as distribution analysis and global optimization. Additionally, queries often focus on specific columns without requiring a full database. These data also exhibit a read-intensive and write-rarely access pattern. Accordingly, the Parquet format is particularly suited for such applications, enabling both high storage efficiency and rapid data retrieval. Parquet also exhibits excellent storage savings due to its compression algorithm based on the homogenized column data. To demonstrate the storage efficiency of our framework, we also saved all calculated data for the six-component HEAs (Datasets 1–4) into two row-oriented formats commonly used in materials data management, CSV and JSON. As shown in Fig. 4c, Parquet required the least storage space at 17.5 TB, achieving up to 90% storage reduction compared with CSV (69 TB) and JSON (178 TB). JSON incurs additional overhead because it stores key names and structural delimiters for every record.

Parquet files are difficult to modify, which limits the append and update of data. In this work, DuckLake was introduced to address these limitations. It uses a metadata layer to intelligently manage these Parquet files. The metadata catalog can be regarded as a super directory that records file composition, table structure, partitions, and operation history. Users can remotely access our entire database by downloading the tens of megabytes of metadata catalog. To evaluate the performance of our framework on large-scale database queries, we compared CSV and PostgreSQL against our approach on both full-database and subset queries using the 17.5 TB HEA database. CSV represents the typical file-based workflow, and PostgreSQL serves as a representative row-oriented relational database. Before executing the queries, we converted the dataset into the corresponding formats required by CSV and PostgreSQL. We executed six representative query cases, as detailed in the Table 1 footnote. Among them, the four subset queries are shown in Fig. 4d, while the remaining two full-database queries are summarized in Table 1. It can be seen that our proposed framework shows a significantly faster response compared to the other two approaches, with measured speedups increasing up to 180 times. Specifically, remotely retrieving the 10,762,647 HEAs compositions through DuckLake only takes 4 s. Querying an approximately 4 GB Parquet file of the material descriptors for a HEA family takes 4 min 41 s. Checking specific columns in this Parquet format is significantly faster; for example, accessing 4 columns takes only 8 s. The reverse query of alloy compositions in a HEA family according to the values of material descriptors was completed in around 8 s. These queries can even be completed immediately on private networks. For the full-database queries, maintaining a persistent DuckDB connection completed the query in 3 min and 22 s and required approximately 27 GB of memory. In comparison, releasing and re-establishing the connection after every 100 Parquet files reduced the memory footprint to under 4 GB, at the cost of increasing the execution time to 7 min and 38 s. These results demonstrate that our database framework enables efficient and rapid analysis of trillion-scale databases on a single machine.

**Table 1:** The results of querying cases of the large-scale database

Cases	File volumes^a^	Test network	CSV	PostgreSQL	DuckLake
Case1^b^	150.2 MB	Public	40 s	67 s	4 s
Case2^c^	4.1 GB	Public	23 min 25 s	26 min 25 s	4 min 41 s
Case3^d^	3.3 GB	Public	22 min 8 s	1 min 13 s	8 s
Case4^e^	3.8 GB	Public	24 min	2 min 19 s	8 s
Case5^f^	17.5 TB	Private	–	–	3 min 22 s
Case6^g^	17.5 TB	Private	–	–	7 min 38 s

^a^It represents the underlying storage volumes that data queries must access.

^b^Case1 is querying the entire parquet file of compositions.

^c^Case2 is querying 4 columns in a materials descriptors file.

^d^Case3 is querying an entire parquet file of 194 materials descriptors of a HEA family.

^e^Case4 is the reverse query of alloy compositions in a HEA family according to the values of material descriptors.

^f^Case5 is the reverse query of alloy compositions in 5005 HEA families according to the values of material descriptors by maintaining a persistent DuckDB connection.

^g^Case6 is the reverse query of alloy compositions in 5005 HEA families according to the values of material descriptors by releasing and re-establishing the connection after every 100 Parquet files.

### AI-driven design of HEAs

**Fig. 5:**
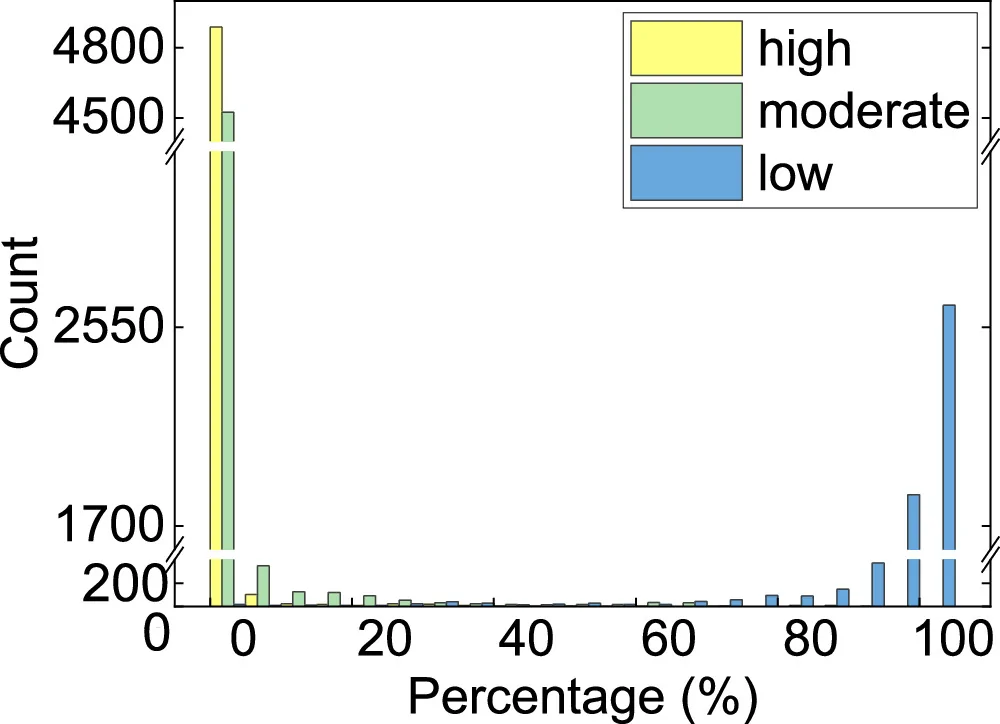
Statistical distribution of the predicted plasticity labels across 5005 HEA families. The horizontal ordinate is the percentage of HEAs showing three plasticity labels (high, moderate, and low) in a HEA family. High: high-plasticity (*ε*_p_ ≥ 40%), moderate: moderate-plasticity (10% ≤ *ε*_p_ < 40%), and low: low-plasticity (*ε*_p_ < 10%). Source data are provided as a Source Data file.

Based on the high-throughput predictions, we performed statistical analysis of the predicted plasticity categories across the 5005 HEA families. The percentage of each category within each family was counted and shown in Fig. 5. It can be seen that most HEAs families show low percentages of high and moderate plasticity samples. Approximately 50% of the HEA families were characterized by exclusively low-plasticity samples, whereas no family exhibited entirely high or moderate plastic deformation capacity. The remaining around 50% of HEA families contained mixtures of sample categories, indicating an obvious trend of performance changes with the variation of components. The top five HEA families with the highest proportions of high plasticity samples are Al-Ti-Nb-Ta-V-Hf, Al-Ti-Nb-Ta-V-Zr, Ti-Nb-Ta-V-Zr-Hf, Al-Nb-Ta-V-Zr-Hf, and Al-Ti-Nb-Ta-W-Hf, with respective percentages of 71, 70, 64, 59, and 52%.

The GB model was established for predicting the plasticity of HEAs showing single body center cubic (BCC) solid solution phase. Therefore, we also applied two commonly used empirical criteria to select BCC HEAs. Firstly, the two-parameter criterion based on atomic size mismatch (δ*r*) and thermodynamic parameters (*Ω*) was applied to classify the solid solution phase (S). It can recognize the common phase exhibiting in HEAs, solid solution phase (S), intermetallic compounds (I), bulk metallic glasses (BMG, showing amorphous phase), and the mixture of solid solution phase and intermetallic compounds (S+I). HEAs that satisfy *Ω* ≥ 1.1 and δ*r* ≤ 6.6% exhibit a high probability of S phase^68^. In addition, researchers proposed a criterion of valence electron concentration (VEC) to identify the type of solid solution phase. According to this criterion, HEAs with VEC ≥  8.0 tend to form FCC phases, those with VEC < 6.87 preferentially develop BCC structures, while systems with 6.87 ≤ VEC < 8.0 typically exhibit mixed BCC and FCC phase formations^69^. Therefore, we queried the range of three parameters *Ω*, δ*r*, and VEC of these five HEAs families as exhibited in Table 2. It can be seen that TiNbTaVZrHf HEAs are mostly possible to exhibit a single BCC solid solution phase, with 0.000 < *Ω*^−1^ < 0.094, 0.032 < δ*r* < 0.061 and 4.15 < VEC < 4.85. Accordingly, experimental validation was performed on the Ti-Nb-Ta-V-Zr-Hf HEA family. In addition, we also examined the Mo-Ti-Nb-Ta-W-Hf HEA family to further evaluate the generalizability of the AI predictions. The Mo-Nb-Ta-W quaternary family is a prototypical refractory high-entropy alloy (RHEA) family showing excellent high-temperature strength. For example, the equiatomic Nb_25_Mo_25_Ta_25_W_25_ HEA exhibits a yield strength above 400 MPa at 1600 °C^70^. However, these HEAs typically exhibit severe room-temperature brittleness. High-throughput screening reveals that the addition of Ti and Hf to the Mo-Nb-Ta-W family can enhance its compressive plasticity to above 40%. According to our database, the predicted fraction of high-plasticity HEAs in this family is approximately 0.09%. Table 2 shows that the calculated parameters for the Mo-Ti-Nb-Ta-W-Hf family meet the phase criteria, confirming that this family also forms a single BCC solid solution. As listed in Table 3, we selected nine compositions that were predicted as “high” plastic label and BCC phase for experimental validation, including five Ti-Nb-Ta-V-Zr-Hf HEAs and four Mo-Ti-Nb-Ta-W-Hf HEAs.

**Table 2:** The range of phase parameters of HEA families

HEAs	*Ω*^−1^^a^	δ*r*^b^	VEC^c^
Al-Ti-Nb-Ta-V-Hf	0.075–1.236	0.064–0.157	3.80–4.80
Al-Ti-Nb-Ta-V-Zr	0.087–1.447	0.064–0.157	3.80–4.80
Ti-Nb-Ta-V-Zr-Hf	0.000–0.094	0.032–0.061	4.15–4.85
Al-Nb-Ta-V-Zr-Hf	0.072–1.459	0.070–0.161	3.80–4.80
Al-Ti-Nb-Ta-W-Hf	0.099–1.170	0.061–0.158	3.85–5.15
Mo-Nb-Ta-W-Ti-Hf	0.000–0.171	0.024–0.055	4.40–5.60

^a^*Ω*^−1^ is a descriptor for predicting the phase stability of HEAs, as shown in Eq. 6.

^b^δ*r* is a atomic size mismatch that can be calculated by Eq. 2.

^c^VEC is the average value of valence electron concentration that can be calculated by Eq. 14.

**Table 3:** The predicted and experimental *ε*_p_ of the selected HEAs

Sample ID^a^	Composition (at. %)	Prediction^b^	Experimental *ε*_p_ (%)
TNTVZH1	Ti_14_Nb_18_Ta_8_V_13_Zr_35_Hf_12_	High	>40
TNTVZH2	Ti_18_Nb_18_Ta_26_V_6_Zr_13_Hf_19_	High	>40
TNTVZH3	Ti_25_Nb_18_Ta_8_V_24_Zr_19_Hf_6_	High	>40
TNTVZH4	Ti_30_Nb_18_Ta_5_V_6_Zr_12_Hf_29_	High	>40
TNTVZH5	Ti_34_Nb_30_Ta_5_V_5_Zr_21_Hf_5_	High	>40
MTNTWH1	Mo_5_Ti_25_Nb_18_Ta_16_W_6_Hf_30_	High	36.5 ± 0.5
MTNTWH2	Mo_5_Ti_35_Nb_30_Ta_9_W_7_Hf_14_	High	>40
MTNTWH3	Mo_5_Ti_35_Nb_31_Ta_14_W_5_Hf_10_	High	>40
MTNTWH4	Mo_7_Ti_12_Nb_11_Ta_30_W_5_Hf_35_	High	23.5 ± 0.5

^a^TNTVZH and MTNTWH denote two distinct HEA families, Ti-Nb-Ta-V-Zr-Hf and Mo-Ti-Nb-Ta-W-Hf, respectively.

^b^Predicted plasticity regime based on the GB classifier: high (*ε*_p_ ≥ 40%).

**Fig. 6:**
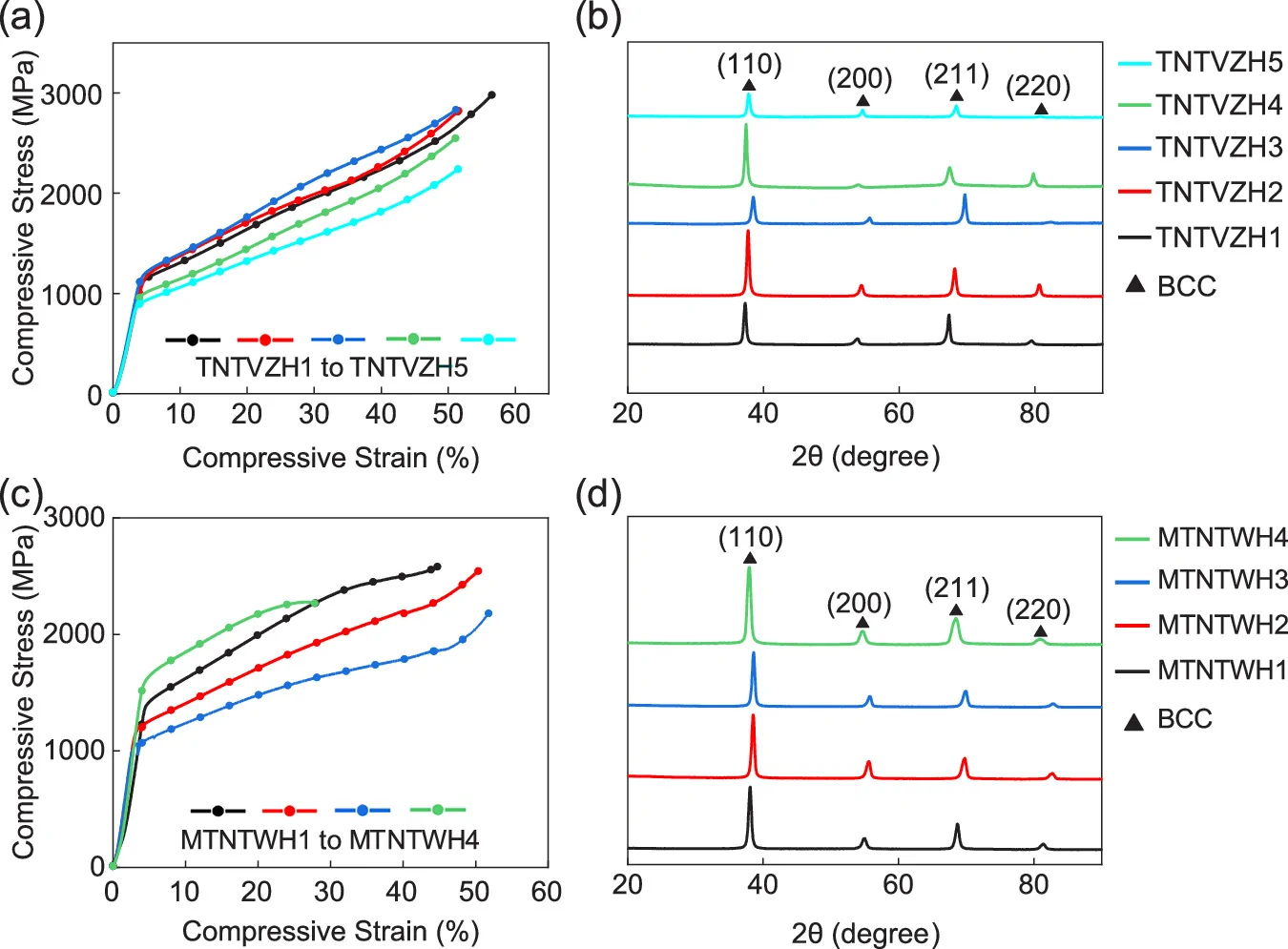
Experimental validation of the selected HEAs. **a**, **b** Compressive stress-strain curves and XRD patterns for the Ti-Nb-Ta-V-Zr-Hf HEAs (labeled as TNTVZH HEAs). **c**, **d** Compressive stress-strain curves and XRD patterns for the Mo-Ti-Nb-Ta-W-Hf HEAs (labeled as MTNTWH HEAs). The replicate test results for compressive stress-strain curves are provided in Supplementary Figs. 7 and 8. Source data are provided as a Source Data file.

The compressive stress-strain curves (Fig. 6a) reveal that all five Ti-Nb-Ta-V-Zr-Hf HEAs exhibit compressive *ε*_p_ exceeding 40%, in full agreement with their predicted high-plasticity labels. For the Mo-Ti-Nb-Ta-W-Hf family, two of the four selected compositions exhibit compressive *ε*_p_ above 40%, while the other two show moderate values of 36.5% and 23.5%, respectively (Fig. 6c). X-ray diffraction (XRD) patterns (Fig. 6b,d) confirm the single-BCC phase for all nine compositions. Energy-dispersive X-ray spectroscopy (EDS) mapping further confirmed that the measured compositions are consistent with the nominal values (as shown in Supplementary Table 3). The successful validation across two distinct HEA families demonstrates the generalizability of our AI predictions. The AI-driven screening over 50 billion compositions was completed in less than 2 h, and the subsequent statistical analysis and data querying took only a few minutes. This high efficiency establishes a solid foundation for large-scale exploration and design of HEAs, enabling systematic investigation of previously less-accessible composition spaces.

## Discussion

In this work, we proposed an integrated framework for the computational exploration of enormous compositional spaces in complex material systems. By combining stateless parallel computation with a lakehouse-based data management architecture, our framework demonstrates substantial improvements in computational efficiency, storage compression, and data retrieval speed compared with conventional distributed frameworks and row-based formats. We have validated its practical utility through the construction of a trillion-scale HEA database and the experimental discovery of multiple novel HEAs with high compressive plasticity at room temperature. The framework also operates effectively on heterogeneous edge devices, confirming its adaptability to resource-constrained environments beyond conventional computing clusters.

The parallel performance of our framework derives from the following architectural mechanisms. Spark requires a dedicated runtime environment that consumes baseline memory for scheduling, serialization, and shuffle services regardless of task granularity. Dask workers are long-running processes that retain memory pages even after garbage collection, causing memory to accumulate over time. Slurm, while memory-efficient, relies primarily on disk-based I/O and writes task outputs directly to disk, generating fragmented files that complicate data aggregation across multiple nodes. In our single-family test, tasks were evenly distributed across 560 cores, producing 560 output files. Extrapolating to the full set of 5005 HEA families would produce roughly 2.8 million files, placing intense pressure on the metadata cache and exhausting the filesystem caches of the storage system. The post-processing stage would then require aggregating this massive number of files, which would consume substantial CPU and I/O bandwidth and ultimately become a critical bottleneck for overall throughput. In this work, we used Redis queues for task distribution and result collection, with task executions completed in Docker containers. The upstream and downstream nodes of the containers are both Redis queues, providing an isolated and stateless execution environment. Workers consume memory only during active task execution (Supplementary Fig. 6), and task distribution through Redis eliminates the need for high-speed internal network interconnects among worker nodes. The stateless design of our framework inherently supports elastic scaling of compute nodes and cross-cluster distributed computing, allowing resources to be aggregated across physical boundaries without I/O overhead or memory accumulation. The two test configurations demonstrate the flexibility of our framework in balancing memory usage and computational time. Therefore, it also accommodates heterogeneous and resource-constrained environments, including edge devices. This edge-computing capability also lays the groundwork for integrating the framework into self-driving laboratory environments for real-time experimental feedback.

The scalability of our framework across HEA systems with varying numbers of principal components is also an important consideration for broader applications. The total number of compositions reaches approximately 1.6 × 10^7^ for four-component systems, 1.7 × 10^9^ for five-component systems, 5.4 × 10^10^ for six-component systems, 7.5 × 10^11^ for seven-component systems, and 5.2 × 10^12^ for eight-component systems, all drawn from a pool of 15 elements. Four- and five-component systems are computationally and storage-wise straightforward, as their composition spaces remain manageable. In contrast, seven- and eight-component systems bring substantial increases in both computational cost and storage footprint, with these resource demands roughly tens to hundreds of times higher than those of the six-component system. The computational time, however, can be flexibly managed by scaling the number of computing nodes or by adjusting the configurations of our framework, such as the number of Redis queues and the batch size per worker. Storage, by contrast, is likely to become the primary bottleneck for higher-order HEA systems. Even with the substantial storage compression achieved by our columnar format, the eight-component space still demands considerable storage capacity, which can be accommodated through scalable storage configurations or mitigated by adopting adaptive alloy search strategies that narrow the search space.

The excellent performance of our framework in high-throughput computation and data management offers benefits for large-scale materials design, enabling research avenues that were previously inaccessible due to resource constraints. The framework is designed for computational tasks that can be partitioned into independent and repetitive units. For tasks involving interdependent subtasks or heterogeneous workloads, effective application requires careful design of subtask partitioning strategies. Beyond one-shot screening, it can also provide a robust foundation for iterative workflows, such as active learning. For small-scale tasks where all data can be kept in memory, stateful execution can achieve better efficiency by caching intermediate results on compute nodes. As the task scale grows to billions of compositions, however, this approach becomes impractical. The accumulation of intermediate states in memory across iterations leads to unsustainable memory growth and eventual exhaustion. Our framework overcomes this barrier by decoupling computation from state management. The stateless computation layer, paired with a storage layer that persistently maintains intermediate results across rounds, enables iterative execution without the memory accumulation or recovery overhead typical of stateful designs. This separation allows the computational efficiency of our stateless framework to be fully leveraged in multi-round exploration.

The present demonstration focuses on BCC HEAs, but our framework is not restricted to this context. Extension to more complex systems is feasible with appropriately designed materials strategies. These systems include those exhibiting precipitation, segregation, or magnetism. For precipitation-strengthened alloys, phase-specific prediction models could be constructed using phase data. For segregation-prone systems, dedicated datasets could be built using segregation parameters as prediction targets. For magnetic systems, spin-polarized first-principles calculations would be required to capture the underlying physics. More complex systems may require more accurate descriptors than the empirical formulas used in the present work. The current descriptors are relatively simplified, yet they are widely adopted as an effective and computationally efficient approach in the field. Future work will therefore leverage our computational framework to incorporate multi-scale descriptors, such as melting temperature and shear modulus from first-principles calculations. This will require the development of tailored subtask partitioning strategies.

## Methods

### Data generation for the HEA case study

We selected 15 elements commonly used in HEAs (Fe, Ni, Mn, Al, Cr, Cu, Co, Mo, Ti, Nb, Ta, W, V, Zr, and Hf) and enumerated all possible six-element combinations, each representing a distinct HEA family. For each family, we exhaustively listed the component proportions to construct the composition space, with the proportion of each principal element ranging from 5 to 35 at.% in 1 at.% intervals. This range and step size were chosen to reflect typical experimental tolerances in alloy fabrication and to ensure comprehensive coverage of the composition space. This approach allows all HEA families to share a unified component proportions dataset, avoiding redundant computations. Furthermore, 194 material descriptors are calculated based on the compositions^42^. A detailed description is provided as follows:

1. Ten material descriptors, which are two atomic size mismatch values (*Λ* and *γ*), mixing entropy (Δ*S*_mix_), mixing enthalpy (Δ*H*_mix_) and its mismatch value (δΔ*H*_mix_), vacancy formation energy (*E*_v_), a descriptor for predicting the phase stability of HEAs (*Ω*^−1^), two parameters related to plastic deformation behaviors in HEAs (*G*/*G*_0_ and *K*/*G*), and a temperature parameter (*T*_b_ − *T*_m_), were obtained using specific formulas. The formulas are shown in Eqs. 

1–7:

1 $${\rm\Lambda }={\rm\Delta }{S}_{{\mathrm{mix}}}/{{\delta}} {{\rm{r}}}^{2}$$

2 $$\gamma=\left(1-\sqrt{\dfrac{{({r}_{{{\rm{s}}}}+\bar{r})}^{2}-{\bar{r}}^{2}}{{({r}_{{{\rm{s}}}}+\bar{r})}^{2}}}\right)/\left(1-\sqrt{\dfrac{{({r}_{{{\rm{l}}}}+\bar{r})}^{2}-{\bar{r}}^{2}}{{({r}_{{{\rm{l}}}}+\bar{r})}^{2}}}\right)$$

where δ*r* is also an atomic size mismatch value of HEAs and can be calculated by δ*r* =  $\sqrt{{\sum }_{i=1}^{n}{c}_{{{\rm{i}}}}{(1-{r}_{{{\rm{i}}}}/\overline{r})}^{2}}$. *r*_i_ is the atomic size of the i-th element and $\overline{r}$ is the average value of these elements with $\overline{r}={\sum }_{i=1}^{n}{c}_{{{\rm{i}}}}{r}_{{{\rm{i}}}}$. *γ* is a kind of topology parameter that is proposed as a more precise descriptor of the atomic size mismatch. *r*_s_ is the smallest atomic size among the principal elements, and *r*_l_ is the largest atomic size. *Λ* and *γ* are also always used to analyze the phase and strength of HEAs.

3 $$\Delta {S}_{{{\rm{mix}}}}=-{{\rm{R}}}\mathop{\sum }_{i=1}^{n}{c}_{{{\rm{i}}}}{{\rm{nl}}}{c}_{{{\rm{i}}}}$$

4 $$\Delta {H}_{{{\rm{mix}}}}=4{\sum }_{i=1,i\ne j}^{n}{c}_{{{\rm{i}}}}{c}_{{{\rm{j}}}}{H}_{{{\rm{ij}}}}^{{{\rm{mix}}}}$$

5 $${{\delta \Delta }}{H}_{{\rm{mix}}}=\sqrt{{\sum }_{i=1,i\ne j}^{n}{c}_{{{\rm{i}}}}{c}_{{{\rm{j}}}}{\left(1-\frac{{H}_{{\rm{ij}}}^{{\rm{mix}}}}{{\Delta }{H}_{{\rm{mix}}}}\right)}^{2}}$$

6 $${\Omega }^{-1}=\dfrac{1000\left\vert \Delta {H}_{{{\rm{mix}}}}\right\vert }{{T}_{{{\rm{m}}}}\Delta {S}_{{{\rm{mix}}}}}$$

7 $${E}_{{{\rm{v}}}}=4{\sum }_{i=1,i\ne j}^{n}{c}_{{{\rm{i}}}}{c}_{{{\rm{j}}}}{E}_{{{\rm{ij}}}}^{{{\rm{v}}}}$$

where *c*_i_ and *c*_j_ denote the mole fractions of the *i*-th and *j*-th elements, respectively. *R* = 8.314 J mol^−1^ K^−1^ is the universal gas constant. ${H}_{{{\rm{ij}}}}^{{{\rm{mix}}}}$ and ${E}_{{{\rm{ij}}}}^{{{\rm{v}}}}$ are the binary mixing enthalpy and vacancy formation energy between the *i*-th and *j*-th elements. *n* is the number of elements. *T*_m_ is the melting point of the alloys, obtained by ${T}_{{{\rm{m}}}}={\sum }_{i=1}^{n}{c}_{{{\rm{i}}}}{T}_{{{\rm{m}}}{{\rm{i}}}}$. *T*_mi_ is the melting point of the i-th element. Δ*S*_mix_, Δ*H*_mix_, δΔ*H*_mix_, *Ω*, *E*_v_ are commonly used to predict the phases of HEAs. In this work, *Ω*^−1^ is used to avoid the not a number (NaN) values caused by Δ*H*_mix_ = 0. It should be noted that the binary vacancy formation energy for the *i*–*j* pair, ${E}_{{{\rm{ij}}}}^{{{\rm{v}}}}$, is calculated by Eqs. 8 and 9^71^:

8 $${E}_{{{\rm{ij}}}}^{{{\rm{v}}}}=(1-{f}_{{{\rm{ij}}}}){E}_{{{\rm{i}}}}^{{{\rm{v}}}}+{f}_{{{\rm{ij}}}}{E}_{{{\rm{j}}}}^{{{\rm{v}}}}{\left(\dfrac{{V}_{{{\rm{i}}}}}{{V}_{{{\rm{j}}}}}\right)}^{5/6}\quad \,{{\mbox{if}}}\,{V}_{{{\rm{i}}}} > {V}_{{{\rm{j}}}}$$

9 $${E}_{{{\rm{ij}}}}^{{{\rm{v}}}}=(1-{f}_{{{\rm{ij}}}}){E}_{{{\rm{j}}}}^{{{\rm{v}}}}+{f}_{{{\rm{ij}}}}{E}_{{{\rm{i}}}}^{{{\rm{v}}}}{\left(\dfrac{{V}_{{{\rm{j}}}}}{{V}_{{{\rm{i}}}}}\right)}^{5/6}\quad \,{\mbox{if}}\,{V}_{{{\rm{i}}}}\le {V}_{{{\rm{j}}}}$$

where *V*_*i*_ and *V*_*j*_ are the molar volumes of elements *i* and *j*, and ${E}_{{{\rm{i}}}}^{{{\rm{v}}}}$ and ${E}_{{{\rm{j}}}}^{{{\rm{v}}}}$ are the monovacancy formation energies of pure elements *i* and *j*, respectively. The weighting factor *f*_*ij*_ is defined as Eqs. 10 and 11:

10 $${f}_{{{\rm{ij}}}}={C}_{{{\rm{j}}}}\left(1+5{({C}_{{{\rm{i}}}}{C}_{{{\rm{j}}}})}^{2}\right)\quad \,{\mbox{if}}\,{V}_{i} > {V}_{j}$$

11 $${f}_{{{\rm{ij}}}}={C}_{{{\rm{i}}}}\left(1+5{({C}_{{{\rm{j}}}}{C}_{{{\rm{j}}}})}^{2}\right)\quad \,{\mbox{if}}\,{V}_{i}\le {V}_{j}$$

where the concentration weights are obtained by Eqs. 12 and 13:

12 $${C}_{{{\rm{i}}}}=\dfrac{{x}_{{{\rm{i}}}}{V}_{{{\rm{i}}}}^{{{\rm{2}}}/{{\rm{3}}}}}{{x}_{{{\rm{i}}}}{V}_{{{\rm{i}}}}^{{{\rm{2}}}/{{\rm{3}}}}+{x}_{{{\rm{j}}}}{V}_{{{\rm{j}}}}^{{{\rm{2}}}/{{\rm{3}}}}}$$

13 $${C}_{{{\rm{j}}}}=\dfrac{{x}_{{{\rm{j}}}}{V}_{{{\rm{j}}}}^{2/3}}{{x}_{{{\rm{i}}}}{V}_{{{\rm{i}}}}^{2/3}+{x}_{{{\rm{j}}}}{V}_{{{\rm{j}}}}^{2/3}}$$

where *x*_i_ and *x*_j_ are the atomic fractions of elements *i* and *j*. In addition, two descriptors related to plastic deformation behavior were included. The ratio *G*/*G*_0_ originates from the Kocks–Mecking model and is associated with dislocation dynamics^72^. *K*/*G* serves as a common criterion for predicting the plasticity of HEAs. *T*_b_ − *T*_m_ represents the range of liquid temperatures of alloys, which affects the liquid viscosity during solidification. Here, *G*, *G*_0_, *K*, and *T*_b_ denote the average values of shear modulus at room temperature, shear modulus at 0 K, bulk modulus at room temperature, and boiling point of the HEAs, respectively. They are included in the 46 elemental parameters (detailed below) and are obtained by applying the composition-weighted averaging rule.

2) Forty-six elemental parameters are also considered to calculate material descriptors by applying four commonly used formulas: weighted average value ($\overline{F}$), mismatch value (δ*F*), range value (Range *F*), and weighted average value considering binary pairs (Pairs *F*). These parameters are divided into four categories: (i) electronic properties (*F*_E_), including Pauling electronegativity, Allred Rochow electronegativity, valence electron concentration, free electron concentration, electronic work function, the first and second ionization energy, the numbers of valence electrons filled and unfilled in *s*, *p*, *d* orbitals and in all orbitals (the sum of the *s*, *p*, and *d* orbitals), Wigner lattice surface electron density, atomic number, group and period numbers from the periodic table; (ii) size properties (*F*_S_), including Waber metal radius, atomic radius, covalent radius, atomic volume, lattice constant; (iii) physical properties (*F*_P_), including body modulus, shear modulus, elastic modulus, relative atomic mass, molar volume, density, Poisson’s ratio, static dipole polarizabilities, specific heat, Brinell Hardness, thermal conductivity, electrical conductivity, shear modulus of 0 K temperature, viscosity of liquid metals; (iv) thermodynamic properties (*F*_T_), including melting temperature, boiling temperature, heat of vaporization, heat fusion, enthalpy of atomization, cohesive energy, vacancy migration energy, and vacancy formation energy^42^. The formulas are shown in Eqs. 14–17:

14 $$\overline{F}={\sum }_{i=1}^{n}{c}_{{{\rm{i}}}}{F}_{{{\rm{i}}}}$$

15 $${\rm\delta }F=\sqrt{{\sum}_{i=1}^{n}{c}_{{{\rm{i}}}}{(1-{F}_{{{\rm{i}}}}/\overline{F})}^{2}}$$

16 $${{\rm{Range}}}\,F=\max \left[{c}_{{{\rm{i}}}}{\left(1-\dfrac{{F}_{{{\rm{i}}}}}{\bar{F}}\right)}^{2}\right]-\min \left[{c}_{{{\rm{i}}}}{\left(1-\dfrac{{F}_{{{\rm{i}}}}}{\bar{F}}\right)}^{2}\right]$$

17 $${{\rm{Pairs}}}\,F=\dfrac{\mathop{\sum }_{i=1}^{n}\mathop{\sum }_{j=i}^{n}{c}_{{{\rm{i}}}}{c}_{{{\rm{j}}}}{F}_{{{\rm{ij}}}}}{\mathop{\sum }_{i=1}^{n}\mathop{\sum }_{j=i}^{n}{c}_{{{\rm{i}}}}{c}_{{{\rm{j}}}}}$$

where *F*_i_ and *c*_i_ are the element parameters and the mole fraction of i-th element, respectively. A full list of all 194 material descriptors is provided in Supplementary Table 2. The elemental properties and binary mixing enthalpies used in the calculations are provided in the GitHub repository (https://github.com/Wolido/hea_ducklake) and Supplementary Data 1.

The feature engineering and model development, including algorithm selection, hyperparameter optimization, and key descriptor identification, were established in our previous work^42^. The classification model was built using the GradientBoostingClassifier algorithm with hyperparameters set to n_estimators =  100 and random_state = 3. The training dataset comprised 92 compressive fracture plastic strain (*ε*_p_) values of as-cast BCC HEAs collected from published literature. This model can divide HEAs into three categories: low plasticity (*ε*_p_ < 10%), moderate plasticity (10% ≤ *ε*_p_ < 40%), and high plasticity (*ε*_p_ ≥ 40%). These categories are assigned labels of −1, 0, and 1, respectively. In the previous work, the dataset was split into training and testing sets with a ratio of 85%:15%. To fully utilize the limited data, the final model was retrained on the entire 92-sample dataset, achieving training and leave-one-out cross-validation accuracy (LOOCV) of 100% and 85.9%, respectively (Supplementary Fig. 1). A combination of material descriptors is used as input features, which are the average and mismatch values of valence electron concentration, the average value of atomic radius, the weighted average value considering binary pairs of covalent radius, and the mismatch values of atomic size *Λ*. To predict the plasticity of all HEAs in the database, the corresponding set of five material descriptors needs to be retrieved from the database. They are then normalized based on the minimum and maximum values of the training data. The normalized material descriptors are used as input features to the ML model for high-throughput prediction of plasticity labels across the composition space. The GB model is exported to ONNX format to enable efficient batch inference across the distributed computing cluster. The ONNX model, along with the normalization parameters and usage examples, is provided in the GitHub repository https://github.com/Wolido/hea_ducklake.

### Framework deployment on the HEA case study

The generation of element combinations and component proportions, being computationally lightweight, was handled through an automated iterative process rather than the parallel architecture. In contrast, the calculation of material descriptors was efficiently scaled using a Redis-based message queue system. The compositions of HEAs were enqueued as input, with the corresponding 194 material descriptors returned as output from the stateless Docker containers. This process was supported by 4 upstream and 4 downstream Redis queues, and utilized 500 CPU cores distributed across 10 compute nodes (each core running in a separate Docker container). The compute cluster comprised Intel Xeon Gold 6330 processors (56 physical cores per socket at 2.00 GHz; 50 cores utilized per node), 512 GB of RAM per node, and a 10 GbE interconnect providing over 9000 Mbps of internal bandwidth. Each queue managed all compositions from one HEA family, while each container processed a single HEA composition at a time. To further accelerate descriptor computation, NumPy’s broadcasting and Rust functions implemented via PyO3 were incorporated. For the AI-driven prediction stage, the metadata of HEA compositions were enqueued to retrieve the corresponding materials descriptors. These descriptors, along with the model predictions, served as the input and output of the AI-prediction containers. Given the optimized runtime, only one upstream and one downstream message queue were required in this stage. All generated data were persisted by Parquet format. The elemental combinations and component proportions were stored as two unified datasets, while the material descriptors and plasticity predictions were partitioned into multiple Parquet files organized by HEA families. The complete implementation of the HEA database construction pipeline is available in the Supplementary Information and https://github.com/Wolido/hea_ducklake. Both the data and code have been archived in Zenodo for long-term preservation and assigned the DOI 10.5281/zenodo.22075211 (https://doi.org/10.5281/zenodo.22075211^73^).

### Performance benchmark settings

Parallel computing performance tests were performed using 10 nodes with 56 cores per node, rather than the 50 cores per node used in database construction. This adjustment avoided CPU swapping that would otherwise arise when running Dask on partially allocated cores, introducing additional time overhead and compromising measurement accuracy. The Spark test used Apache Spark 3.5.8 with a mapPartitions architecture. Composition generation and descriptor computation were performed locally within each executor. The output was written as Parquet files compressed with Snappy, totaling 23 TB. Spark runs on the Java Virtual Machine, and each executor is a JVM process that manages task execution and data storage. The JVM heap stores task scheduling information, partition metadata, and serialization buffers. Each executor processed one full six-element family, corresponding to approximately 10.7 million compositions. We used the default Slurm configuration without I/O tuning, which is the typical setup in most research. Dask 2025.1.0 was applied to the performance test. The workload was partitioned into 101 groups of 50 families, each containing approximately 535 million compositions. Our single-family test was executed on Slurm 19.05.3-2, with all composition calculations evenly distributed across 560 CPU cores, generating 560 separate output files. For our framework, the same computational process described in Section “Framework Deployment on the HEA Case Study” was used, except that each worker pulled 200,000 HEA compositions per batch from the Redis queue instead of one composition at a time. Figure 4a reports the total completion time of the whole task and the peak memory consumption of a single representative CPU node. The memory behavior across all nodes in the cluster was nearly identical, so a single node was chosen for this metric. This is also supported by the runtime results of another node shown in the Supplementary Information, which exhibited similar memory consumption of approximately 147.4 GB when running our framework. In addition to the cluster-based performance evaluation, we tested our framework on two edge devices. A Raspberry Pi Zero 2W with an ARM Cortex-A53 processor and 512 MB RAM and a smartphone with an ARM Cortex-A520 processor and 16 GB RAM were used as compute nodes.

For the storage and query performance tests, the datasets were converted into CSV, JSON, and PostgreSQL formats to establish standard baselines. For the query performance tests, the CSV files were loaded using pandas, and queries were executed through DataFrame operations. For the PostgreSQL tests, queries were executed using standard SQL statements. For our framework, the database was accessed by downloading the metadata catalog, which is approximately several tens of MB in size. A DuckDB connection was then established, and the remote S3-compatible object storage was attached using the ATTACH command. SQL queries were executed directly against the virtual tables. The metadata catalog, query implementations, and usage examples are provided in the GitHub repository https://github.com/Wolido/hea_ducklake. We also prepared a specialized and unified test environment for the subset test cases, which was deployed on a cloud server with 4 cores, 16 GB of memory, 100 GB of hard drive, and a maximum bandwidth of 100 Mbps. The server was accessed from Beijing to Hong Kong. In contrast, the full-database cases were tested under a private network, not on the public network, because massive data transmission over the public network would cause prohibitive time consumption. It was conducted on a compute server with the same configuration as the descriptor-generation nodes. The full-database querying was not tested by CSV or PostgreSQL, owing to insufficient memory on the storage-side servers and query-side devices, respectively.

### Experimental methods

This subsection describes the experimental methods used to validate the AI-predicted HEA candidates. To maintain consistency with the as-cast HEA data used for training the AI model, the validation sample in this study is similarly prepared using a casting route. The selected HEA is synthesized via vacuum arc melting using high-purity metals (purity >99.9 wt%) and solidified in a water-cooled copper crucible. Each alloy ingot is remelted at least four times to ensure chemical homogeneity. After that, the alloy ingots are cut into several sections, followed by grinding and polishing for the next analysis. The crystal structure of the samples is examined by XRD (D/max-2550, CuK*α* radiation) over a 2*θ* range from 20^∘^ to 100^∘^ at a scanning rate of 4^∘^/min. Cylindrical compression samples (*ϕ*4 mm × 6 mm) were prepared to test their compression plasticity at room temperature, using an Instron 5985 universal testing machine with a strain rate of 1 × 10^−3^ s^−1^. Compositional characterization was performed using a Zeiss Sigma 500 scanning electron microscope equipped with an Oxford UltimMax 65 EDS detector.

## Supplementary information

Supplementary Information

Description of Additional Supplementary File

Supplementary Data 1

Transparent Peer Review File

## Source data

Source Data

## Data Availability

The elemental property data used in this study and the HEA composition, descriptor, and prediction datasets generated in this study have been deposited in the Zenodo repository under accession code 10.5281/zenodo.22075211 (https://doi.org/10.5281/zenodo.22075211^73^) and are also available in the GitHub repository https://github.com/Wolido/hea_ducklake. All data are publicly available with no access restrictions. Source data are provided as a Source Data file. Source data are provided with this paper.

## Code availability

The custom code developed for this study is available in the HEA DuckLake GitHub repository (https://github.com/Wolido/hea_ducklake) and has been archived in Zenodo with the version-specific DOI 10.5281/zenodo.22075211 (https://doi.org/10.5281/zenodo.22075211^73^). The archived release v1.0.0 corresponds to the code version used in this paper. All code is publicly available under the Creative Commons Attribution 4.0 International licence (CC-BY-4.0).
