# Supplementary material for: Trillion-scale integrated framework for high-throughput materials databases and seamless sharing
Source: Nat Commun. 2026 Oct 7;17:11401. doi: 10.1038/s41467-026-78223-3 (PMC13736967; doi:10.1038/s41467-026-78223-3)
Supplement: Supplementary file 1 [file 41467_2026_78223_MOESM1_ESM.pdf]

Trillion-Scale Integrated Framework for High-Throughput Materials Databases and Seamless Sharing

Xiaoya Huang[1,2,3], Yu Liu[1,3,4,5*], Shuo Shi[1], Yuanyuan Zhang[4], Zengzeng Liang[4,5], Miao Zhou[1,2,6], Hanwei Fu[4], Lei Zheng[1,3,4*], Peng Kang[1,3,4*]

[1]Tianmushan Laboratory, Beihang University, Hangzhou, 311115, China.
[2]School of Physics, Beihang University, Beijing, 100191, China.
[3]State Key Laboratory of Artificial Intelligence for Materials Science, Beihang University, Beijing, 102206, China.
[4]School of Materials Science and Engineering, Beihang University, Beijing, 100191, China.
[5]Testing and Evaluation Center for High-Performance Fibers, Beihang University, Beijing, 102206, China.
[6]Beihang Hangzhou Innovation Institute Yuhang, Hangzhou, 310023, China.

*Corresponding author(s). E-mail(s): liuyucarbon@buaa.edu.cn; zhenglei@buaa.edu.cn; pengkang@buaa.edu.cn;

Supplementary Note 1. Implementation and operational specifications of the stateless parallel computation framework

Supplementary Note 1.1. Core logic for applying the stateless parallel framework

The framework is designed for computational tasks that can be partitioned into independent and repetitive computational units. The core execution logic is to package the subtask computations into Docker containers, and to push the required input

data into Redis for scheduling management. Unlike routine execution without the framework, using our framework requires modifications to the computation code to include connecting to Redis, retrieving subtask data from the message queue, and writing the results to the message queue. Redis serves as a temporary queue on a server and must be deployed first. A separate program is then needed to push input data into Redis, and another program is required for result collection.

The Redis deployment, subtask push, and result collection steps are lightweight and can be run either in Docker or directly on the server. The subtask computations themselves are recommended to be run in Docker to ensure fast deployment and consistent environments. A single Redis queue can become an I/O bottleneck under extremely high throughput. This can be mitigated by using multiple parallel queues. In this setup, worker nodes remain stateless and pull tasks from all available queues, but users need to define their own data aggregation logic to combine results from multiple queues.

Supplementary Note 1.2. idm-gridcore: framework implementation and usage

The repository https://github.com/Wolido/idm-gridcore provides a general-purpose implementation of the stateless parallel computing architecture shown in Figure 2a. It consists of three coordinated components. ComputeHub is the central scheduler. It maintains the task registry and the list of online worker nodes, exposes a small web interface, and drives task switching. A task is registered with a JSON payload that specifies the Docker image and the input and output Redis queues. The administrator advances the workflow by requesting a task transition through the appropriate endpoint. GridNode runs on each worker machine. It registers itself with ComputeHub on startup, sends periodic heartbeats, and launches one Docker container per central processing unit core. Each container receives the Redis connection parameters and queue names as environment variables, retrieves one task at a time from the input queue, performs the computation, and writes the result to the output queue. The repository includes the complete source code for ComputeHub and GridNode, a Docker Compose template for Redis, cross-platform build scripts, example configuration files, and a minimal working example (https://github.com/Wolido/idm-gridcore/tree/master/test-workflow) that computes the squares of one million numbers in parallel.

Supplementary Note 1.3. agent-idm-gridcore: AI-agent skill for automated deployment and use

The repository https://github.com/Wolido/agent-idm-gridcore provides an AI-agent skill that wraps idm-gridcore. It allows users to deploy and use the distributed computing framework through natural-language dialogue rather than manual configuration. Given a description of a computational task, the agent checks the environment, deploys a cluster if needed, generates the consumer code and Dockerfile, registers the task with ComputeHub, pushes data to Redis, monitors execution, and returns the results. The repository also includes installation instructions and usage guidelines for

the skill. Direct use of idm-gridcore remains possible for users who prefer manual control.

Supplementary Note 2. Technical Terminology Used in This Work

A glossary of key technical terms used in this work, along with their definitions and roles in the framework, is provided in Supplementary Table 1.

Supplementary Note 3. Data and executable code for the HEA case study

The repository (https://github.com/Wolido/hea_ducklake) contains the source code for constructing the senary HEA database, the metadata catalog, and usage examples. A reproducible workflow example is also provided at https://github.com/Wolido/hea_ducklake/tree/main/examples/minimal_workflow. This workflow executes a small-scale task on 100 randomly sampled HEA compositions, covering the entire process from Redis queue setup and Docker container orchestration to distributed descriptor-calculation workloads and dataset storage and management. The workflow is documented in the "README.md".

Supplementary Note 4. The machine learning model for predicting the plasticity of high entropy alloys

The GB model used in this work were retrained using the whole 92 data with the training and leave-one-out cross-validation (LOOCV) accuracy of 100% and 85.9%, respectively (Supplementary Figure 1).

Supplementary Note 5. The materials descriptors of HEAs

Here, we supplied the entire table of 194 materials descriptors, as shown in Supplementary Table 2.

Supplementary Note 6. The large-scale databases of HEAs

The datasets of elemental combinations, the proportions of components, 194 materials descriptors, and the predicted plasticity of HEAs are shown in Supplementary Figures 2–5.

Supplementary Table 1: Glossary of technical terms used in the integrated framework

Term	Definition	Role in the framework
Stateless parallel computing	A computing paradigm where each worker processes tasks independently without retaining any state between tasks.	Enables dynamic scaling and fault tolerance by decoupling task execution from state management.
Message queue	An asynchronous communication mechanism that decouples data producers from consumers.	In our framework, Redis List implements the message queue for task distribution and results collection.
Redis List	A first-in-first-out (FIFO) queue data structure provided by Redis.	Serves as upstream and downstream task queues in the parallel architecture.
Docker container	An isolated runtime environment that packages an application and its dependencies.	Runs as a stateless worker executing computational tasks.
Containerization	A technology that packages applications and dependencies into isolated containers.	Ensures environment consistency across heterogeneous computing nodes.
Worker	A computational unit (process or container) that fetches and executes tasks.	In our framework, workers are implemented as Docker containers that consume tasks from Redis queues.
Write Lock	A synchronization mechanism that controls write operations.	Prevents queue congestion when producers generate tasks faster than consumers can process.
Lakehouse architecture	An integrated data platform that combines the scalable storage of data lakes with the query capabilities of data warehouses.	Serves as the foundation of the data management module.
DuckLake	A metadata-driven data sharing layer built on Parquet files in object storage.	Enables remote dataset access via a small metadata catalog, avoiding full data transfer.
Metadata catalog	A structured record of dataset schema, file locations, and partitions.	Allows local querying without transferring entire datasets.
Object storage	A storage architecture that manages data as objects with unique identifiers.	Provides durable, scalable cloud storage for Parquet files.
S3 protocol	A RESTful API standard for object storage (Amazon S3 and compatible systems).	Enables HTTP-based remote access to shared databases.
Cloud sharing	Distribution of datasets and services via cloud infrastructure.	Enables seamless sharing of the HEA database among researchers.
Parquet	A columnar storage format with built-in schema and compression.	Stores HEA datasets and supports fast column retrieval.
Columnar storage	A storage layout where column values are stored contiguously.	Matches descriptor query patterns by reading only the required columns.
DuckDB	An in-process analytical database engine.	Queries Parquet files directly without requiring a separate database server.
Embedded analytical engine	A database engine that runs within the application process.	DuckDB uses this model to query Parquet files directly.
Query	A request to retrieve or analyze data.	Supports full database scans, column queries, and reverse queries on HEA data.
Reverse query	A query that finds records whose descriptor values match specified conditions.	Enables searching for HEA compositions within target descriptor ranges.
I/O (Input/Output)	Data transfer between the system and external storage or devices.	Please provide the role description for I/O.

Supplementary Table 2: The knowledge-based feature set.

Description	Features		Formulas
Plastic descriptors	Vacancy formation energy 1	E_v	$4\sum c_i c_j E_{ij}^v$
	Dislocation storage/dynamic coefficient	G/G_0	G/G_0
	Pugh's modulus ratio	K/G	K/G
	Topology atomic size mismatch	γ	$\gamma = (1-\sqrt{\frac{(r_s+\bar{r})^2-\bar{r}^2}{(r_s+\bar{r})^2}})/(1-\sqrt{\frac{(r_l+\bar{r})^2-\bar{r}^2}{(r_l+\bar{r})^2}})$
	Liquid Range	$T_b - T_m$	$T_b - T_m$
Phase parameters	Mixing entropy	ΔS_{mix}	$-R\sum c_i \ln c_i$
	Mixing enthalpy	ΔH_{mix}	$4\sum c_i c_j H_{ij}^{mix}$
	Mismatch of mixing enthalpy	$\delta\Delta H_{mix}$	$\sqrt{\sum c_i c_j (1-\frac{H_{ij}^{mix}}{\Delta H_{mix}})}$
	Formation capability of solid solution phase	Ω^{-1}	$\frac{1000\lvert\Delta H_{mix}\rvert}{T_m \Delta S_{mix}}$
	Geometrical descriptor	Λ	$\Delta S_{mix}/\delta R_m^2$
Electronic F_E	Pauling electronegativity	F_{E1}	
	Allred Rochow electronegativity	F_{E2}	
	Valence electron concentration VEC*	F_{E3}	
	Free electron concentration	F_{E4}	
	Electron work function	F_{E5}	
	First Ionization Energy	F_{E6}	
	Second Ionization Energy	F_{E7}	
	Number of VECs filled valence orbitals	F_{E8}	
	Number of VECs valence electrons unfilled valence orbitals	F_{E9}	
	Number of VECs filled s valence orbitals	F_{E10}	
	Number of VECs unfilled s valence orbitals	F_{E11}	
	Number of VECs filled p valence orbitals	F_{E12}	
	Number of VECs unfilled p valence orbitals	F_{E13}	
	Number of VECs filled d valence orbitals	F_{E14}	
	Number of VECs unfilled d valence orbitals	F_{E15}	
	Electron density at surface of Wigner Seitz cell	F_{E16}	
	Atomic number	F_{E17}	
	Group	F_{E18}	
	Period	F_{E19}	
Physical F_P	Bulk modulus K	F_{P1}	$\overline{F} = \sum c_i F_i$
	Shear modulus G	F_{P2}	
	Young's modulus	F_{P3}	$\delta F = \sqrt{\sum c_i (1-F_i/\overline{F})^2}$
	Atomic Mass	F_{P4}	
	Molar volume	F_{P5}	
	Density	F_{P6}	$\Delta F = \max[c_i(1-\frac{F_i}{\overline{F}})^2] - \min[c_i(1-\frac{F_i}{\overline{F}})^2]$
	Poisson's ratio	F_{P7}	
	Static dipole polarizabilities	F_{P8}	
	Specific heat capacity	F_{P9}	Pairs $F = \frac{\sum_i \sum_j c_i c_j F_{ij}}{\sum_i \sum_j c_i c_j}$
	Brinell Hardness	F_{P10}	
	Thermal Conductivity	F_{P11}	
	Electrical Conductivity	F_{P12}	
	0 K Shear modulus G_0	F_{P13}	
	Viscosity of Liquid metals	F_{P14}	
Size F_S	Metallic radius R_m	F_{S1}	
	Covalent radius	F_{S2}	
	Atomic radius	F_{S3}	
	Atomic volume	F_{S4}	
Thermodynamic F_T	Lattice constant (BCC, a)	F_{S5}	
	Melting temperature T_m	F_{T1}	
	Boiling temperature T_b	F_{T2}	
	Heat of vaporization	F_{T3}	
	Heat of fusion	F_{T4}	
	Enthalpy of atomisation	F_{T5}	
	Cohesive energy	F_{T6}	
	Vacancy migration energy	F_{T7}	
	Vacancy formation energy 2	F_{T8}	

c_i, c_j and F_i, F_j are the content and the corresponding elemental parameter of the i-th and j-th component of a HEA; the parameters G, G_0, K, a, T_b and T_m are the average values. Two vacancy formation energies are included. r_s is the smallest atomic size among the principal elements and r_l is the largest atomic size. VECs are the valence electrons.

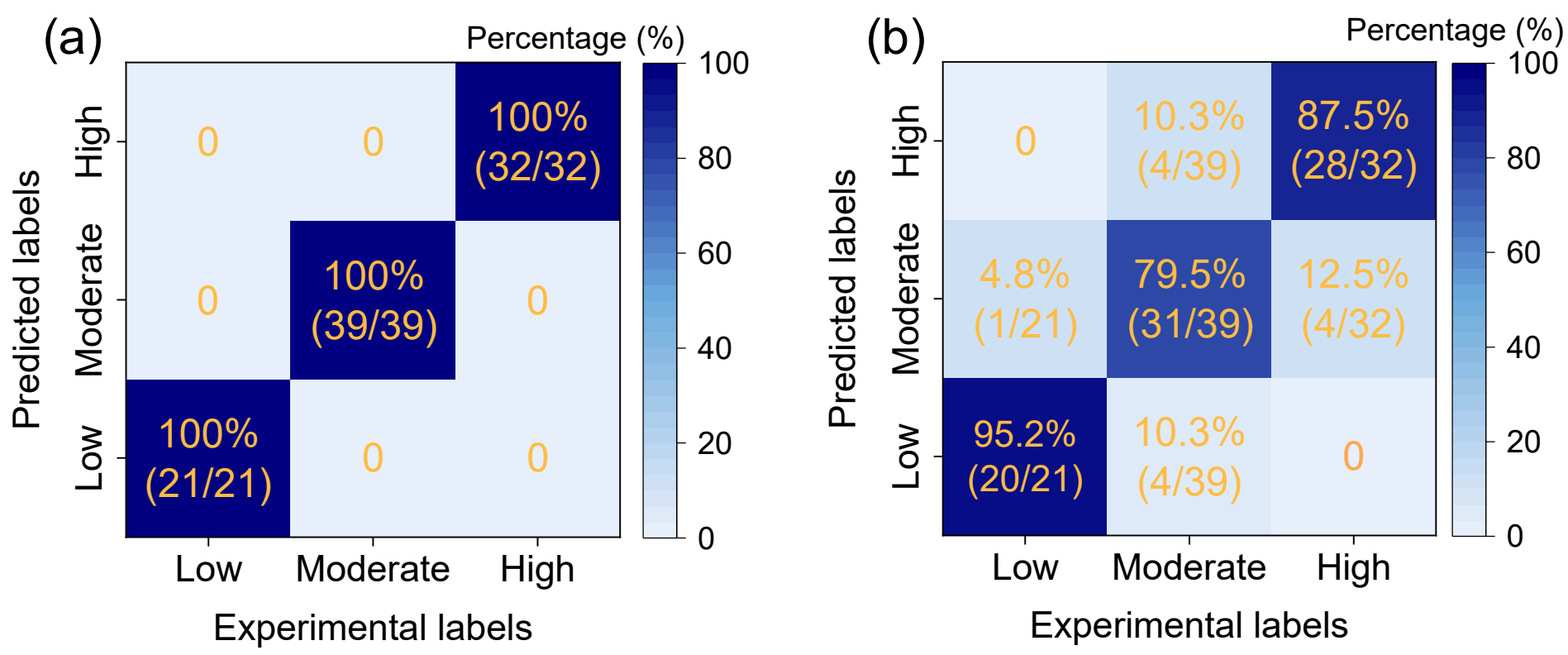

Supplementary Figure 1: Prediction performance of the retrained GB model. (a) Training confusion matrix; (b) LOOCV confusion matrix. Source data are provided as a Source Data file.

Supplementary Note 7. Performance Benchmark Details

The memory consumption trajectories of Spark, Dask, Slurm, and our framework during the HEA descriptor computation are shown in Supplementary Figure 6.

Supplementary Note 8. Supplementary experimental results

As shown in Supplementary Figure 7 and 8, for each HEA, the compression test was repeated three times using independent specimens to ensure reproducibility. EDS mapping was performed on three distinct regions per composition, and the results were listed in Supplementary Table 3.

Supplementary references

id<br>int32	elem1<br>varchar	elem2<br>varchar	elem3<br>varchar	elem4<br>varchar	elem5<br>varchar	elem6<br>varchar
1	Fe	Ni	Mn	Al	Cr	Cu
2	Fe	Ni	Mn	Al	Cr	Co
3	Fe	Ni	Mn	Al	Cr	Mo
4	Fe	Ni	Mn	Al	Cr	Ti
5	Fe	Ni	Mn	Al	Cr	Nb
6	Fe	Ni	Mn	Al	Cr	Ta
7	Fe	Ni	Mn	Al	Cr	W
8	Fe	Ni	Mn	Al	Cr	V
9	Fe	Ni	Mn	Al	Cr	Zr
10	Fe	Ni	Mn	Al	Cr	Hf
11	Fe	Ni	Mn	Al	Cu	Co
12	Fe	Ni	Mn	Al	Cu	Mo
13	Fe	Ni	Mn	Al	Cu	Ti
14	Fe	Ni	Mn	Al	Cu	Nb
15	Fe	Ni	Mn	Al	Cu	Ta
16	Fe	Ni	Mn	Al	Cu	W
17	Fe	Ni	Mn	Al	Cu	V
18	Fe	Ni	Mn	Al	Cu	Zr
19	Fe	Ni	Mn	Al	Cu	Hf
20	Fe	Ni	Mn	Al	Co	Mo
.	.	.	.	.	.	.
.	.	.	.	.	.	.
.	.	.	.	.	.	.
4986	Mo	Ti	Nb	W	Zr	Hf
4987	Mo	Ti	Nb	V	Zr	Hf
4988	Mo	Ti	Ta	W	V	Zr
4989	Mo	Ti	Ta	W	V	Hf
4990	Mo	Ti	Ta	W	Zr	Hf
4991	Mo	Ti	Ta	V	Zr	Hf
4992	Mo	Ti	W	V	Zr	Hf
4993	Mo	Nb	Ta	W	V	Zr
4994	Mo	Nb	Ta	W	V	Hf
4995	Mo	Nb	Ta	W	Zr	Hf
4996	Mo	Nb	Ta	V	Zr	Hf
4997	Mo	Nb	W	V	Zr	Hf
4998	Mo	Ta	W	V	Zr	Hf
4999	Ti	Nb	Ta	W	V	Zr
5000	Ti	Nb	Ta	W	V	Hf
5001	Ti	Nb	Ta	W	Zr	Hf
5002	Ti	Nb	Ta	V	Zr	Hf
5003	Ti	Nb	W	V	Zr	Hf
5004	Ti	Ta	W	V	Zr	Hf
5005	Nb	Ta	W	V	Zr	Hf

5005 rows (40 shown) 7 columns

Supplementary Figure 2: The datasets of elemental combinations.

id int32	con1 float	con2 float	con3 float	con4 float	con5 float	con6 float
1	0.05	0.05	0.05	0.15	0.35	0.35
2	0.05	0.05	0.05	0.16	0.34	0.35
3	0.05	0.05	0.05	0.16	0.35	0.34
4	0.05	0.05	0.05	0.17	0.33	0.35
5	0.05	0.05	0.05	0.17	0.34	0.34
6	0.05	0.05	0.05	0.17	0.35	0.33
7	0.05	0.05	0.05	0.18	0.32	0.35
8	0.05	0.05	0.05	0.18	0.33	0.34
9	0.05	0.05	0.05	0.18	0.34	0.33
10	0.05	0.05	0.05	0.18	0.35	0.32
11	0.05	0.05	0.05	0.19	0.31	0.35
12	0.05	0.05	0.05	0.19	0.32	0.34
13	0.05	0.05	0.05	0.19	0.33	0.33
14	0.05	0.05	0.05	0.19	0.34	0.32
15	0.05	0.05	0.05	0.19	0.35	0.31
16	0.05	0.05	0.05	0.2	0.3	0.35
17	0.05	0.05	0.05	0.2	0.31	0.34
18	0.05	0.05	0.05	0.2	0.32	0.33
19	0.05	0.05	0.05	0.2	0.33	0.32
20	0.05	0.05	0.05	0.2	0.34	0.31
.	.	.	.	.	.	.
.	.	.	.	.	.	.
.	.	.	.	.	.	.
10762628	0.35	0.35	0.12	0.05	0.05	0.08
10762629	0.35	0.35	0.12	0.05	0.06	0.07
10762630	0.35	0.35	0.12	0.05	0.07	0.06
10762631	0.35	0.35	0.12	0.05	0.08	0.05
10762632	0.35	0.35	0.12	0.06	0.05	0.07
10762633	0.35	0.35	0.12	0.06	0.06	0.06
10762634	0.35	0.35	0.12	0.06	0.07	0.05
10762635	0.35	0.35	0.12	0.07	0.05	0.06
10762636	0.35	0.35	0.12	0.07	0.06	0.05
10762637	0.35	0.35	0.12	0.08	0.05	0.05
10762638	0.35	0.35	0.13	0.05	0.05	0.07
10762639	0.35	0.35	0.13	0.05	0.06	0.06
10762640	0.35	0.35	0.13	0.05	0.07	0.05
10762641	0.35	0.35	0.13	0.06	0.05	0.06
10762642	0.35	0.35	0.13	0.06	0.06	0.05
10762643	0.35	0.35	0.13	0.07	0.05	0.05
10762644	0.35	0.35	0.14	0.05	0.05	0.06
10762645	0.35	0.35	0.14	0.05	0.06	0.05
10762646	0.35	0.35	0.14	0.06	0.05	0.05
10762647	0.35	0.35	0.15	0.05	0.05	0.05
10762647 rows (10.76 million rows, 40 shown)						7 columns

Supplementary Figure 3: The datasets of the proportions of component.

con_index int32	ave_fe1 float	ave_fe2 float	ave_fe3 float	ave_fe4 float	ave_fe5 float	ave_fe6 float	ave_fe7 float	ave_fe8 float	ave_fe9 float	…	pair_sfe4 float	smix_data float	lambda_data float	gamma_data float	ev_data float	gg0_data float	kg_data float	tbtm_data float	hmix_data float	rmse_hmix_data float	omega_data float
1	1.532	1.3385	4.75	1.2535	6.8971	14.416	6.85	7.25	1.85	…	0.0	12.21163	1393.1959	0.75949	2.93877	0.84637	3.07407	2036.35	-3.22	0.71795	0.11016
2	1.5307	1.3373	4.75	1.2602	6.9085	14.4249	6.99	7.25	1.85	…	0.0	12.27816	1415.3835	0.75955	2.96728	0.84691	3.06822	2045.79	-3.1476	0.7409	0.10661
3	1.5337	1.3396	4.76	1.2543	6.9096	14.4399	7.0	7.24	1.85	…	0.0	12.27816	1420.5486	0.75938	2.95276	0.84672	3.07455	2035.22	-3.1512	0.73243	0.1067
4	1.5294	1.3361	4.75	1.2669	6.9199	14.4338	7.13	7.25	1.85	…	0.0	12.33705	1437.3104	0.75962	2.99313	0.84745	3.06243	2055.23	-3.0744	0.76476	0.10315
5	1.5324	1.3384	4.76	1.261	6.921	14.4488	7.14	7.24	1.85	…	0.0	12.3395	1442.3951	0.75944	2.97928	0.84726	3.06874	2044.66	-3.0812	0.75529	0.10334
6	1.5354	1.3407	4.77	1.2551	6.9221	14.4637	7.15	7.23	1.85	…	0.0	12.33705	1448.4607	0.75926	2.96417	0.84707	3.07501	2034.09	-3.0848	0.74638	0.10346
7	1.5281	1.3349	4.75	1.2736	6.9313	14.4428	7.27	7.25	1.85	…	0.0	12.38853	1459.0022	0.75969	3.01633	0.84799	3.05668	2064.67	-3.0004	0.78963	0.0998
8	1.5311	1.3372	4.76	1.2677	6.9324	14.4577	7.28	7.24	1.85	…	0.0	12.39349	1464.0005	0.75951	3.00319	0.8478	3.06298	2054.1	-3.0104	0.77906	0.10007
9	1.5341	1.3395	4.77	1.2618	6.9336	14.4726	7.29	7.23	1.85	…	0.0	12.39349	1469.9927	0.75933	2.98876	0.84761	3.06925	2043.53	-3.0172	0.76911	0.10027
10	1.5371	1.3418	4.78	1.2559	6.9347	14.4875	7.3	7.22	1.85	…	0.0	12.38853	1477.0298	0.75915	2.97315	0.84742	3.07547	2032.96	-3.0208	0.75975	0.10041
11	1.5268	1.3337	4.75	1.2803	6.9427	14.4517	7.41	7.25	1.85	…	0.0	12.43278	1480.4808	0.75976	3.03692	0.84852	3.05099	2074.11	-2.9256	0.81565	0.09652
12	1.5298	1.336	4.76	1.2744	6.9438	14.4666	7.42	7.24	1.85	…	0.0	12.44035	1485.3872	0.75958	3.02452	0.84833	3.05728	2063.54	-2.9388	0.80386	0.09688
13	1.5328	1.3383	4.77	1.2685	6.945	14.4815	7.43	7.23	1.85	…	0.0	12.44287	1491.3003	0.7594	3.01081	0.84814	3.06353	2052.97	-2.9488	0.79277	0.09716
14	1.5358	1.3406	4.78	1.2626	6.9461	14.4965	7.44	7.22	1.85	…	0.0	12.44035	1498.271	0.75922	2.99587	0.84795	3.06975	2042.4	-2.9556	0.78233	0.09739
15	1.5388	1.3429	4.79	1.2567	6.9472	14.5114	7.45	7.21	1.85	…	0.0	12.43278	1506.3572	0.75904	2.9798	0.84776	3.07593	2031.83	-2.9592	0.77249	0.09754
16	1.5255	1.3325	4.75	1.287	6.9541	14.4606	7.55	7.25	1.85	…	0.0	12.46998	1501.7648	0.75983	3.05493	0.84905	3.04535	2083.55	-2.85	0.84295	0.09332
17	1.5285	1.3348	4.76	1.2811	6.9553	14.4755	7.56	7.24	1.85	…	0.0	12.48022	1506.5747	0.75965	3.0433	0.84886	3.05163	2072.98	-2.8664	0.82981	0.09376
18	1.5315	1.3371	4.77	1.2752	6.9564	14.4904	7.57	7.23	1.85	…	0.0	12.48534	1512.4037	0.75947	3.03034	0.84867	3.05787	2062.41	-2.8796	0.81746	0.09413
19	1.5345	1.3394	4.78	1.2693	6.9575	14.5054	7.58	7.22	1.85	…	0.0	12.48534	1519.3022	0.75929	3.01612	0.84847	3.06407	2051.84	-2.8896	0.80584	0.09444
20	1.5375	1.3417	4.79	1.2634	6.9586	14.5203	7.59	7.21	1.85	…	0.0	12.48022	1527.3287	0.75911	3.00074	0.84828	3.07024	2041.27	-2.8964	0.79489	0.09468
⋮	⋮	⋮	⋮	⋮	⋮	⋮	⋮	⋮	⋮	⋮	⋮	⋮	⋮	⋮	⋮	⋮	⋮	⋮	⋮	⋮	⋮
10762628	1.7919	1.3852	5.62	1.2096	6.9241	15.7082	6.32	6.38	1.18	…	0.0	12.39566	2185.6587	0.75394	2.80459	0.58719	3.01854	1646.26	-5.0764	0.51171	0.16057
10762629	1.7949	1.3875	5.63	1.2037	6.9253	15.7232	6.33	6.37	1.18	…	0.0	12.4215	2335.1572	0.75375	2.82378	0.58754	3.02362	1635.69	-4.8876	0.51649	0.15424
10762630	1.7979	1.3898	5.64	1.1978	6.9264	15.7381	6.34	6.36	1.18	…	0.0	12.4215	2507.2312	0.75356	2.84275	0.58788	3.02867	1625.12	-4.6956	0.52093	0.14815
10762631	1.8009	1.3921	5.65	1.1919	6.9275	15.753	6.35	6.35	1.18	…	0.0	12.39566	2708.8535	0.75337	2.86187	0.58823	3.0337	1614.55	-4.5004	0.52492	0.14226
10762632	1.7936	1.3863	5.63	1.2104	6.9367	15.7321	6.47	6.37	1.18	…	0.0	12.4215	2326.3745	0.75382	2.83428	0.5884	3.01924	1645.13	-5.0032	0.50788	0.15721
10762633	1.7966	1.3886	5.64	1.2045	6.9378	15.747	6.48	6.36	1.18	…	0.0	12.43542	2497.6575	0.75363	2.85408	0.58874	3.02429	1634.56	-4.8144	0.51185	0.15107
10762634	1.7996	1.3909	5.65	1.1986	6.9389	15.7619	6.49	6.35	1.18	…	0.0	12.4215	2697.34375	0.75344	2.87392	0.58908	3.02931	1623.99	-4.6224	0.51536	0.14518
10762635	1.7953	1.3874	5.64	1.2112	6.9492	15.7559	6.62	6.36	1.18	…	0.0	12.4215	2483.367	0.7537	2.86316	0.58959	3.01993	1644.0	-4.9324	0.50296	0.15428
10762636	1.7983	1.3897	5.65	1.2053	6.9503	15.7708	6.63	6.35	1.18	…	0.0	12.4215	2681.2944	0.75351	2.88386	0.58993	3.02495	1633.43	-4.7436	0.50602	0.14835
10762637	1.797	1.3885	5.65	1.212	6.9617	15.7798	6.77	6.35	1.18	…	0.0	12.39566	2660.7656	0.75358	2.89176	0.59078	3.02062	1642.87	-4.864	0.49686	0.15178
10762638	1.7946	1.3853	5.63	1.2079	6.9254	15.7196	6.33	6.37	1.17	…	0.0	12.35315	2313.9158	0.75382	2.81812	0.58729	3.02979	1643.39	-5.0144	0.50717	0.15877
10762639	1.7976	1.3876	5.64	1.202	6.9265	15.7346	6.34	6.36	1.17	…	0.0	12.36708	2484.3442	0.75363	2.83816	0.58764	3.03483	1632.82	-4.826	0.5111	0.1526
10762640	1.8006	1.3899	5.65	1.1961	6.9276	15.7495	6.35	6.35	1.17	…	0.0	12.35315	2683.011	0.75344	2.85813	0.58798	3.03986	1622.25	-4.6344	0.51456	0.14667
10762641	1.7963	1.3864	5.64	1.2087	6.9379	15.7435	6.48	6.36	1.17	…	0.0	12.36708	2472.8896	0.7537	2.84883	0.58849	3.03042	1642.26	-4.944	0.50229	0.15565
10762642	1.7993	1.3887	5.65	1.2028	6.939	15.7584	6.49	6.35	1.17	…	0.0	12.36708	2670.041	0.75351	2.86962	0.58884	3.03544	1631.69	-4.7556	0.50531	0.14969
10762643	1.798	1.3875	5.65	1.2095	6.9504	15.7673	6.63	6.35	1.17	…	0.0	12.35315	2652.1218	0.75358	2.87897	0.58969	3.03105	1641.13	-4.876	0.49625	0.15299
10762644	1.7973	1.3854	5.64	1.2062	6.9266	15.731	6.34	6.36	1.16	…	0.0	12.29233	2458.3442	0.7537	2.83202	0.58739	3.04102	1640.52	-4.9556	0.50141	0.1573
10762645	1.8003	1.3877	5.65	1.2003	6.9277	15.746	6.35	6.35	1.16	…	0.0	12.29233	2654.395	0.75351	2.85298	0.58774	3.04603	1629.95	-4.7676	0.5044	0.1513
10762646	1.799	1.3865	5.65	1.207	6.9392	15.7549	6.49	6.35	1.16	…	0.0	12.29233	2639.5413	0.75358	2.86384	0.58859	3.04158	1639.39	-4.888	0.49542	0.15445
10762647	1.8	1.3855	5.65	1.2045	6.9279	15.7424	6.35	6.35	1.15	…	0.0	12.21163	2622.6897	0.75358	2.84633	0.58749	3.05223	1637.65	-4.9	0.49439	0.15618

10762647 rows (10.76 million rows, 40 shown) 195 columns (21 shown)

Supplementary Figure 4: 194 materials descriptors of AlCrCuCoFeNi HEAs.

con_index int64	pred_data int64
1	0
2	0
3	0
4	1
5	1
6	1
7	1
8	1
9	1
10	1
11	1
12	1
13	1
14	1
15	1
16	1
17	1
18	1
19	1
20	1
.	.
.	.
.	.
10762628	1
10762629	1
10762630	1
10762631	1
10762632	1
10762633	1
10762634	1
10762635	1
10762636	1
10762637	1
10762638	1
10762639	1
10762640	1
10762641	1
10762642	1
10762643	1
10762644	1
10762645	1
10762646	1
10762647	1
10762647 rows (10.76 million rows) (40 shown)	

Supplementary Figure 5: The prediction results of AlCrCuCoFeNi HEAs.

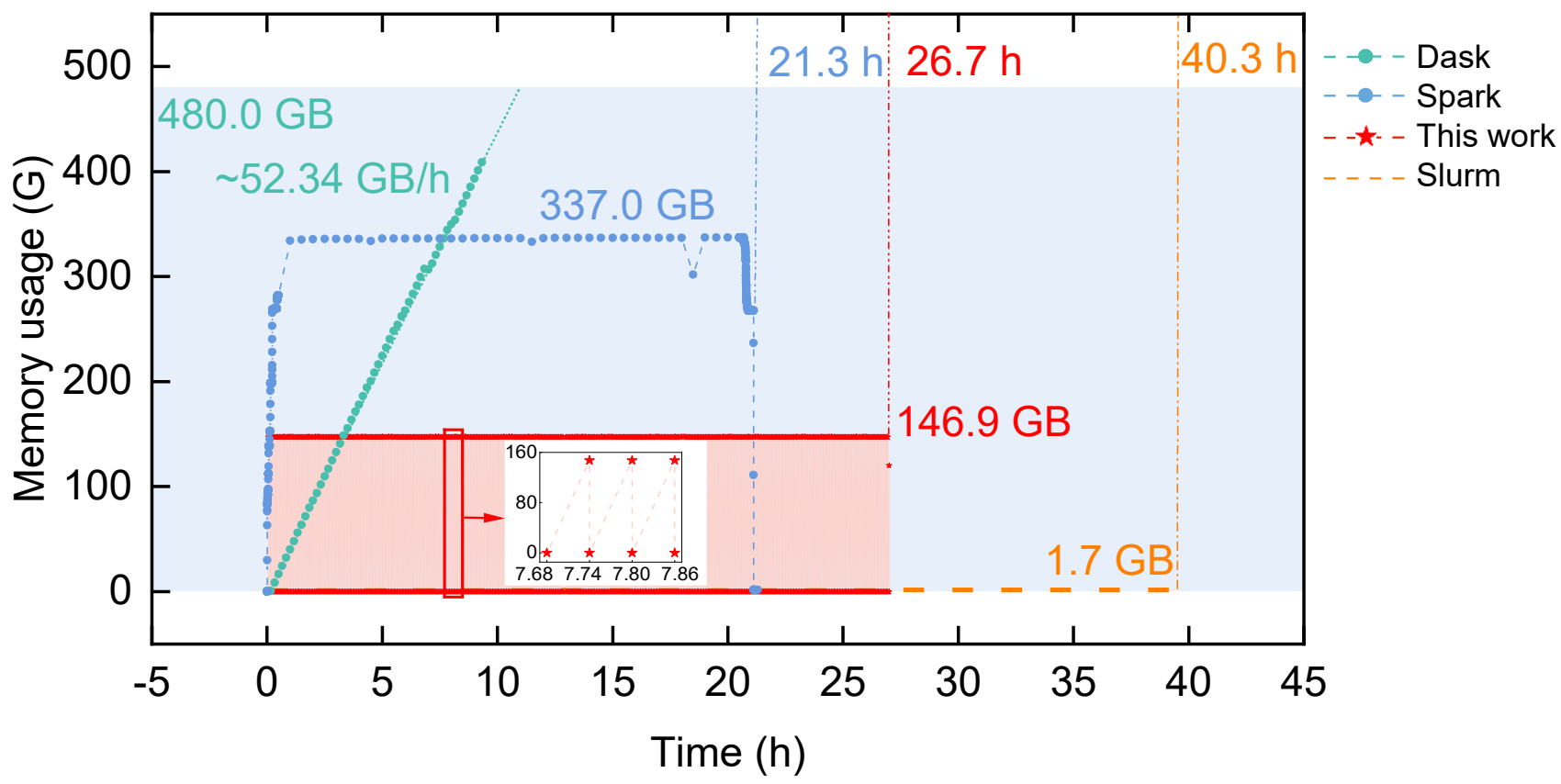

Supplementary Figure 6: Memory usage per compute node over runtime for Spark, Dask, Slurm, and our framework during the HEA descriptor computation. The embedded zoomed-in panel displays our framework's memory behavior across three Docker worker runtime cycles. Source data are provided as a Source Data file.

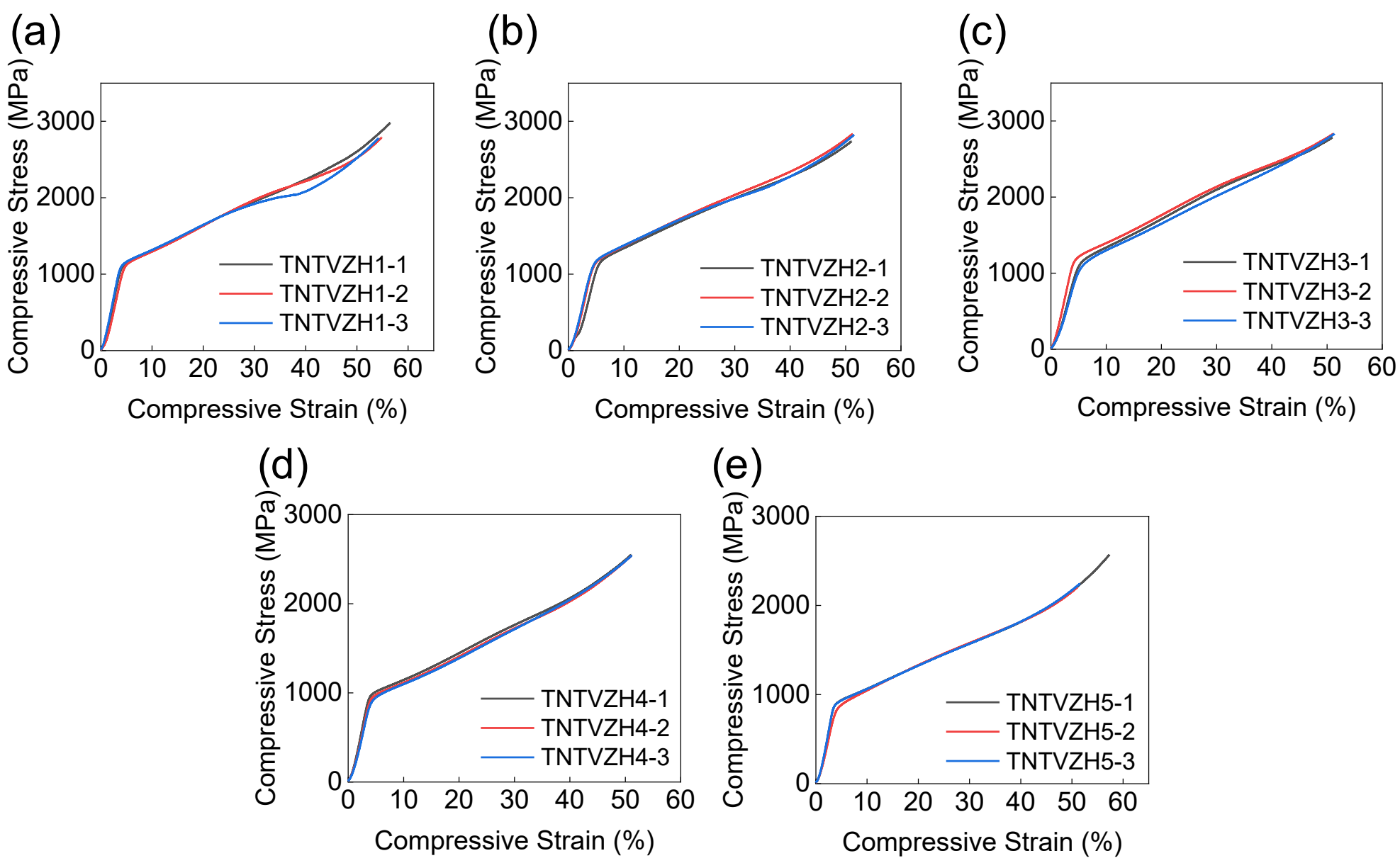

Supplementary Figure 7: Compressive stress–strain curves for the Ti–Nb–Ta–V–Zr–Hf (TNTVZH) high-entropy alloys. Subfigures (a)–(e) show the results for compositions TNTVZH 1 through 5, respectively. Source data are provided as a Source Data file.

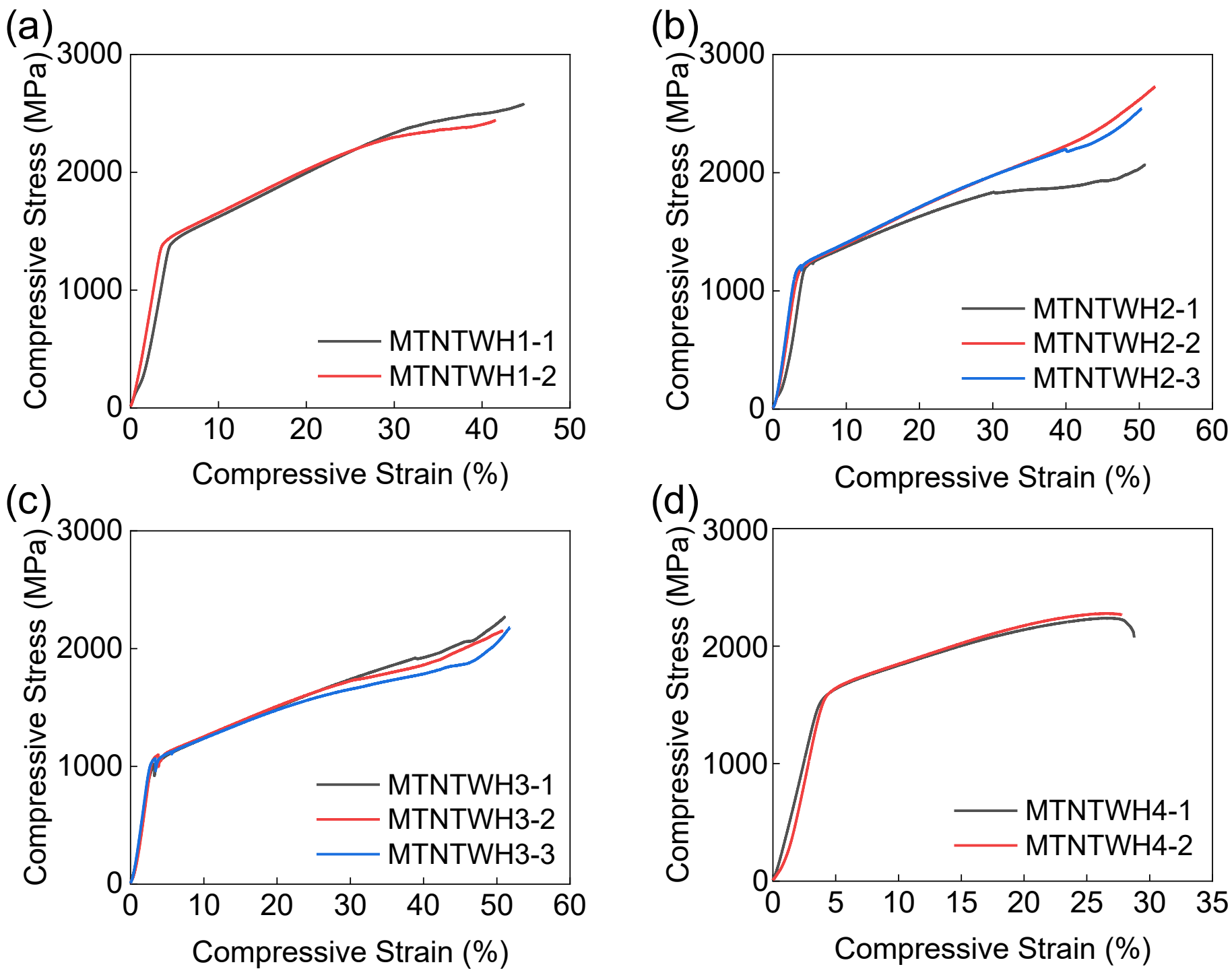

Supplementary Figure 8: Compressive stress–strain curves for the Mo-Ti-Nb-Ta-W-Hf (MTNTWH) high-entropy alloys. Subfigures (a)–(d) show the results for compositions MTNTWH 1 through 4, respectively. Source data are provided as a Source Data file.

Supplementary Table 3: The selected HEAs by the ML model for experimental validation.

Sample ID[a]	Ti	Nb	Ta	V	Zr	Hf
TNTVZH1	14.2±0.1	17.2±0.1	9.0 ± 0.2	13.0±0.0	34.8±0.0	11.8±0.0
TNTVZH2	18.4±0.0	17.0±0.1	26.1±0.1	6.3 ± 0.1	13.6±0.1	18.6±0.1
TNTVZH3	25.2±0.0	17.2±0.1	8.6 ± 0.1	24.0±0.1	19.2±0.1	5.7 ± 0.1
TNTVZH4	30.6±0.1	17.0±0.0	5.8 ± 0.1	6.3 ± 0.0	12.5±0.1	27.8±0.0
TNTVZH5	34.0±0.1	29.0±0.1	6.1 ± 0.1	5.1 ± 0.0	20.9±0.1	4.9 ± 0.0
Sample ID[a]	Mo	Ti	Nb	Ta	W	Hf
MTNTWH1	4.7 ± 0.0	25.9±0.5	16.8±0.2	16.1±0.4	6.9 ± 0.3	29.5±0.5
MTNTWH2	4.9 ± 0.1	32.5±0.5	29.5±0.2	10.7±0.3	8.9 ± 0.2	13.4±0.3
MTNTWH3	4.9 ± 0.1	34.2±0.1	29.8±0.1	15.5±0.1	6.3 ± 0.1	9.4 ± 0.1
MTNTWH4	6.7 ± 0.2	12.8±0.2	10.0±0.3	29.3±0.3	5.8 ± 0.2	35.5±0.3

[a] TNTVZH and MTNTWH denote two distinct HEA families, Ti-Nb-Ta-V-Zr-Hf and Mo-Ti-Nb-Ta-W-Hf, respectively.
