# Supplementary material for: Trillion-scale integrated framework for high-throughput materials databases and seamless sharing
Source: Nat Commun. 2026 Oct 7;17:11401. doi: 10.1038/s41467-026-78223-3 (PMC13736967; doi:10.1038/s41467-026-78223-3)
Supplement: Supplementary file 2 [file 41467_2026_78223_MOESM2_ESM.pdf]

The Description of Additional Supplementary Files

Supplementary Data 1: It contains the elemental parameters used for descriptor calculations, such as atomic radius, electronegativity, molar volume, monovacancy formation energy.
