# Supplementary material for: Trillion-scale integrated framework for high-throughput materials databases and seamless sharing
Source: Nat Commun. 2026 Oct 7;17:11401. doi: 10.1038/s41467-026-78223-3 (PMC13736967; doi:10.1038/s41467-026-78223-3)
Supplement: Supplementary file 3 [file 41467_2026_78223_MOESM4_ESM.pdf]

nature portfolio

Peer Review File

Trillion-Scale Integrated Framework for High-Throughput Materials Databases and Seamless Sharing

Corresponding Author: Dr 于 刘

This file contains all reviewer reports in order by version, followed by all author rebuttals in order by version.

Version 0:

Reviewer comments:

Reviewer #1

(Remarks to the Author)
The manuscript presents an innovative and technically robust framework that integrates stateless parallel computing with a lakehouse-based data management architecture to enable the construction and querying of trillion-scale materials databases. The proposed framework demonstrates impressive performance gains in storage efficiency, query speed, and computational scalability. However, several clarifications and methodological details are needed to strengthen the rigor and reproducibility of the study.

1. There are inconsistencies in figure citations. For instance, in the first paragraph of Section 2.3.1, the architecture schematic is referred to as ‘Figure 1’ but should be ‘Figure 2’ . A thorough check and correction of all figure references throughout the manuscript is recommended.
2. While Figure 2 effectively illustrates the high-level architecture of the stateless parallel computing system employing Redis message queues and Docker containers, the description of the operational workflow remains somewhat abstract for practical implementation. To significantly enhance the reproducibility and utility of the proposed framework, it is recommended to provide more detailed specifications of the core processes in the Supplementary Information.
3. Please specify the hardware setup (CPU types, memory per node, network bandwidth) used for the 500-core computation. This is critical for reproducibility and performance benchmarking.
4. The comparison with CSV and PostgreSQL is valuable, but additional comparisons with other modern frameworks (e.g., Spark, Dask, or dedicated HPC schedulers like Nextflow) would strengthen the performance claims. And providing more context on why Slurm was chosen as a baseline—was it configured optimally for I/O? A brief discussion of its limitations in this context would be helpful.
5. The ML model for plasticity prediction is based on a previous publication, but key details (feature selection, hyperparameters, validation metrics) should be summarized in the main text or SI. The GitHub repository is mentioned; please ensure it includes all code, metadata catalog samples, and a reproducible workflow example.
6. Only one HEA composition ($Ti_{30}Nb_{30}Ta_5V_5Zr_{25}Hf_5$) is experimentally validated. While this is a good proof-of-concept, the authors should discuss the generalizability of the AI predictions across other HEA families or suggest future validation studies.
7. The stateless design may not be optimal for iterative workflows (e.g., active learning) where state persistence is needed. Please comment on this.
8. Crucial details of the material descriptors are missing. These include the detailed computational methods of G, G0, K, Tb and etc. According to the introduction in the 2.3.1 section, the material descriptors only include 10 materials descriptors with specific calculating formulas and 46 elemental parameters. There is no crystal structure parameters can be found, so how to evaluated the modulus, formation energy and vacancy migration energy of HEAs?

(Remarks on code availability)

Reviewer #2

(Remarks to the Author)
Building on their previous work on machine learning applied to high-entropy alloys (HEAs), the authors of this manuscript

extend this work to classes containing 6 different chemical species, drawn from a total of 15 elements. This leads to an exponential increase of combinations, which motivated the authors to develop a computational framework suitable for such large amounts of computations, data transfer, storage, and retrieval.

Hence, the present manuscript focuses on information technology with HEAs serving as a test case. As a consequence, the paper overwhelms the reader with a plethora of computer science related terms such as DataForge, stateless computing, Redis queues, docker, container, lakehouse architecture, DuckDB, Parquet storage, DuckLake cloud sharing, and S3 protocol.

Despite the interesting application to HEAs, the present manuscript seems to be more appropriate for a journal focusing on software architectures and algorithms in data science rather than Nature Communications. Thus, I do not recommend publication in Nature Communications.

In any case, the authors should consider the following points in any resubmission.

1. Remove "DataForge" from the title.

2. Introduction: The first sentence is only partially true. The discovery of new materials is also, and perhaps even primarily, driven by a deeper understanding of the mechanisms leading to desired properties, aided by combined theoretical and computational efforts, as practiced, for example, by companies like QuesTek in the development of new steels.

3. Not all readers of this article are necessarily familiar with terms like DuckLake cloud sharing. Hence, they should be properly explained, which is partially done, or references should be provided.

4. The authors claim a 90% storage reduction and 180 times faster queries compared with traditional frameworks. This is an empty claim unless both the traditional framework and the performance test are exactly defined.

5. Stating that a full search across trillions of data in minutes (how many?) needs a specification of the processor, not only the memory usage.

6. The authors make the bold statement: "Existing solutions or technical frameworks are inadequate for the unique data structures and computational demands of materials science." Can the authors really defend this statement in view of the multitude of successful academic as well as commercial software solutions used in computational materials science?

7. Why do the authors discuss at length all 194 descriptors when at the end only five simple descriptors derived from the valence electron count and atomic radii are used as input features?

8. It is not clear how properties such as mixing enthalpy are computed. Do they involve DFT calculations of binary system? Are they taken from CALPHAD approaches?

9. Similarly, the computation of vacancy formation energies involving two elements should be explained.

10. The approximation of the melting temperature as a weighted average of melting temperatures of pure elements is rather crude, as it ignores, for example, eutectic behavior.

11. How would the approach scale for seven- or eight-component systems? Perhaps to this end, the authors could perform tests for five- and four-component systems and analyze the trends.

12. In the Summary and outlook section, the authors may want to point out other areas of materials research for their approach. Also, the limits of their approach should be discussed. One of the fundamental challenges in solid state science is the prediction of crystal structures. In the present case of HEAs, this question is avoided by considering random solid solution in a given lattice (BCC in this work). This is a very serious limitation. What happens if a system develops short-range order or precipitates second phase particles? Futhermore, in a microstructure, segregation to grain boundaries could have a significant effect on the macroscopic properties. Magnetism adds another major complication when atoms like Mn, Fe, Cr, Ni, and Co are present. This does not diminish the merits of the present machine-learning approach, but materials science is more than scaling to trillions of random configurations.

(Remarks on code availability)

Version 1:

Reviewer comments:

Reviewer #1

(Remarks to the Author)
The authors have adequately addressed all prior concerns. The manuscript is now well organized and suitable for

publication.

(Remarks on code availability)

Reviewer #2

(Remarks to the Author)
Overall, the authors have responded well to the questions raised in my report and they have made significant changes to the manuscript. Provided that the main emphasis of the paper is deemed appropriate for publication by the editor, I would recommend publication of the revised manuscript.

(Remarks on code availability)

Open Access This Peer Review File is licensed under a Creative Commons Attribution 4.0 International License, which permits use, sharing, adaptation, distribution and reproduction in any medium or format, as long as you give appropriate credit to the original author(s) and the source, provide a link to the Creative Commons license, and indicate if changes were made.
In cases where reviewers are anonymous, credit should be given to 'Anonymous Referee' and the source.
The images or other third party material in this Peer Review File are included in the article's Creative Commons license, unless indicated otherwise in a credit line to the material. If material is not included in the article's Creative Commons license and your intended use is not permitted by statutory regulation or exceeds the permitted use, you will need to obtain permission directly from the copyright holder.
To view a copy of this license, visit https://creativecommons.org/licenses/by/4.0/

Responses to reviewers:

Reviewer #1:

The manuscript presents an innovative and technically robust framework that integrates stateless parallel computing with a lakehouse-based data management architecture to enable the construction and querying of trillion-scale materials databases. The proposed framework demonstrates impressive performance gains in storage efficiency, query speed, and computational scalability. However, several clarifications and methodological details are needed to strengthen the rigor and reproducibility of the study.

Response: We sincerely thank the reviewer for the thoughtful and constructive evaluation of our work. We are grateful for the positive recognition of our framework's innovation, technical robustness, and the demonstrated performance gains in storage efficiency, query speed, and computational scalability. We fully agree with the reviewer’s call for additional clarifications and methodological details, and we appreciate these valuable suggestions for strengthening the rigor and reproducibility of our study. We provide point-by-point responses below, with all revisions highlighted in yellow and the section headings marked in green.

We believe these revisions have substantially improved the clarity and completeness of our work, and we hope the reviewer finds our responses satisfactory.

Question 1: There are inconsistencies in figure citations. For instance, in the first paragraph of Section 2.3.1, the architecture schematic is referred to as ‘Figure 1’ but should be ‘Figure 2’. A thorough check and correction of all figure references throughout the manuscript is recommended.

Response: We sincerely thank the reviewer for the careful review and for pointing out the inconsistencies in the figure citations. We apologize for these errors and have

conducted a thorough check of all figure citations throughout the manuscript. We identified four issues regarding figure citations and have corrected them as follows:

(1) We have corrected the figure reference in the first paragraph of Section 2.2.1. The text originally cited Fig. 1, which provides a schematic overview of the entire framework. We have revised it to correctly cite **Fig. 2a**, which specifically illustrates the stateless parallel computing architecture.

(2) We have added a missing reference to **Fig. 2b** in Section 2.2.2, which illustrates the lakehouse-based data management workflow.

(3)–(4) In Section 3.2, we have corrected two figure referencing errors. The text originally cited Fig. 3a and Fig. 3b, both of which depict the schema architecture of the large-scale database. We have revised these citations to correctly refer to **Fig. 4c** and **Fig. 4d**, respectively, which present the storage volume comparison across file formats and the query speed comparison across the four HEA cases.

Revised Section:

Revisions in the Manuscript

Page 5: line 170 (Section 2.2.1 Stateless parallel computation)

As shown in Fig. 2a, the architecture works by decoupling ...

Page 6: line 208 (Section 2.2.2 Lakehouse-based data management)

We proposed a data management chain based on a lakehouse architecture to enable efficient storage, utilization, and seamless sharing of scientific data between researchers (Fig. 2b).

Page 16: line 503 (Section 3.2 Advantages of the framework for the large-scale database)

As shown in Fig. 4c, we compared the storage volumes ...

Page 16: line 521 (Section 3.2 Advantages of the framework for the large-scale database)

As shown in Fig. 4d, while the remaining ...

Question 2: While Figure 2 effectively illustrates the high-level architecture of the stateless parallel computing system employing Redis message queues and Docker containers, the description of the operational workflow remains somewhat abstract for practical implementation. To significantly enhance the reproducibility and utility of the proposed framework, it is recommended to provide more detailed specifications of the core processes in the Supplementary Information.

Response: We sincerely thank the reviewer for this valuable suggestion. We agree that the high-level architecture in Fig. 2 alone is insufficient for practical implementation. In response, we have added a more detailed description of the operational workflow of the stateless parallel computing framework in the Supplementary Information. We have also released two code repositories to further facilitate adoption and implementation. The first, https://github.com/Wolido/idm-gridcore, provides a standalone implementation of the stateless parallel computing system that researchers can apply to their own computational tasks. The second, https://github.com/Wolido/agent-idm-gridcore, offers a natural-language interface powered by an AI agent skill, which automates the entire lifecycle of deployment, task submission, and execution monitoring. This significantly lowers the barrier for researchers without distributed-systems expertise. We briefly describe these below.

(1) Detailed operational workflow

We briefly summarize the core logic that users need to understand when applying the framework to their own computational tasks. First, the user must ensure that their computational task can be decomposed into independent and repetitive computational units. Based on this, the core execution logic is to package the subtask computations into Docker containers, and to push the required input data or information into Redis queue for scheduling management. In practice, the user writes their computation code (single-core program) as they normally would and then packages it into a Docker container. There is only one additional requirement. The code must include functions

for accessing Redis queue, pulling subtask data from the queue, and writing the computed results back to Redis.

Redis queue can be understood as a set of temporary storage slots on a server, accessible over the network. It must be deployed first. The user then writes a separate program to connect to the Redis list and push task data. Once the computation is complete, the same logic applies to result collection. The user writes another program to access Redis, retrieve results from the output queue, and store them in the database. The Redis deployment, task push, and result collection steps can be done either in Docker or directly on the server, as they are fast and run only a limited number of times. The subtask computations are recommended to be run in Docker to ensure fast deployment. One practical consideration is that a single Redis queue can become an I/O bottleneck under extremely high throughput. Using multiple parallel queues helps avoid the congestion that excessive data can cause. In this setup, worker nodes remain stateless and pull tasks from all available queues. Users also need to define their own data aggregation logic. The core Redis commands for deployment, access, push, and retrieval are available in the code repository https://github.com/Wolido/idm-gridcore.

(2) idm-gridcore: general-purpose stateless parallel computing framework (https://github.com/Wolido/idm-gridcore)

A procedural description alone may not be sufficient for researchers to readily adopt the framework. We therefore provide a standalone, general implementation of the framework at https://github.com/Wolido/idm-gridcore. The repository implements the conceptual workflow described above in a runnable system with three distinct layers. At the top layer, ComputeHub acts as the central scheduler that manages the task queues, coordinates compute nodes, and monitors the overall running status. At the middle layer, GridNode runs on each compute device and communicates with ComputeHub. It pulls Docker images, launches containers, and reports heartbeats to the scheduler. GridNode can be deployed on a wide range of devices, including servers, workstations, and even resource-constrained devices such as Raspberry Pi. At the bottom layer, Docker containers serve as the actual execution units. Each container is bound to a single CPU core, and the number of containers defaults to the number of CPU cores on each node,

with the option for users to adjust this setting. Each container pulls one task from the Redis input queue, performs the computation, and writes the result back to the output queue.

In order to help users get started quickly, the repository also provides detailed step-by-step guidance covering the entire deployment and execution workflow. This includes commands for starting the ComputeHub server, installing GridNode on compute nodes, registering computational tasks, launching compute nodes, finishing the current task, and stopping compute nodes, along with configuration and environment variable instructions. In addition, the repository contains a complete working example (https://github.com/Wolido/idm-gridcore/tree/master/test-workflow) that demonstrates the full framework implementation, which users can directly refer to and adapt for their own use.

(3) agent-idm-gridcore: AI-agent skill for automated deployment and use (https://github.com/Wolido/agent-idm-gridcore)

Although idm-gridcore is already simpler than traditional cluster schedulers, it still requires users to write Dockerfiles and queue-handling code. To make the framework accessible to researchers without distributed-systems experience, we developed agent-idm-gridcore (https://github.com/Wolido/agent-idm-gridcore), an AI-agent skill that wraps idm-gridcore. A user can describe a task in plain language, for example, "calculate the square roots of these 10,000 numbers using the cluster", and the agent will automatically check the local environment, deploy an idm-gridcore cluster if one is not already available, generate the consumer code and Dockerfile, register the task with ComputeHub, push the input data to Redis, monitor execution, and return the results. The skill includes code templates, environment-check scripts, and a "SKILL.md" guide for AI agents. Using the agent skill is optional. Users can still interact directly with idm-gridcore if they prefer.

In summary, the supplementary specifications and the two repositories together ensure that readers can understand, reproduce, and apply the framework in their own work. The corresponding revisions have been made in the manuscript and the Supplementary Information.

Revised Section:

(1) Revisions in the Manuscript

Page 6: line 197 to line 204 (Section 2.2.1 Stateless parallel computation)

A detailed description of the core operational workflow of the framework is provided in the Supplementary Information. In addition, a standalone implementation of this framework is available at https://github.com/Wolido/idm-gridcore, which includes user guidance and a complete working example. To further lower the adoption barrier, an AI-agent skill that automates deployment and task execution via natural-language instructions is provided at https://github.com/Wolido/agent-idm-gridcore. Detailed descriptions of both repositories are also provided in the Supplementary Information.

Page 22: line 687 to line 689 (Code Availability section)

The custom code developed for this study is available on GitHub at https://github.com/Wolido/hea_ducklake, https://github.com/Wolido/idm-gridcore, and https://github.com/Wolido/agent-idm-gridcore.

(2) **Revisions in the Supplementary Information**

Page 1 to page 2: line 25 to line 42 (Section S1.1 Core logic for applying the stateless parallel framework)

The framework is designed for computational tasks that can be partitioned into independent and repetitive computational units. The core execution logic is to package the subtask computations into Docker containers, and to push the required input data into Redis for scheduling management. Unlike routine execution without the framework, using our framework requires modifications to the computation code to include connecting to Redis, retrieving subtask data from the message queue, and writing the results to the message queue. Redis serves as a temporary queue on a server and must be deployed first. A separate program is then needed to push input data into Redis, and another program is required for result collection.

The Redis deployment, subtask push, and result collection steps are lightweight and can be run either in Docker or directly on the server. The subtask computations

themselves are recommended to be run in Docker to ensure fast deployment and consistent environments. A single Redis queue can become an I/O bottleneck under extremely high throughput. This can be mitigated by using multiple parallel queues. In this setup, worker nodes remain stateless and pull tasks from all available queues, but users need to define their own data aggregation logic to combine results from multiple queues. The core Redis commands for deployment, access, push, and retrieval are available in the code repository https://github.com/Wolido/idm-gridcore.

Page 2: line 44 to line 60 (Section S1.2 idm-gridcore: framework implementation and usage)

The repository https://github.com/Wolido/idm-gridcore provides a general-purpose implementation of the stateless parallel computing architecture shown in Fig. 2a. It consists of three coordinated components. ComputeHub is the central scheduler. It maintains the task registry and the list of online worker nodes, exposes a small web interface, and drives task switching. A task is registered with a JSON payload that specifies the Docker image and the input and output Redis queues. The administrator advances the workflow by requesting a task transition through the appropriate endpoint. GridNode runs on each worker machine. It registers itself with ComputeHub on startup, sends periodic heartbeats, and launches one Docker container per central processing unit core. Each container receives the Redis connection parameters and queue names as environment variables, retrieves one task at a time from the input queue, performs the computation, and writes the result to the output queue. The repository includes the complete source code for ComputeHub and GridNode, a Docker Compose template for Redis, cross-platform build scripts, example configuration files, and a minimal working example (https://github.com/Wolido/idm-gridcore/tree/master/test-workflow) that computes the squares of one million numbers in parallel.

Page 2 to Page 3: line 63 to line 71 (Section S1.3 agent-idm-gridcore: AI-agent skill for automated deployment and use)

The repository https://github.com/Wolido/agent-idm-gridcore provides an AI-agent skill that wraps idm-gridcore. It allows users to deploy and use the distributed computing framework through natural-language dialogue rather than manual

configuration. Given a description of a computational task, the agent checks the environment, deploys a cluster if needed, generates the consumer code and Dockerfile, registers the task with ComputeHub, pushes data to Redis, monitors execution, and returns the results. The repository also includes installation instructions and usage guidelines for the skill. Direct use of idm-gridcore remains possible for users who prefer manual control.

Question 3: Please specify the hardware setup (CPU types, memory per node, network bandwidth) used for the 500-core computation. This is critical for reproducibility and performance benchmarking.

Response: We thank the reviewer for pointing out this important omission. We agree that detailed hardware specifications are essential for reproducibility and meaningful performance benchmarking. The 500-core computation was performed on a cluster of 10 nodes, each equipped with Intel Xeon Gold 6330 CPUs (56 cores per node, 2.00 GHz) and 512 GB of RAM, with 50 cores utilized per node. The nodes were interconnected via 10GbE optical modules, providing an internal network bandwidth of over 9000 Mbps. These hardware details have been added to the revised manuscript.

Revised Section:

Revisions in the Manuscript

Page 11: line 350 to line 354 (Section 2.3.2 Implementation of the integrated framework)

This process was supported by 4 upstream and 4 downstream Redis queues, and utilized 500 CPU cores distributed across 10 compute nodes (each core running in a separate Docker container). The compute cluster comprised Intel Xeon Gold 6330 processors (56 physical cores per socket at 2.00 GHz; 50 cores utilized per node), 512 GB of RAM per node, and a 10 GbE interconnect providing over 9000 Mbps of internal bandwidth.

Question 4: The comparison with CSV and PostgreSQL is valuable, but additional comparisons with other modern frameworks (e.g., Spark, Dask, or dedicated HPC schedulers like Nextflow) would strengthen the performance claims. And

providing more context on why Slurm was chosen as a baseline—was it configured optimally for I/O? A brief discussion of its limitations in this context would be helpful.

Response: We thank the reviewer for this important suggestion and for the positive recognition of our comparison with CSV and PostgreSQL. We also acknowledge the value of extending this comparison to additional modern distributed frameworks and the need to clarify our choice of Slurm as a baseline. To address these points, we have benchmarked our framework against several representative platforms, including Spark and Dask as execution-layer frameworks, Nextflow as a workflow orchestration system, and Slurm as a resource scheduler. Slurm in particular was chosen as a baseline due to its widespread adoption in academic and research computing environments. We address each point in turn below.

All the tests were performed on the same hardware, using 560 CPU cores across 10 nodes (Intel Xeon Gold 6330, 2.00 GHz), with 512 GB RAM per node and a 10 GbE interconnect delivering over 9000 Mbps. The full 56 cores per node were used rather than the 50 cores per node employed in the original manuscript. This adjustment was made to avoid CPU swapping that could arise when using Dask, which would introduce additional time overhead and compromise measurement accuracy. We computed the materials descriptors for over 50 billion compositions spanning 5,005 six-element HEA families by Spark, Dask and our framework under a new configuration. For Slurm, we limited the test to a single family of 10,762,647 HEA compositions. This is because Slurm relies primarily on disk-based I/O rather than in-memory processing. The high-frequency random writes generated by full-scale computation would cause excessive wear on storage media, especially SSDs, and significantly reduce their service life. We therefore chose a single family as a representative baseline.

The performance comparison is summarized in **Fig. R1a**, which presents the runtime and memory usage. Dask failed to complete the workload. Each worker showed quasi-linear memory growth at a rate of approximately 52.2 GB per hour and reached the preset memory limit of 480 GB after about 9.2 hours, triggering out-of-memory

errors. Slurm executed the computation in approximately 29 seconds, with a peak memory usage of ~ 1.7 GB. Extrapolating to all 5,005 families would give an estimated runtime of approximately 40.3 hours. Notably, while Slurm maintained a low runtime memory footprint, it incurred massive storage I/O overhead. Spark completed the computation in approximately 21.3 hours with a peak memory usage of 337.0 GB. This result was achieved by moving composition generation from the driver to the executors, so that the driver only manages element combinations rather than billions of individual compositions. This aligns with Spark's coarse-grained design and represents a fair performance baseline. In contrast, fine-grained task management, where the driver tracks individual HEA compositions, would generate billions of tasks and drive memory demand far beyond the 512 GB node limit, leading to rapid failure. This bottleneck is inherent to Spark's centralized driver model and would be further amplified in higher-dimensional systems such as seven-element HEAs. For Slurm, we used the default configuration without I/O tuning, which reflects typical HPC usage. Our framework's I/O approach could also be leveraged to optimize Slurm in future studies.

For our framework, in the original manuscript, each Docker worker pulled one HEA composition at a time from Redis queue, computed the descriptors, and pushed the result back. It consumed only 30.1 MB of memory per docker worker and around 1.7 GB (30.1 MB*56) in total across a CPU node, but required approximately one week to complete the full task. This configuration minimizes memory usage and makes the architecture viable on very small edge devices, although the Redis overhead becomes substantial when processing billions of compositions. To achieve higher throughput and enable a direct performance comparison with Spark, we used an alternative configuration in which each worker pulled 200,000 HEAs compositions in a single batch. This reduced the Redis overhead and completed the computation in approximately 26.7 hours, with peak memory usage of 146.9 GB across the cluster. To provide a quantitative comparison of overall resource efficiency, we used the product of runtime and peak memory usage as a combined metric. Dask failed to complete the computation due to memory exhaustion, and Slurm was excluded from this comparison

because its performance is dominated by disk I/O rather than memory usage. For Spark, the time-memory product was approximately 7.18 × 10^3 GB·h (21.3 h × 337.0 GB), while our framework achieved approximately 3.97 × 10^3 GB·h (26.7 h × 146.9 GB), representing a 45% reduction. In summary, our framework generally outperformed Slurm, Spark, and Dask in both computational speed and memory efficiency.

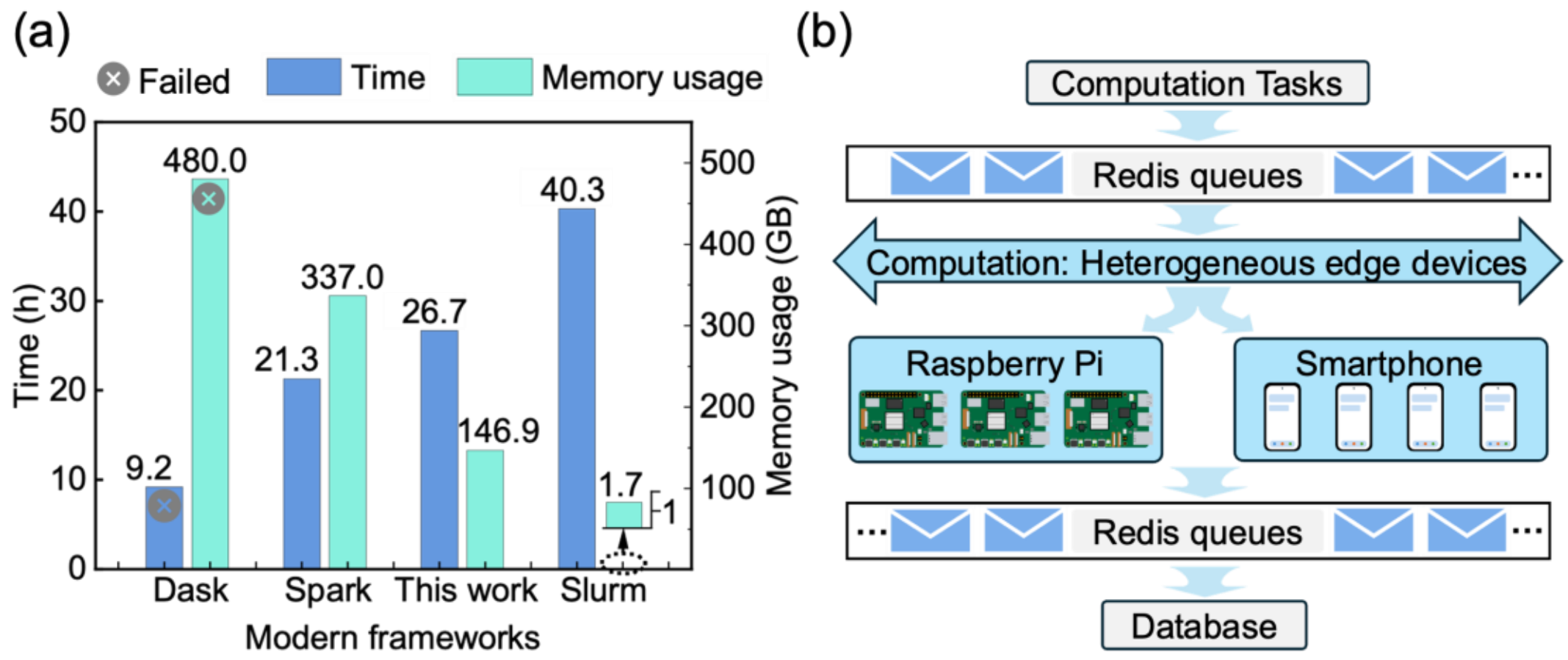

Fig. R1 (a) Performance comparison of Spark, Dask, Slurm, and our framework for the HEA descriptor computation. (b) Schematic illustration of the framework architecture with heterogeneous edge devices as compute nodes.

We further analyze the differences between these frameworks and our framework. Spark relies on a dedicated runtime environment with a driver, executors, and a cluster manager. This runtime itself consumes baseline memory for scheduling, serialization, and shuffle services, an overhead that is inherent to Spark's architecture and independent of task granularity. Dask workers are long-running processes that persist after each task. Memory pages allocated during execution are typically not returned to the operating system even after garbage collection, causing memory usage to accumulate gradually over time (**Fig. R2**). Slurm, however, shows a different pattern. Its memory footprint is low, approximately 1.7 GB in our test, because Slurm primarily relies on disk-based I/O interactions and writes task outputs directly to disk. This approach generates numerous fragmented files and creates difficulties in data access and management. In our single-family test, the tasks were evenly distributed across 560 cores, producing 560 separate output files. Extrapolating to the full set of 5,005 HEA

families would generate approximately 2.8 million files. The burst writing of such a huge number of small files would introduce severe system-level bottlenecks. The metadata cache would face intense pressure, as the frequent creation and access of large numbers of files would rapidly exhaust the inode and dentry caches of the storage system, leading to severe metadata operation latency. In a full-scale run, even listing the contents of a directory would become unresponsive. The post-processing stage would require aggregating these massive numbers of files, consuming substantial CPU and I/O bandwidth and becoming a critical bottleneck for overall data throughput.

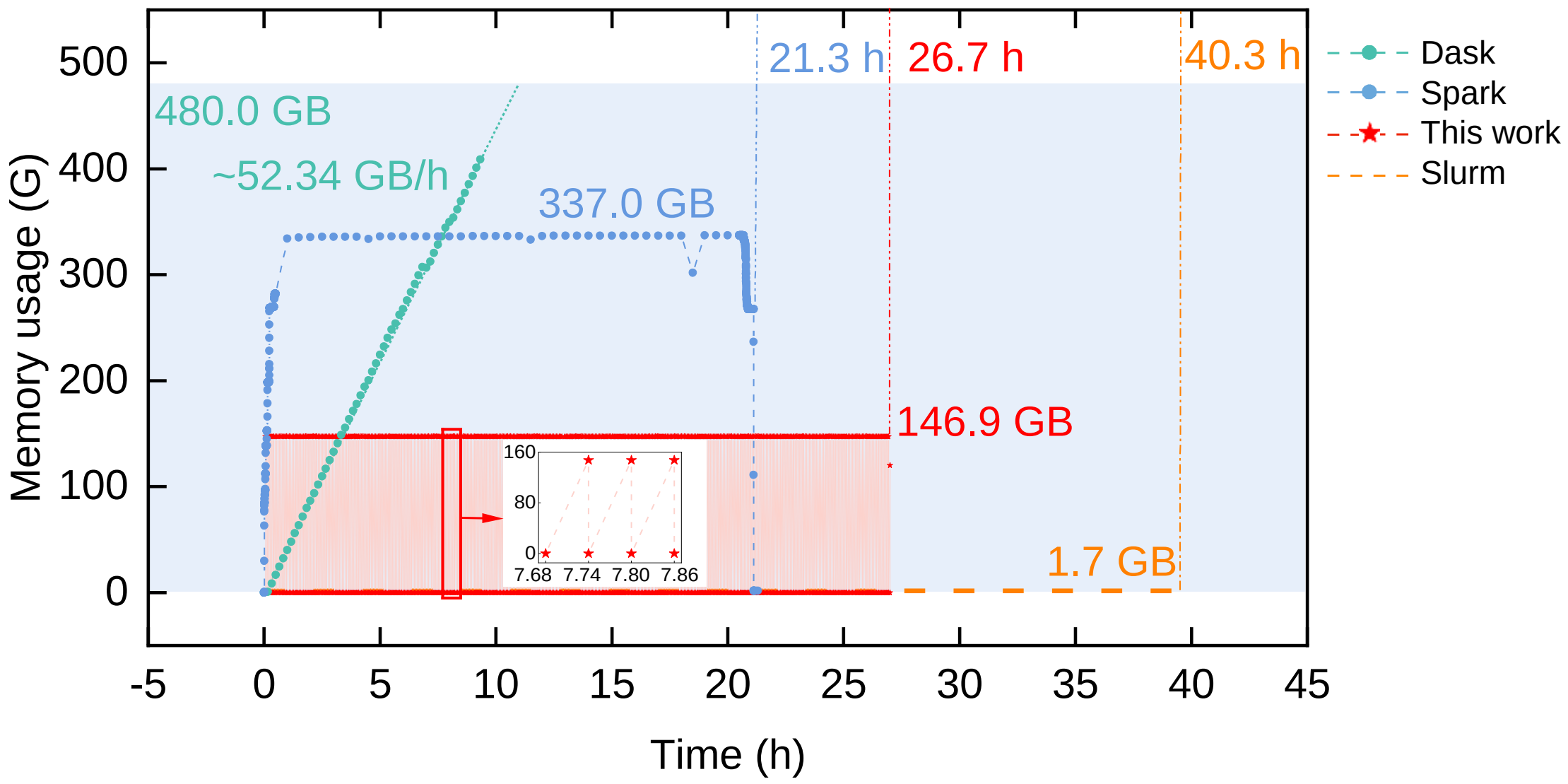

Fig. R2 Memory usage per compute node over runtime for Spark, Dask, Slurm, and our framework during the HEA descriptor computation. The embedded zoomed-in panel displays our framework's memory behavior across three Docker worker runtime cycles.

In contrast, our framework offers a fundamentally different operational model. It does not require a dedicated runtime to remain active throughout the computation. In addition, the stateless workers only consume memory during active task execution as demonstrated in **Fig. R2**. This allows for extremely low memory footprints in the single-task configuration. Moreover, task distribution relies on Redis as a lightweight queue, eliminating the need for high-speed internal network interconnects among worker nodes. As a result, the framework is well suited for heterogeneous and resource-constrained edge devices. To verify this flexibility, we successfully tested two

heterogeneous edge devices as compute nodes, as illustrated in **Fig. R1b,** which are a Raspberry Pi Zero 2W powered by an ARM Cortex-A53 processor with 512 MB RAM and a smartphone equipped with an ARM Cortex-A520 processor with 16 GB RAM. They successfully connected to the same Redis queue simultaneously, pulled subtask data from it, and completed the calculations. For most edge devices, we recommend running the computation inside Docker containers to ensure fast deployment and environment consistency. For devices that cannot run Docker, such as the smartphone used in this test, the computation code can be executed directly on the device without containerization. These results confirm that the framework can effectively utilize a wide range of computing resources, from dedicated cluster nodes to heterogeneous edge devices.

Nextflow is a workflow orchestration system designed for multi-step pipelines with complex interdependencies. It manages task dependencies and submission logic rather than executing computations directly. This makes Nextflow a complementary tool for orchestrating heterogeneous workflows rather than a direct competitor for high-throughput task-parallel computation. We therefore did not include Nextflow in the performance benchmark, as it addresses a different class of problems. Moreover, our framework does not require such complex orchestration and can be deployed and executed efficiently with minimal operational overhead.

We have expanded the performance discussion in the manuscript with detailed benchmark results and analysis. In addition, more details of these frameworks have also been supplied in the Supplementary Information in order to maintain readability.

Revised Section:

(1) Revisions in the Manuscript

Page 2: line 34 to line 38 (Abstract)

Our framework cuts runtime–memory cost by 45% versus distributed frameworks (Spark, Dask, Slurm), reduces storage by 90% for the full HEA database versus row-based formats (CSV, JSON), and accelerates subset queries by up to 180 times versus CSV and PostgreSQL.

Page 12 to Page 13: line 407 to line 409 (Section 3.2 Advantages of the framework for the large-scale database)

Using this framework, high-throughput calculations of more than 10 trillion materials descriptors were completed in one week.

Page 13 to Page 16: line 410 to line 491 (Section 3.2 Advantages of the framework for the large-scale database)

To further quantify these advantages, we evaluated the performance of our framework against several representative distributed platforms, including Spark and Dask as execution-layer frameworks, and Slurm as a resource scheduler. Slurm in particular was selected as a baseline, given its widespread adoption in academic and research computing environments. All tests were performed using 10 nodes with 56 cores per node, rather than the 50 cores per node used previously. This adjustment avoided CPU swapping that would otherwise arise when running Dask on partially allocated cores, introducing additional time overhead and compromising measurement accuracy. We executed Spark, Dask, and our framework to compute materials descriptors for over 50 billion compositions across 5,005 six-element HEA families. For Slurm, we restricted the test to a single family of 10,762,647 compositions to avoid excessive wear on storage media, as Slurm relies primarily on disk-based I/O rather than in-memory processing.

As shown in Fig. 4a, Dask failed to accomplish the task. Memory usage on each worker grew at a quasi-linear rate of about 52.2 GB per hour, and the configured limit of 480 GB was reached after roughly 9.2 hours, resulting in out-of-memory errors (Fig. S7). Slurm finished a single HEA family of 10.7 million compositions in about 29 seconds, with a peak memory footprint of approximately 1.7 GB. Extrapolated to all 5,005 families, the estimated runtime would be around 40.3 hours. Spark completed the computation in approximately 21.3 hours with a peak memory usage of 337.0 GB. For our framework, when each Docker worker pulled one HEA composition at a time, the memory consumption was only 30.1 MB per worker, totaling approximately 1.7 GB across a CPU node (56 cores). While this setup minimizes memory usage and is viable

on resource-constrained edge devices, the Redis communication overhead becomes significant at the scale of billions of compositions. To increase throughput and enable a direct comparison with Spark, we adopted an alternative configuration in which each worker pulled 200,000 HEA compositions per batch. This approach reduced the Redis overhead and completed the computation in about 26.7 hours, with a peak cluster-wide memory usage of 146.9 GB. These two configurations illustrate the tunable balance between memory footprint and computational speed that our framework offers. It should be noted that the result of Spark is optimized by shifting composition generation from the driver to the executors, such that the driver only handles element combinations instead of billions of individual compositions. This is consistent with Spark's coarse-grained design and offers a fair performance baseline. By contrast, if the driver were to manage fine-grained tasks involving individual HEA compositions, the task count would climb to billions and push memory usage well beyond the 512 GB node limit, eventually causing job failure. We used the default Slurm configuration without I/O tuning, which is the typical setup in most research. Overall, our framework outperformed Slurm and Dask in efficiency and achieved a runtime comparable to that of Spark while using considerably less memory. To provide a quantitative comparison of overall resource efficiency, we used the product of runtime and peak memory usage as a combined metric. Dask failed to complete the computation due to memory exhaustion, and Slurm was excluded from this metric because its performance is dominated by disk I/O rather than memory usage. The time-memory product for Spark was approximately 7.18×10^3 GB·h, while our framework achieved approximately 3.97×10^3 GB·h, representing a 45% reduction.

The architecture underlying these performance gains is as follows. Spark requires a dedicated runtime environment that consumes baseline memory for scheduling, serialization, and shuffle services regardless of task granularity. Dask workers are long-running processes that retain memory pages even after garbage collection, causing memory to accumulate over time. Slurm, while memory-efficient, relies primarily on disk-based I/O and writes task outputs directly to disk, generating fragmented files that complicate data aggregation across multiple nodes. In our single-family test, tasks were

evenly distributed across 560 cores, producing 560 output files. Extrapolating to the full set of 5,005 HEA families would produce roughly 2.8 million files, placing intense pressure on the metadata cache and exhausting the filesystem caches of the storage system. The post-processing stage would then require aggregating this massive number of files, which would consume substantial CPU and I/O bandwidth and ultimately become a critical bottleneck for overall throughput. In this work, we used Redis queues for task distribution and result collection, with task executions completed in Docker containers. The upstream and downstream nodes of the containers are both Redis queues, providing an isolated and stateless execution environment. Workers consume memory only during active task execution (Fig. S7), and task distribution through Redis eliminates the need for high-speed internal network interconnects among worker nodes. The stateless design of our framework inherently supports elastic scaling of compute nodes and cross-cluster distributed computing, allowing resources to be aggregated across physical boundaries without I/O overhead or memory accumulation. The two test configurations demonstrate the flexibility of our framework in balancing memory usage and computational time. Therefore, it also accommodates heterogeneous and resource-constrained environments, including edge devices. We tested two edge devices as compute nodes, a Raspberry Pi Zero 2W powered by an ARM Cortex-A53 processor with 512 MB RAM and a smartphone equipped with an ARM Cortex-A520 processor with 16 GB RAM, as shown in Fig. 4b. Both devices successfully connected to the same Redis queue, pulled subtask data, and completed the descriptor calculations. For most edge devices, we recommend running computations inside Docker containers to ensure fast deployment and environment consistency. For devices that cannot run Docker, such as the smartphone used in this test, the computation code can be executed directly. Our framework can also be integrated into existing workflow orchestration systems such as Nextflow, though it remains fully functional and deployable independently without such orchestration. These results confirm that the framework can effectively utilize a wide range of computing resources, from dedicated cluster nodes to edge devices. More details are provided in the Supplementary Information.

Page 21: line 646 to line 648 (Summary and outlook)

Benchmarking against modern distributed frameworks, including Spark, Dask, and Slurm, shows that our framework achieves a 45% reduction in the combined time-memory metric.

Page 15: Revised Fig. 4

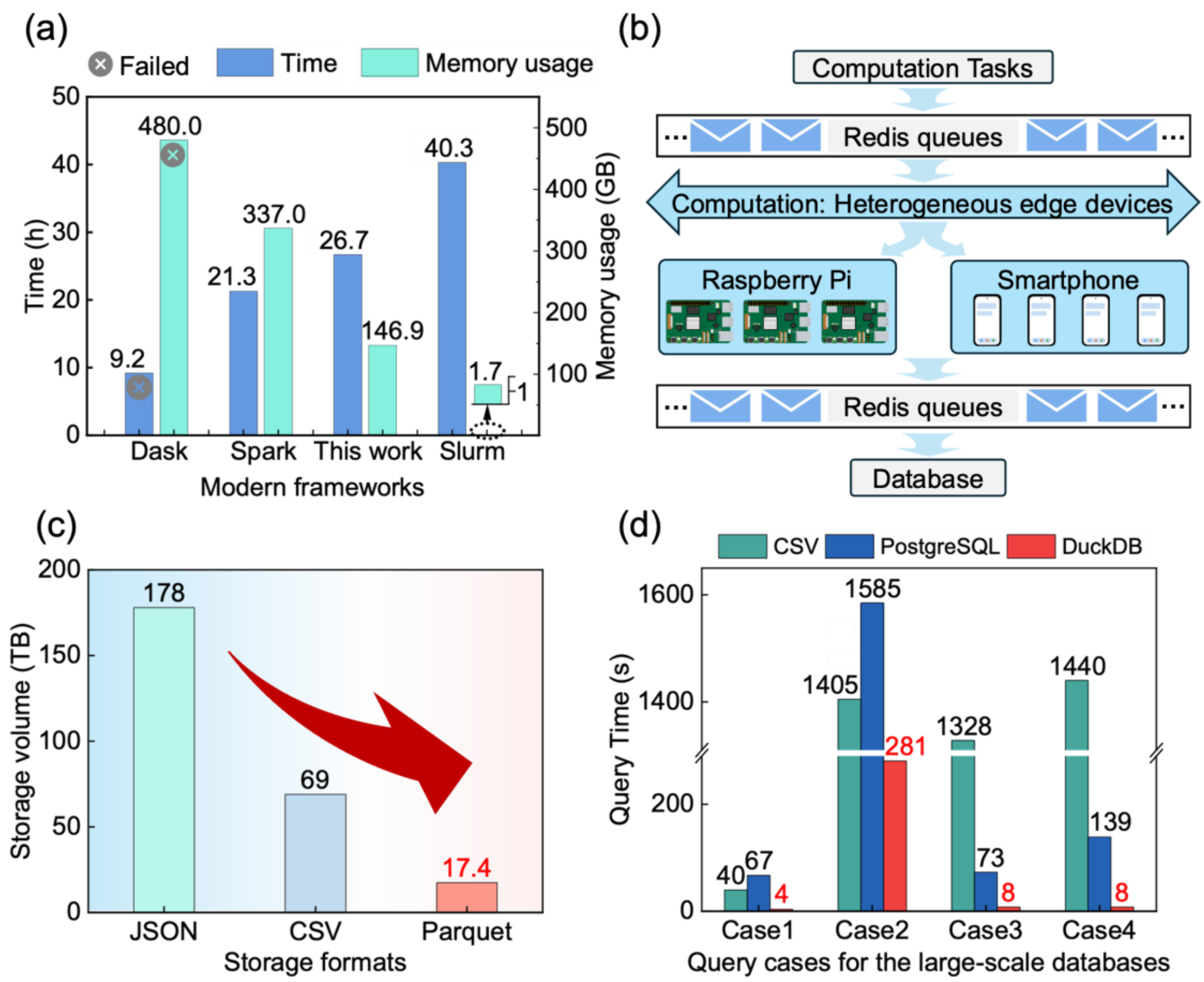

Fig. 4 Performance benchmark of distributed frameworks and data management architectures for large-scale materials data. (a) Runtime and per-node peak memory usage for Spark, Dask, Slurm, and our framework on the HEA descriptor computation. (b) Schematic illustration of the framework architecture with heterogeneous edge devices as compute nodes. (c) Storage volume comparison between columnar Parquet, row-based formats (CSV and JSON), and PostgreSQL. (d) Query speeds for four HEA cases using CSV, PostgreSQL, and DuckDB. The four query cases are defined in Table 1.

Page 22: line 653 to line 655 (Summary and outlook)

We have also validated the framework on heterogeneous edge devices, confirming its adaptability to resource-constrained environments beyond conventional computing clusters.

(2) Revisions in the Supplementary Information

Page 13: Additional Fig. S7

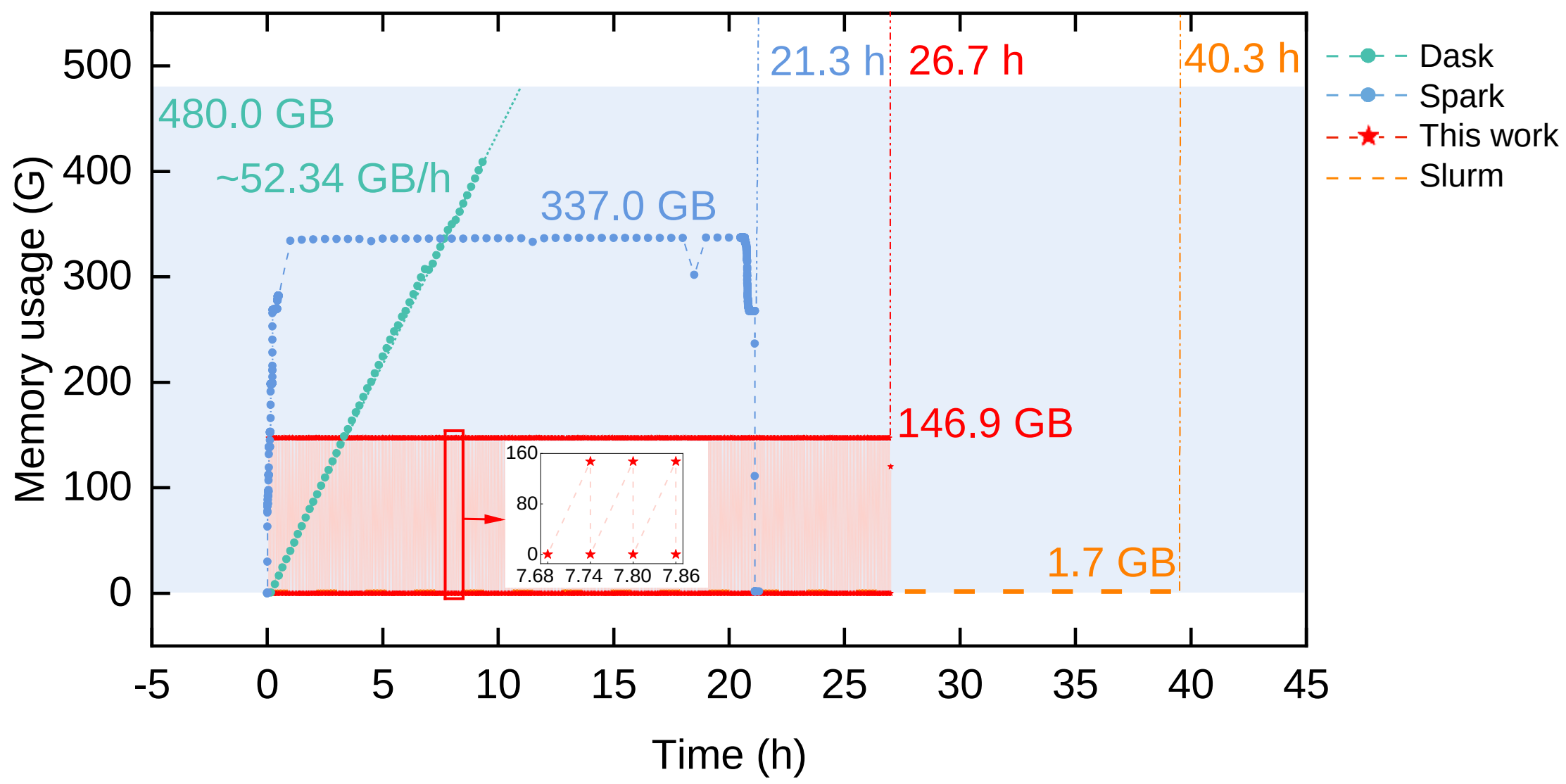

Fig. S7 Memory usage per compute node over runtime for Spark, Dask, Slurm, and our framework during the HEA descriptor computation. The embedded zoomed-in panel displays our framework's memory behavior across three Docker worker runtime cycles.

Page 7: line 125 to line 131 (Section S7.1 Spark configuration)

The Spark test used Apache Spark 3.5.8 with a mapPartitions architecture. Composition generation and descriptor computation were performed locally within each executor. The output was written as Parquet files compressed with Snappy, totaling 23 TB. Spark runs on the Java Virtual Machine, and each executor is a JVM process that manages task execution and data storage. The JVM heap stores task scheduling information, partition metadata, and serialization buffers. Each executor processed one full six-element family, corresponding to approximately 10.7 million compositions.

Page 7: line 133 to line 134 (Section S7.2 Dask configuration)

Dask 2025.1.0 was applied to the performance test. The workload was partitioned into 101 groups of 50 families, each containing approximately 535 million compositions.

Page 7: line 136 to line 138 (Section S7.3 The computations by Slurm)

Our single-family test was executed on Slurm 19.05.3-2, with all composition calculations evenly distributed across 560 CPU cores, generating 560 separate output files.

Question 5: The ML model for plasticity prediction is based on a previous publication, but key details (feature selection, hyperparameters, validation metrics) should be summarized in the main text or SI. The GitHub repository is mentioned; please ensure it includes all code, metadata catalog samples, and a reproducible workflow example.

Response: We sincerely thank the reviewer for these constructive suggestions. We fully agree that summarizing the ML model details and ensuring the completeness of the open-source repositories are essential for reproducibility. We have addressed both points as follows:

(1) ML modelling details

In the original manuscript, we have provided the core information of the ML model, including the dataset (92 compressive fracture plastic strain ε_p values of as-cast body-center-cubic (BCC) HEAs), the applied algorithms (GradientBoostingClassifier), input features (the mean and mismatch values of valence electron concentration, the mean atomic radius, the mismatch of covalent radius binary pairs, and the atomic size mismatch), output target (three categories: low plasticity (ε_p < 10%), moderate plasticity (10% ≤ ε_p < 40%), and high plasticity (ε_p ≥ 40%), encoded as labels -1, 0, and 1, respectively), and some other processing details. We have also described the optimization process in the original Supplementary Information (Section S3), covering feature engineering and algorithms selection.

In addition, we have included more detailed information in the revised manuscript and the Supplementary Information, covering the final performance metrics and model deployment. Specifically, the GB model achieves training and leave-one-out cross-validation accuracy of 100% and 85.9%, respectively, which are now presented in Fig. S2. The GB model was exported to ONNX format to enable efficient batch

inference across the distributed computing cluster and was provided in in the GitHub repository (https://github.com/Wolido/hea_ducklake). The normalization parameters of input features and the usage example of the ONNX models were also included in this repository.

(2) hea_ducklake: HEA case study (https://github.com/Wolido/hea_ducklake)

The original repository contains the source code for constructing the senary HEA database, along with the metadata catalog and usage examples. In addition, we have added a reproducible workflow example available at https://github.com/Wolido/hea_ducklake/tree/main/examples/minimal_workflow. This workflow demonstrates the complete pipeline on a small-scale task (100 randomly sampled HEA compositions), covering the process from Redis queue setup and Docker container orchestration to distributed execution of descriptor-calculation workloads and dataset storage and management. The workflow is fully documented in the "README.md".

Revised Section:

(1) Revisions in the Manuscript

Page 11: line 328 to line 329 (Section 2.3.1 Database contents and structure)

The GB model achieves training and leave-one-out cross-validation accuracy of 100% and 85.9%, respectively (Fig. S2).

Page 11: line 337 to line 340 (Section 2.3.1 Database contents and structure)

The GB model is exported to ONNX format to enable efficient batch inference across the distributed computing cluster. The ONNX model, along with the normalization parameters and usage examples, is provided in the GitHub repository (https://github.com/Wolido/hea_ducklake).

Page 11: line 365 to line 367 (Section 2.3.2 Implementation of the integrated frame)

The complete implementation of the HEA database construction pipeline is available at Supplementary Information and https://github.com/Wolido/hea_ducklake/tree/main/examples/minimal_workflow.

(2) Revisions in the Supplementary Information

Page 3: line 73 to line 80 (Section S2 Data and executable code for the HEA case study)

The repository (https://github.com/Wolido/hea_ducklake) contains the source code for constructing the senary HEA database, the metadata catalog, and usage examples. A reproducible workflow example is also provided at https://github.com/Wolido/hea_ducklake/tree/main/examples/minimal_workflow. This workflow executes a small-scale task on 100 randomly sampled HEA compositions, covering the entire process from Redis queue setup and Docker container orchestration to distributed descriptor-calculation workloads and dataset storage and management. The workflow is documented in the "README.md".

Page 5: Additional Fig. S2.

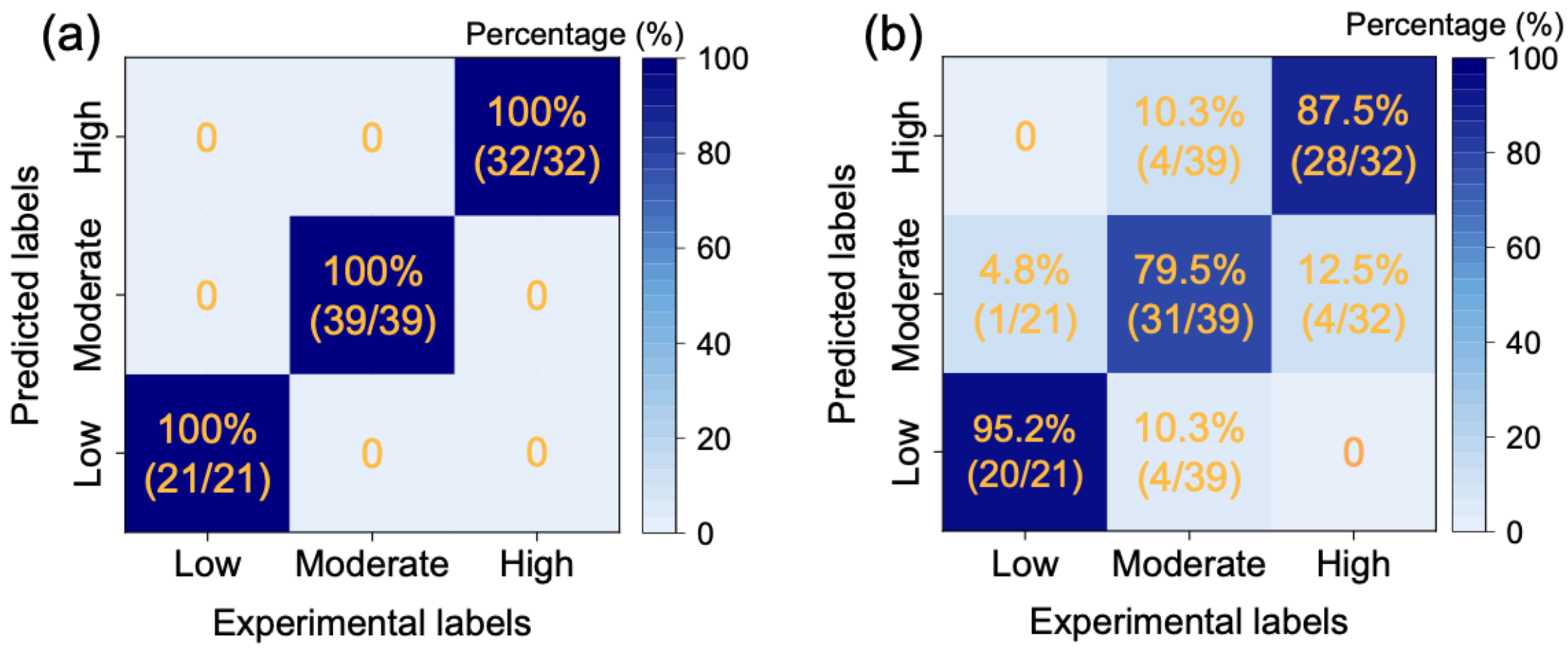

Fig. S2 Prediction performance of the retrained GB model. (a) Training confusion matrix; (b) LOOCV confusion matrix.

Page 3: line 105 to line 107 (Section S2 The machine learning model for predicting the plasticity of high entropy alloys)

The GB model used in this work were retrained using the whole 92 data with the training and leave-one-out cross-validation (LOOCV) accuracy of 100% and 85.9%, respectively (Fig. S2).

Question 6: Only one HEA composition ($Ti_{30}Nb_{30}Ta_5V_5Zr_{25}Hf_5$) is experimentally validated. While this is a good proof-of-concept, the authors should discuss the generalizability of the AI predictions across other HEA families or suggest future validation studies.

Response: We thank the reviewer for raising the important points. We agree that validation on a single composition, while valuable as a proof of concept, is insufficient to demonstrate broad applicability. Therefore, we have substantially expanded our experimental validation by adding eight new compositions across two distinct HEA families, which are the original Ti-Nb-Ta-V-Zr-Hf family and a refractory Mo-Ti-Nb-Ta-W-Hf family. The detailed experimental results are presented below.

First, as shown in the original manuscript, within the Ti-Nb-Ta-V-Zr-Hf family, the AI model predicted that approximately 64% of compositions exhibit high plasticity (compressive plastic strain ε_p>40%). We selected four additional HEAs (listed in Table R1) spanning a wide range of component ratios for experimental validation. As shown in Fig. R3a and Fig. R3b, all four HEAs exhibited a single BCC phase and compressive plastic strains exceeding 40%, consistent with the predictions. These results confirm that the AI model reliably identifies high-plasticity compositions within this family beyond the originally tested $Ti_{30}Nb_{30}Ta_5V5Zr_{25}Hf_5$.

Table R1. The predicted and experimental ε_p of the selected HEAs.

Sample ID [a)]	Composition (at. %)	Prediction [b)]	Experimental ε_p (%)
TNTVZH1	$Ti_{14}Nb_{18}Ta_8V_{13}Zr_{35}Hf_{12}$	High	>40
TNTVZH2	$Ti_{18}Nb_{18}Ta_{26}V_6Zr_{13}Hf_{19}$	High	>40
TNTVZH3	$Ti_{25}Nb_{18}Ta_8V_{24}Zr_{19}Hf_6$	High	>40
TNTVZH4	$Ti_{30}Nb_{18}Ta_5V_6Zr_{12}Hf_{29}$	High	>40
TNTVZH5	$Ti_{34}Nb_{30}Ta_5V_5Zr_{21}Hf_5$	High	>40
MTNTWH1	$Mo_5Ti_{25}Nb_{18}Ta_{16}W_6Hf_{30}$	High	36.5±0.5

MTNTWH2	$Mo_5Ti_{34}Nb_{30}Ta_9W_7Hf_{15}$	High	>40
MTNTWH3	$Mo_5Ti_{35}Nb_{31}Ta_{14}W_5Hf_{10}$	High	>40
MTNTWH4	$Mo_7Ti_{12}Nb_{11}Ta_{30}W_5Hf_{35}$	High	23.5±0.5

[a] TNTVZH and MTNTWH denote two distinct HEA familiess, Ti-Nb-Ta-V-Zr-Hf and Mo-Ti-Nb-Ta-W-Hf, respectively; [b] Predicted plasticity regime based on the GB classifier: High (ε_p > 40%).

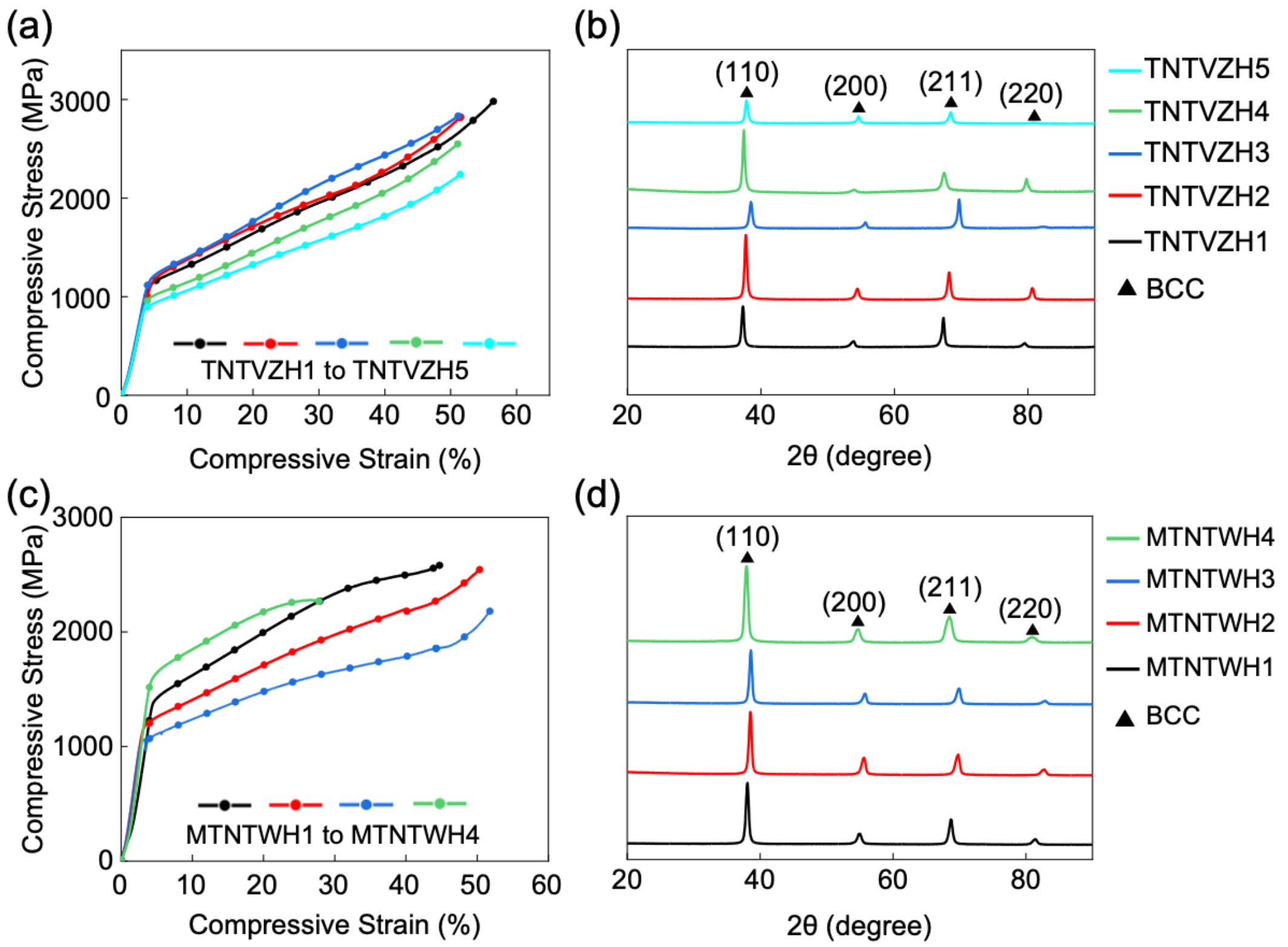

Fig. R3 Experimental validation of the selected HEAs. (a) and (b) Compressive stress-strain curves and XRD patterns for the Ti-Nb-Ta-V-Zr-Hf HEAs (labeled as TNTVZH HEAs). (c) and (d) Compressive stress-strain curves and XRD patternsfor the Mo-Ti-Nb-Ta-W-Hf HEAs (labeled as MTNTWH HEAs). The replicate test results for compressive stress-strain curves are provided in Figs. S8–S9.

Second, to evaluate cross-family transferability of the AI-design method, we also selected the Mo-Ti-Nb-Ta-W-Hf family for experimental validation. The base

Mo-Nb-Ta-W quaternary is a prototypical refractory high-entropy alloy (RHEA) that exhibits remarkable high-temperature performance. For example, the equiatomic $Mo_{25}Nb_{25}Ta_{25}W_{25}$ RHEA achieves yield strengths exceeding 400 MPa at 1600 °C. However, it exhibits severe room-temperature brittleness, with compressive ε_p typically below 5%. Our high-throughput screening predicted that introducing Ti and Hf into this base family could improve the compressive plasticity to above 40%. According to the established large-scale database, this family shows a predicted high-plasticity proportion of approximately 0.09%. As mentioned in the manuscript, the GB model is built to predict the compressive plasticity of HEAs showing a single BCC solid-solution phase. The phase criteria ($\Omega \geq 1.1$, $\delta r \leq 6.6\%$, and $VEC < 6.87$) are used to classify single BCC solid-solution phase. As shown in Table R2, the calculated parameters for the Mo-Ti-Nb-Ta-W-Hf family satisfy these criteria, indicating that this family also predominantly exhibits BCC phase. Four compositions from this family (listed in Table R1) were selected for experimental validation. As shown in Fig. R3c, two compositions exhibited high plasticity, with compressive ε_p exceeding 40%, while two showed moderate plasticity (36.5% and 23.5%). XRD results confirmed a single BCC phase in all cases (Fig. R3d).

Table R2. The range of phase parameters of HEA families.

HEAs	Ω^{-1} [a]	δr [b]	VEC [c]
Al-Ti-Nb-Ta-V-Hf	0.075–1.236	0.064–0.157	3.80–4.80
Al-Ti-Nb-Ta-V-Zr	0.087–1.447	0.064–0.157	3.80–4.80
Ti-Nb-Ta-V-Zr-Hf	0.000–0.094	0.032–0.061	4.15–4.85
Al-Nb-Ta-V-Zr-Hf	0.072–1.459	0.070–0.161	3.80–4.80
Al-Ti-Nb-Ta-W-Hf	0.099–1.170	0.061–0.158	3.85–5.15
Mo-Nb-Ta-W-Ti-Hf	0.000-0.171	0.024-0.055	4.40-5.60

[a] Ω^{-1} is a descriptor for predicting the phase stability of HEAs, as shown in Eq. (4); [b] δr is an atomic size mismatch that can be calculated by Eq. (9); [c] VEC is the average value of valence electron concentration that can be calculated by Eq. (8).

We have also supplemented the experimental results and method description of the compositional characterization. It was conducted using a Zeiss Sigma 500 scanning electron microscope (SEM) equipped with an Oxford UltimMax 65 energy-dispersive X-ray spectroscopy (EDS) system. The measured compositions, summarized in Table S3, are consistent with the nominal compositions for all samples.

Collectively, these extended experiments show that our AI predictions are not limited to the originally tested single composition. They work well for other compositions within the same HEA family and also transfer to a different refractory HEA system. The validated framework can therefore support future AI-driven discovery of high-performance HEAs across a wide range of compositional spaces.

Revised Section:

(1) Revisions in the Manuscript

Page 2: line 39 to line 42 (Abstract)

High-throughput screening of over 50 billion compositions and experimental validation across two distinct HEA families confirm the practical utility of our framework for AI-driven materials discovery.

Page 12: line 380 to line 383 (Section 2.3.3 Experimental method of allys)

Compositional characterization was performed using a Zeiss Sigma 500 scanning electron microscope (SEM) equipped with an Oxford UltimMax 65 energy-dispersive X-ray spectroscopy (EDS) detector.

Page 19 to Page 20: line 608 to line 634 (Section 3.3 AI-driven design of HEAs)

In addition, we also examined the Mo-Ti-Nb-Ta-W-Hf HEA family to further evaluate the generalizability of the AI predictions. The Mo-Nb-Ta-W quaternary family is a prototypical refractory high-entropy alloy (RHEA) family showing excellent high-temperature strength. For example, the equiatomic $Mo_{25}Nb_{25}Ta_{25}W_{25}$ HEA exhibits a yield strength above 400 MPa at 1600 °C [71]. However, these HEAs typically exhibit severe room-temperature brittleness. High-throughput screening reveals that the

addition of Ti and Hf to the Mo-Nb-Ta-W family can enhance its compressive plasticity to above 40%. According to our database, the predicted fraction of high-plasticity HEAs in this family is approximately 0.09%. Table 2 shows that the calculated parameters for the Mo-Ti-Nb-Ta-W-Hf family meet the phase criteria, confirming that this family also forms a single BCC solid solution. As listed in Table 3, we selected nine compositions that were predicted as "high" plastic label and BCC phase for experimental validation, including five Ti-Nb-Ta-V-Zr-Hf HEAs and four Mo-Ti-Nb-Ta-W-Hf HEAs.

The compressive stress-strain curves (Fig. 6a) reveal that all five Ti-Nb-Ta-V-Zr-Hf HEAs exhibit compressive ε_p exceeding 40%, in full agreement with their predicted high-plasticity labels. For the Mo-Ti-Nb-Ta-W-Hf family, two of the four selected compositions exhibit compressive ε_p above 40%, while the other two show moderate values of 36.5% and 23.5%, respectively (Fig. 6c). XRD patterns (Fig. 6b and Fig. 6d) confirm the single-BCC phase for all nine compositions. EDS mapping (Table S3) further confirmed that the measured compositions are consistent with the nominal values. The successful validation across two distinct HEA families demonstrates the generalizability of our AI predictions. The AI-driven screening over 50 billion compositions was completed in less than two hours, and the subsequent statistical analysis and data querying took only a few minutes. This high efficiency establishes a solid foundation for large-scale exploration and design of HEAs, enabling systematic investigation of previously less-accessible composition spaces.

Page 20: Revised Table 2. (Section 3.3 AI-driven design of HEAs)

Table 2. The range of phase parameters of HEA families.

HEAs	Ω^{-1} a)	δr b)	VEC c)
Al-Ti-Nb-Ta-V-Hf	0.075–1.236	0.064–0.157	3.80–4.80
Al-Ti-Nb-Ta-V-Zr	0.087–1.447	0.064–0.157	3.80–4.80
Ti-Nb-Ta-V-Zr-Hf	0.000–0.094	0.032–0.061	4.15–4.85
Al-Nb-Ta-V-Zr-Hf	0.072–1.459	0.070–0.161	3.80–4.80
Al-Ti-Nb-Ta-W-Hf	0.099–1.170	0.061–0.158	3.85–5.15

Mo-Nb-Ta-W-Ti-Hf	0.000-0.171	0.024-0.055	4.40-5.60

[a] Ω^{-1} is a descriptor for predicting the phase stability of HEAs, as shown in Eq. (4); [b] δr is an atomic size mismatch that can be calculated by Eq. (9); [c] *VEC* is the average value of valence electron concentration that can be calculated by Eq. (8).

Page 20: Additional Table 3. (Section 3.3 AI-driven design of HEAs)

Table 3. The predicted and experimental ε_p of the selected HEAs.

Sample ID [a]	Composition (at. %)	Prediction [b]	Experimental ε_p (%)
TNTVZH1	$Ti_{14}Nb_{18}Ta_8V_{13}Zr_{35}Hf_{12}$	High	>40
TNTVZH2	$Ti_{18}Nb_{18}Ta_{26}V_6Zr_{13}Hf_{19}$	High	>40
TNTVZH3	$Ti_{25}Nb_{18}Ta_8V_{24}Zr_{19}Hf_6$	High	>40
TNTVZH4	$Ti_{30}Nb_{18}Ta_5V_6Zr_{12}Hf_{29}$	High	>40
TNTVZH5	$Ti_{34}Nb_{30}Ta_5V_5Zr_{21}Hf_5$	High	>40
MTNTWH1	$Mo_5Ti_{25}Nb_{18}Ta_{16}W_6Hf_{30}$	High	36.5±0.5
MTNTWH2	$Mo_5Ti_{34}Nb_{30}Ta_9W_7Hf_{15}$	High	>40
MTNTWH3	$Mo_5Ti_{35}Nb_{31}Ta_{14}W_5Hf_{10}$	High	>40
MTNTWH4	$Mo_7Ti_{12}Nb_{11}Ta_{30}W_5Hf_{35}$	High	23.5±0.5

[a] TNTVZH and MTNTWH denote two distinct HEA families, Ti-Nb-Ta-V-Zr-Hf and Mo-Ti-Nb-Ta-W-Hf, respectively; [b] Predicted plasticity regime based on the GB classifier: High (ε_p > 40%).

Page 21: Revised Fig. 6 (Section 3.3 AI-driven design of HEAs)

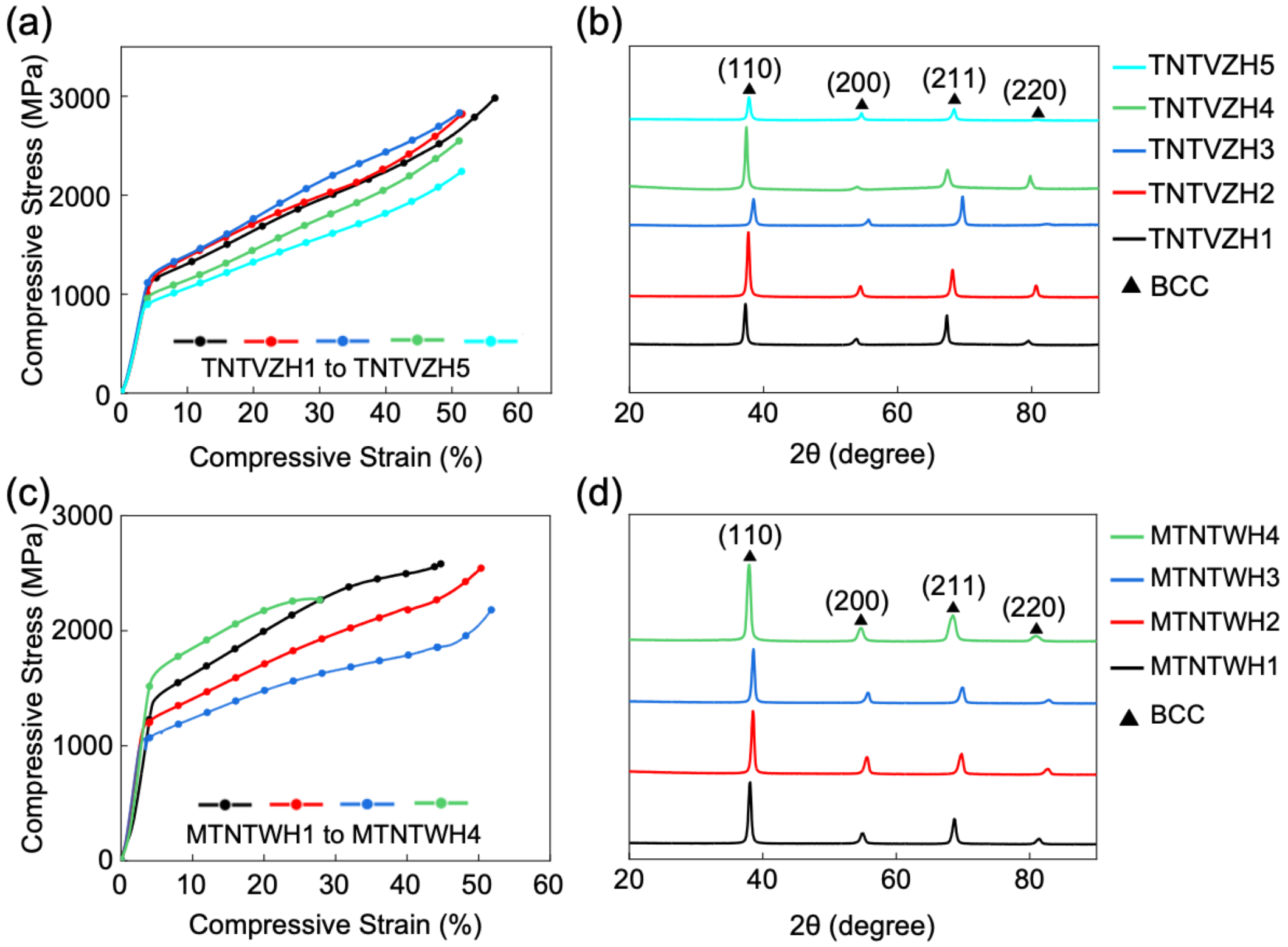

Fig. 6 Experimental validation of the selected HEAs. (a) and (b) Compressive stress-strain curves and XRD patterns for the Ti-Nb-Ta-V-Zr-Hf HEAs (labeled as TNTVZH HEAs). (c) and (d) Compressive stress-strain curves and XRD patternsfor the Mo-Ti-Nb-Ta-W-Hf HEAs (labeled as MTNTWH HEAs). The replicate test results for compressive stress-strain curves are provided in Figs. S8–S9.

Page 22: line 655 to line 658 (Summary and outlook)

By predicting the whole composition space and experimentally validating multiple compositions across two distinct HEA families, we successfully designed nine novel HEAs with high compression plasticity at room temperature.

Added references

[71] Senkov, O., Wilks, G., Scott, J. & et al. Mechanical properties of $Nb_{25}Mo_{25}Ta_{25}W_{25}$ and $V_{20}Nb_{20}Mo_{20}Ta_{20}W_{20}$ refractory high entropy alloys. *Intermetallics* **19**, 698–706 (2011).

(2) **Revisions in the Supplementary Information**

Page 13: Additional Fig. S8.

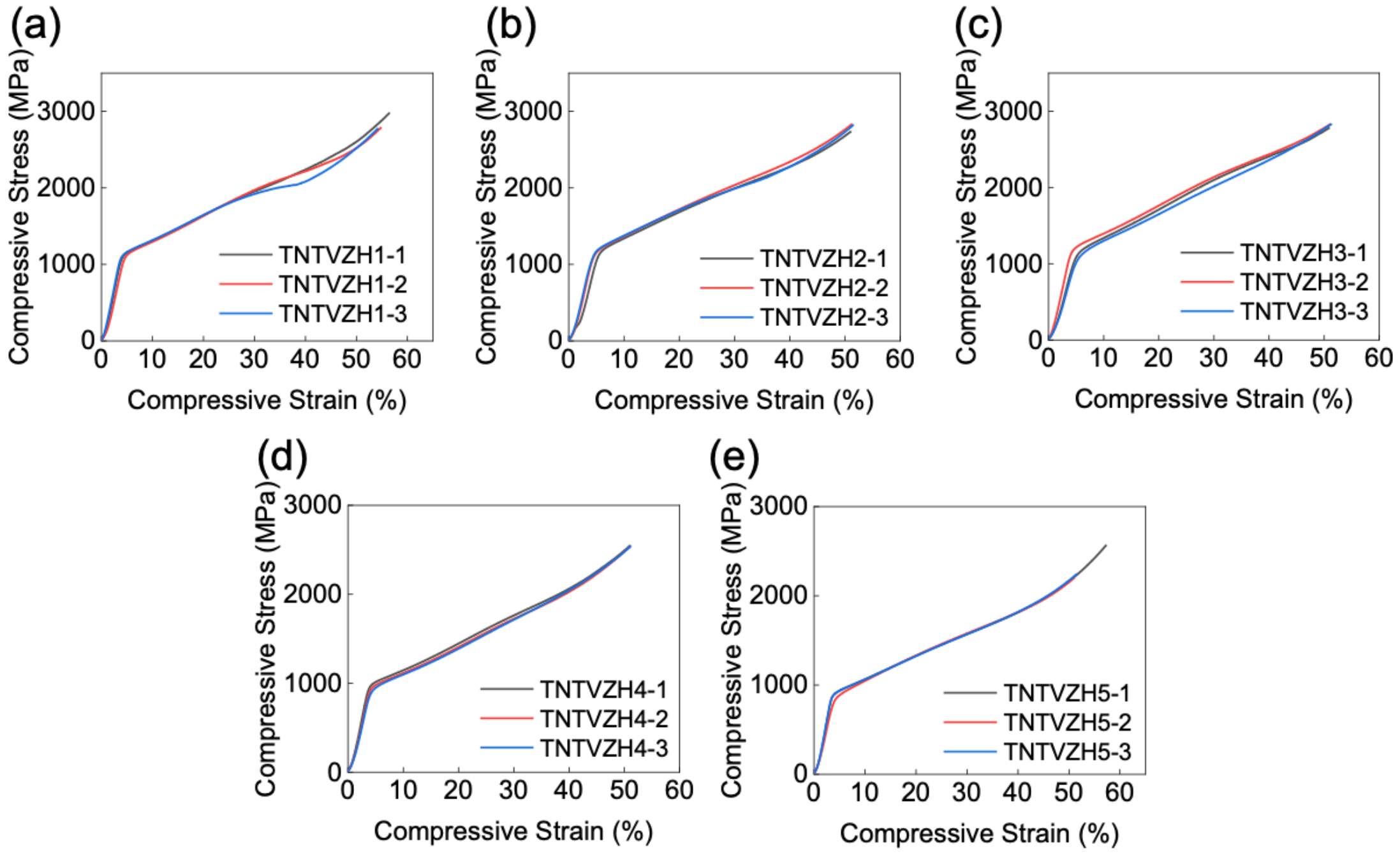

Fig. S8 Compressive stress–strain curves for the Ti–Nb–Ta–V–Zr–Hf (TNTVZH) high-entropy alloys. Subfigures (a)–(e) show the results for compositions TNTVZH 1 through 5, respectively. For each composition, the compression test was repeated three times using independent specimens to ensure reproducibility.

Page 14: Additional Fig. S9.

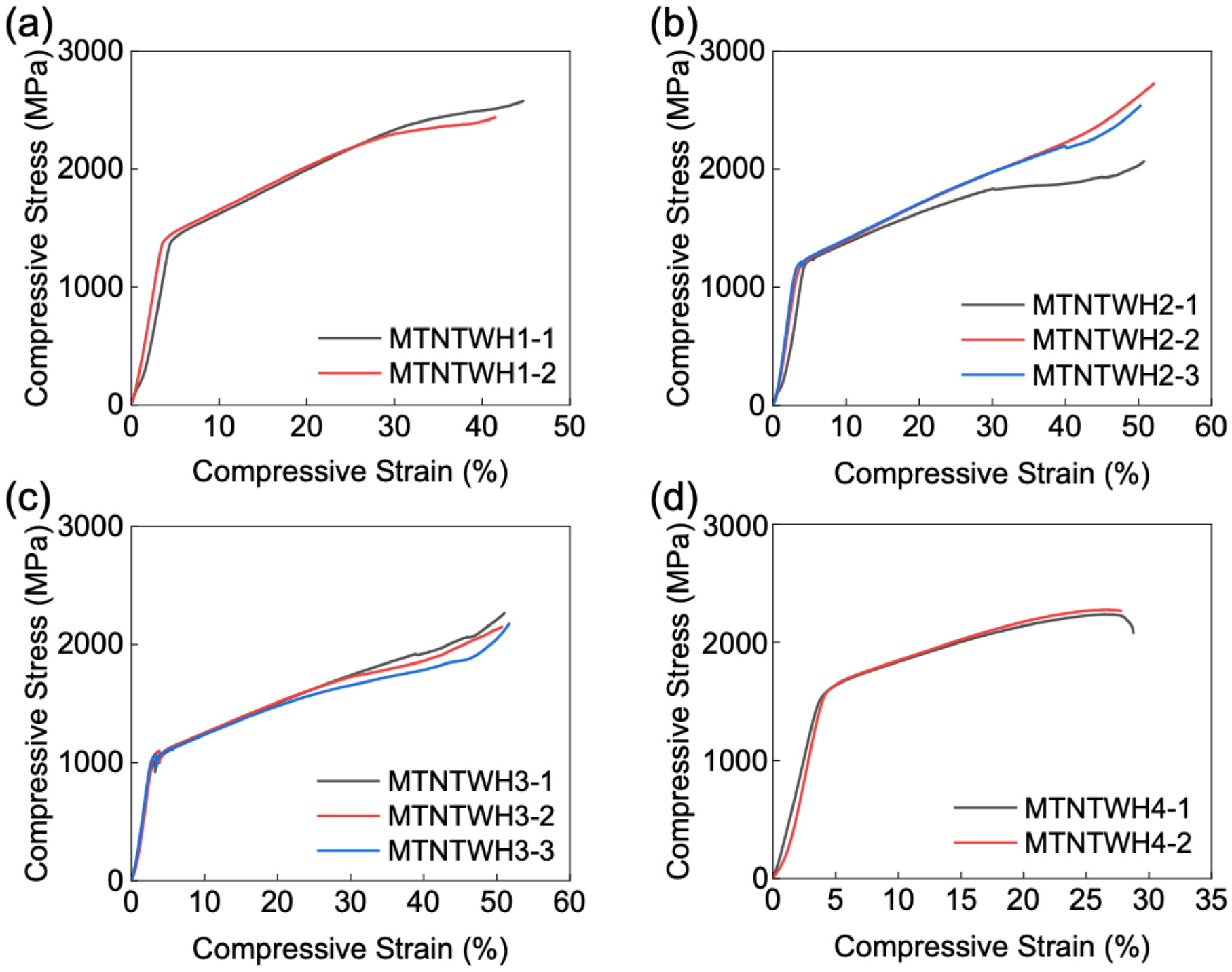

Fig. S9 Compressive stress–strain curves for the Mo-Ti-Nb-Ta-W-Hf (MTNTWH) high-entropy alloys. Subfigures (a)–(d) show the results for compositions MTNTWH 1 through 4, respectively. For each composition, the compression test was repeated two or three times using independent specimens.

Page 10: Additional Table S3.

Table S3. Experimental compositions (at. %) of the designed HEAs in this work.

Sample ID [a)]	Ti	Nb	Ta	V	Zr	Hf
TNTVZH1	14.2±0.1	17.2±0.1	9.0±0.2	13.0±0.0	34.8±0.0	11.8±0.0
TNTVZH2	18.4±0.0	17.0±0.1	26.1±0.1	6.3±0.1	13.6±0.1	18.6±0.1
TNTVZH3	25.2±0.0	17.2±0.1	8.6±0.1	24.0±0.1	19.2±0.1	5.7±0.1
TNTVZH4	30.6±0.1	17.0±0.0	5.8±0.1	6.3±0.0	12.5±0.1	27.8±0.0
TNTVZH5	34.0±0.1	29.0±0.1	6.1±0.1	5.1±0.0	20.9±0.1	4.9±0.0
Sample ID [a)]	Mo	Ti	Nb	Ta	W	Hf

MTNTWH1	4.7±0.0	25.9±0.5	16.8±0.2	16.1±0.4	6.9±0.3	29.5±0.5
MTNTWH2	4.9±0.1	32.5±0.5	29.5±0.2	10.7±0.3	8.9±0.2	13.4±0.3
MTNTWH3	4.9±0.1	34.2±0.1	29.8±0.1	15.5±0.1	6.3±0.1	9.4±0.1
MTNTWH4	6.7±0.2	12.8±0.2	10.0±0.3	29.3±0.3	5.8±0.2	35.5±0.3

[a)] TNTVZH and MTNTWH denote two distinct HEA families, Ti-Nb-Ta-V-Zr-Hf and Mo-Ti-Nb-Ta-W-Hf, respectively.

Question 7: The stateless design may not be optimal for iterative workflows (e.g., active learning) where state persistence is needed. Please comment on this.

Response: We thank the reviewer for this insightful suggestion. We agree that a stateless execution layer is not optimal for all iterative workflows, particularly when datasets are small enough to be processed entirely in memory. In such scenarios, a stateful design can indeed offer higher efficiency. However, for large-scale tasks such as the HEA screening reported in this work (covering 50 billion compositions), stateful execution becomes impractical, as the accumulation of intermediate states across iterations would rapidly exhaust available memory.

Our framework addresses this by shifting the persistence of intermediate states from memory to storage. The storage layer persistently manages intermediate results from each iteration, including prediction outcomes and iteration records. At the start of a new iteration, this information is retrieved from storage and fed back to the computation layer for model retraining and candidate selection. The stateless execution layer is equally important, as large-scale iterative tasks also face substantial computational demands. For example, in active learning for regression, each iteration typically requires multiple resampling procedures to estimate prediction variance, followed by model retraining with the expanded dataset. Our computation layer is well suited for these demands, enabling predictions across billions of compositions within hours. Together, the storage and computation layers address the two key bottlenecks of iterative workflows, state persistence and computational intensity, without the memory

growth or recovery overhead of stateful approaches, making this combined design a practical solution for large-scale iterative tasks.

We acknowledge that our original manuscript did not explicitly discuss iterative workflow support. Therefore, we have added a detailed discussion in Section 3.2.

Revised Section:

Revisions in the Manuscript

Page 17: line 564 to line 576 (Section 3.2 Advantages of the framework for the large-scale data)

The excellent performance of our framework in high-throughput computation and data management offers benefits for large-scale materials design. Beyond one-shot screening, it can also provide a robust foundation for iterative workflows such as active learning. For small-scale tasks where all data can be kept in memory, stateful execution can achieve better efficiency by caching intermediate results on compute nodes. As the task scale grows to billions of compositions, however, this approach becomes impractical. The accumulation of intermediate states in memory across iterations leads to unsustainable memory growth and eventual exhaustion. Our framework overcomes this barrier by decoupling computation from state management. The stateless computation layer, paired with a storage layer that persistently maintains intermediate results across rounds, enables iterative execution without the memory accumulation or recovery overhead typical of stateful designs. This separation allows the computational efficiency of our stateless framework to be fully leveraged in multi-round exploration.

Question 8: Crucial details of the material descriptors are missing. These include the detailed computational methods of G, G0, K, Tb and etc. According to the introduction in the 2.3.1 section, the material descriptors only include 10 materials descriptors with specific calculating formulas and 46 elemental parameters. There is no crystal structure parameters can be found, so how to evaluated the modulus, formation energy and vacancy migration energy of HEAs?

Response: We thank the reviewer for raising this point. We acknowledge that the description of the descriptor calculation methods and the treatment of crystal structure parameters was not sufficiently clear in the original manuscript. We address these issues in detail below.

We would like to clarify that all 194 descriptors used in this work, along with their detailed calculation methods, were already fully provided in the original manuscript and Table S2. The descriptors G, G_0, K, and T_b are included as part of the 46 elemental parameters and are computed using the composition-weighted averaging rule, where the average value is given by $\overline{F}=\sum_{i=1}^{n} c_i F_i$, with c_i and F_i denoting the mole fraction and the elemental parameter of the *i-th* element, respectively. Considering that the description in the original main text may not be sufficiently clear, we have revised Section 2.3.1 to provide a more explicit description of the calculation workflow and to direct readers to Table S2 for full details.

Regarding the crystal structure dependence of materials properties, such as modulus, vacancy formation energy, and vacancy migration energy, we have taken this into consideration when collecting elemental properties. Since our ML model targets the compressive plasticity of BCC HEAs, we attempted to collect elemental properties with the BCC structure. For lattice constants, we successfully obtained data for the BCC structure for all 15 elements. However, for other properties, including modulus, vacancy formation energy, and vacancy migration energy, BCC-specific data are unavailable in the literature or public databases for many elements. We therefore adopted data from the most stable phase of each element as a pragmatic alternative. While we recognize this is an approximation, such elemental descriptors have been widely used in the materials informatics community because they are computationally efficient and capture the intrinsic characteristics of constituent elements to a reasonable extent [R1-R4]. More accurate structure-specific descriptors derived from first-principles or molecular dynamics simulations would certainly improve predictive accuracy. However, for a compositional space of this scale, those approaches are computationally intractable. Therefore, developing accurate efficient descriptors that

are compatible with our high-throughput framework remains an important direction for our future work.

In the original manuscript, the elemental property data have been made publicly accessible via the GitHub repository (https://github.com/Wolido/hea_ducklake). In the revised version, we have further supplemented this with a new table (**Supplementary Datasets**) that provides literature references and crystal structure labels for each elemental parameter. We have also added corresponding descriptions in the revised manuscript to clarify the data provenance and structural basis of the descriptors used in this study.

Reference:

[R1] Roy, A., Babuska, T., Krick B. Machine learned feature identification for predicting phase and Young's modulus of low-, medium- and high-entropy alloys. *Scripta Materialia* **185**, 152–158 (2020).

[R2] Varvenne, C., Luque, Curtin, A., et al. Theory of strengthening in fcc high entropy alloys. *Acta Materialia* **118**, 164–176 (2016).

[R3] Rao, S., Akdemir, Z., Singh, P., et al. Theory of solid solution strengthening of BCC chemically complex alloys. *Acta Materialia* **209,** 116758 (2021).

[R4] Fan, Z., Xing, B. Cao, P. Predicting path-dependent diffusion barrier spectra in vast compositional space of multi-principal element alloys via convolutional neural networks. *Acta Materialia* **237**, 118159 (2022).

Revised Section:

Revisions in the Manuscript

Page 9: line 283 to line 285 (Section 2.3.1 Database contents and structure)

They are included in the 46 elemental parameters (detailed below) and are obtained by applying the composition-weighted averaging rule.

Page 10: line 311 to line 313 (Section 2.3.1 Database contents and structure)

The elemental properties used for descriptor calculations are given in the GitHub repository (https://github.com/Wolido/hea_ducklake) and Supplementary Datasets.

Page 10: line 313 to line 318 (Section 2.3.1 Database contents and structure)

The current descriptors are relatively simplified, yet they are widely adopted as an effective and computationally efficient approach in the field. With the computational efficiency of our framework, future work will incorporate more accurate descriptors from multi-scale calculations, such as melting temperature from CALPHAD or first-principles methods and shear modulus from first-principles calculations.

Reviewer #2:

Building on their previous work on machine learning applied to high-entropy alloys (HEAs), the authors of this manuscript extend this work to classes containing 6 different chemical species, drawn from a total of 15 elements. This leads to an exponential increase of combinations, which motivated the authors to develop a computational framework suitable for such large amounts of computations, data transfer, storage, and retrieval.

Hence, the present manuscript focuses on information technology with HEAs serving as a test case. As a consequence, the paper overwhelms the reader with a plethora of computer science related terms such as DataForge, stateless computing, Redis queues, docker, container, lakehouse architecture, DuckDB, Parquet storage, DuckLake cloud sharing, and S3 protocol.

Despite the interesting application to HEAs, the present manuscript seems to be more appropriate for a journal focusing on software architectures and algorithms in data science rather than Nature Communications. Thus, I do not recommend publication in Nature Communications.

In any case, the authors should consider the following points in any resubmission.

Response: We sincerely thank the reviewer for the time and effort devoted to our manuscript. In our prior work on HEAs, we found that their enormous composition space offers vast opportunities for exploration yet poses tremendous computational challenges. Our framework was developed specifically to address these challenges, but rather as a domain-driven solution where the unique combinatorial explosion of HEAs poses the central scientific bottleneck that we aim to break. The scientific goals and significance of this work are as follows.

First, this work addresses a critical challenge in the field. The compositional space of HEAs is immense, and AI-based predictions, along with experimental and theoretical approaches, face severe resource constraints when dealing with such a space. Our framework provides an efficient solution that improves computational, storage, and

query performance, substantially reducing resource consumption including time, memory, and disk usage. This facilitates large-scale exploration under realistic resource constraints. Second, we have completed a closed-loop validation. We performed an AI-driven design covering over 50 billion HEA compositions. This work validated the computational, storage, and query performance of our framework and demonstrated its capability for large-scale materials discovery. Third, this work provides an integrated infrastructure for future materials design. Our framework enables more sophisticated strategies such as, active learning, multi-objective optimization, and theoretical computation. This capability provides a foundation for the discovery of high-performance HEAs, and can be extended to other complex material systems. We have revised the main text accordingly, as detailed in the **Revised Section** below.

We also note that this work is submitted to the "Lightweight machine learning models and edge computing applications" Collection of Nature Communications. To further demonstrate the flexibility of our framework, we have added a performance comparison with several distributed computing frameworks (Spark, Dask, and Slurm) as shown in Response to Question 6, highlighting its lightweight design and adaptability to resource-constrained environments. We have also included tests on heterogeneous edge devices. These results demonstrate the scientific significance of our framework as a potential infrastructure for data processing and computation on edge devices in future self-driving laboratories.

We acknowledge that the original manuscript contained a number of computer science terms that may have hindered readability. To address this, we have provided brief explanations at the first appearance of each term in the main text and added a comprehensive glossary in the Supplementary Information (as shown in Response to Question 3).

We also provide point-by-point responses to all comments below, with all revisions highlighted in yellow and the section headings marked in green.

Revised Section:

Revisions in the Manuscript

Page 1: line 23 to line 27 (Abstract)

Artificial intelligence (AI) is accelerating materials discovery, yet for complex systems such as high entropy alloys (HEAs), exhaustive exploration of their immense composition spaces is computationally intractable. In this work, we propose an integrated framework that provides a pathway to large-scale exploration by substantially reducing resource demands.

Page 2: line 42 to line 44 (Abstract)

This work establishes a practical framework for resource-efficient materials discovery, serving as an integrated infrastructure for large-scale computational design.

Page 3: line 82 to line 84 (Introduction)

However, the vast composition space also renders the cost of theoretical calculations and AI predictions prohibitively high for exhaustive exploration under realistic resource constraints.

Page 4: line 123 to line 125 (Introduction)

Therefore, it is necessary to develop an efficient framework tailored to the scale and structure of complex material systems, which can serve as an essential infrastructure to support comprehensive exploration across composition spaces.

Page 21: line 636 to line 638 (Summary and outlook)

In this work, we proposed an integrated framework that provides a pathway to the computational exploration of enormous compositional spaces in complex material systems.

Page 22: line 658 to line 659 (Summary and outlook)

This result validates the practical utility of our framework for discovering materials across vast compositional spaces.

Page 22: line 662 to line 664 (Summary and outlook)

This framework establishes a robust foundation for computational exploration in compositional complex systems, enabling research avenues that were previously inaccessible due to resource constraints.

Question 1: Remove "DataForge" from the title.

Response: We thank the reviewer for this suggestion. We have removed "DataForge" from the title while keeping the original structure and emphasis. In addition, we have replaced "Architecture" with "Framework". While "architecture" emphasizes structural design, "framework" more accurately conveys the extensibility, reusability, and workflow-oriented nature of our work. The revised title is "Trillion-Scale Integrated Framework for High-Throughput Materials Databases and Seamless Sharing".

Revised Section:

Revisions in the Manuscript

Page 1: line 1 to line 3 (Title)

Trillion-Scale Integrated Framework for High-Throughput Materials Databases and Seamless Sharing

Question 2: Introduction: The first sentence is only partially true. The discovery of new materials is also, and perhaps even primarily, driven by a deeper understanding of the mechanisms leading to desired properties, aided by combined theoretical and computational efforts, as practiced, for example, by companies like QuesTek in the development of new steels.

Response: We thank the reviewer for this comment. We fully agree that the design of new materials is often driven by a deep understanding of mechanisms, supported by combined theoretical and computational efforts, as exemplified by QuesTek's success in developing high-performance materials through its integrated computational materials engineering (ICME) platform [The Bridge 54, 25-29 (2024); The Bridge 54, 30-37 (2024)]. We apologize that our original opening statement was not fully accurate, as it overlooked this important mechanism-driven pathway. Therefore, we have revised the opening sentence of the Introduction to acknowledge both empirical and mechanism-driven pathways in traditional materials design.

We are grateful to the reviewer for pointing out this oversight and believe the revised text now presents a more accurate view of the field.

References

[R1] Warren, J. The materials genome initiative and the metals industry. *The Bridge* **54**, 25-29 (2024).

[R2] Gong, J. Harnessing the power of AI in materials digital transformation: A synergistic hybrid approach. *The Bridge* **54**, 30-37 (2024).

Revised Section:

Revisions in the Manuscript

Page 2: line 48 to line 59 (Introduction)

Designing new materials has traditionally relied on empirical trial-and-error and mechanism-driven insight supported by theoretical and computational approaches, such as integrated computational materials engineering frameworks built for advanced alloy development [1-2]. In recent years, artificial intelligence (AI) has emerged as a data-driven methodology that offers a valuable complement to these traditional approaches [3-13]. By substituting physical experiments with rapid predictions, AI reduces the cost of iterative experimentation. It also mitigates the inherent limitations of mechanism-based paradigms, which frequently suffer from incomplete theoretical understanding or prohibitive computational costs when applied to complex systems. For instance, machine-learning interatomic potentials (MLIP) can accelerate atomistic simulations by orders of magnitude while maintaining near-first-principles accuracy [14, 15].

Added references

[1] Warren, J. The materials genome initiative and the metals industry. *The Bridge* **54**, 25-29 (2024).

[2] Gong, J. Harnessing the power of AI in materials digital transformation: A synergistic hybrid approach. *The Bridge* **54**, 30-37 (2024).

[10] Bai, S., Wan, D. & Tian, Y. et al. Designing advanced thermoelectrics. *Nature Reviews Materials* **32**, 931 (2026)

[11] Du, Z., Yuan, Y., Wang, L., & et al. Extreme electromagnetic wave absorption properties of graphene through machine learning. *Carbon* **256**, 121657 (2026).

[12] Liu, H., Nakata, T., Xu, C. & et al. Machine learning assisted design of high thermal conductivity and high strength mg alloys. *Metallurgical and Materials Transactions A* **56**, 1534–1551 (2025).

[13] Liang, X., Wand, H., Zhou, X. & et al. Revealing cryo-loading induced twinning and its impact on titanium via correlative data-driven mechanomicroscopy. *Acta Materialia* **309**, 122137 (2026).

[14] Deringer, V. L., Caro, M. A. & Csányi, G. Machine learning interatomic potentials as emerging tools for materials science. *Advanced Materials* **31**, 1902765 (2019).

[15] Behler, J. & Parrinello, M. Generalized neural-network representation of high-dimensional potential-energy surfaces. *Physical Review Letters* **98**, 146401 (2007).

Question 3: Not all readers of this article are necessarily familiar with terms like DuckLake cloud sharing. Hence, they should be properly explained, which is partially done, or references should be provided.

Response: We thank the reviewer for this constructive suggestion. We agree that technical terms such as DuckLake, and other related concepts may not be familiar to all readers. To improve accessibility, we have taken the following actions.

First, we have added a comprehensive glossary in the Supplementary Information (Table S1). For all key technical terms, including stateless computing, Redis queues, Docker containers, lakehouse architecture, DuckDB, Parquet storage, DuckLake cloud sharing, and the S3 protocol, the glossary provides concise definitions and describes their roles in our framework. This helps readers understand not only what each term means, but also why and how it is used in the framework.

Second, we have provided brief explanations at the first appearance of all key terms in the main text, so that readers can follow the technical discussion without repeatedly referring to the glossary.

Third, we have added relevant references in the main text.

We believe these revisions make the manuscript more accessible to a broad readership without compromising technical accuracy.

Revised Section:

(1) Revisions in the Manuscript

Page 4: line 146 to line 153 (Section 2.1 Framework overview and validation strategy)

Stateless computing enables each worker to process tasks independently, without retaining intermediate state between them. This design minimizes inter-node dependencies and I/O overhead, thereby facilitating elastic scaling and fault tolerance across distributed resources. Lakehouse architecture combines the low-cost scalable storage of data lakes with the structured querying capabilities of data warehouses [64]. It therefore supports efficient columnar storage, rapid analytical queries, and seamless cloud sharing of large scientific datasets.

Page 6: line 176 to line 184 (Section 2.2.1 Stateless parallel computation)

Redis List is a lightweight in-memory message queue that provides exceptionally fast read and write performance. It follows a producer-consumer pattern that decouples task generation from task processing [65]. Producers simply push jobs into the list, while consumers pull them from the other end. In our framework, Redis serves as the central task queue. Workers retrieve tasks from the queue independently, with no inter-worker communication or shared memory, making each worker stateless. Any available worker can perform any task, and the worker pool can be dynamically scaled without disrupting the workflow, ensuring optimal resource utilization across all computing nodes.

Page 6: line 189 to line 191 (Section 2.2.1 Stateless parallel computation)

Docker is an open-source containerization platform that encapsulates applications and their dependencies into portable containers, ensuring consistent execution across different environments [66].

Page 6: line 210 to line 211 (2.2.2 Lakehouse-based data management)

To optimize performance for subsequent analytical queries, the raw data is transformed and stored in Parquet, a columnar storage format that enables efficient selective column reads and achieves high compression ratios.

Page 6: line 215 to line 217 (2.2.2 Lakehouse-based data management)

DuckDB is an embeddable analytical database management system that executes SQL queries directly on Parquet files without requiring heavy-weight database servers [67].

Page 7: line 224 to line 226 (2.2.2 Lakehouse-based data management)

A comprehensive glossary of the key technical terms used in this work, along with their definitions and roles in the framework, is provided in Table S1.

(2) **Revisions in the Supplementary Information**

Page 4: Additional Table S1.

Table S1: Glossary of technical terms used in the integrated framework

Term	Definition	Role in the framework
Stateless parallel computing	A computing paradigm where each worker processes tasks independently without retaining any state between tasks.	Enables dynamic scaling and fault tolerance by decoupling task execution from state management.
Message queue	An asynchronous communication mechanism that decouples data producers from consumers.	In our framework, Redis List implements the message queue for task distribution and results collection.
Redis List	A first-in-first-out (FIFO) queue data structure provided by Redis.	Serves as upstream and downstream task queues in the parallel architecture.
Docker container	An isolated runtime environment that packages an application and its dependencies.	Runs as a stateless worker executing computational tasks.
Containerization	A technology that packages applications and dependencies into isolated containers.	Ensures environment consistency across heterogeneous computing nodes.
Worker	A computational unit (process or container) that fetches and executes tasks.	In our framework, workers are implemented as Docker containers that consume tasks from Redis queues.

Write Lock	A synchronization mechanism that controls write operations.	Prevents queue congestion when producers generate tasks faster than consumers can process.
Lakehouse architecture	An integrated data platform that combines the scalable storage of data lakes with the query capabilities of data warehouses.	Serves as the foundation of the data management module.
DuckLake	A metadata-driven data sharing layer built on Parquet files in object storage.	Enables remote dataset access via a small metadata catalog, avoiding full data transfer.
Metadata catalog	A structured record of dataset schema, file locations, and partitions.	Allows local querying without transferring entire datasets.
Object storage	A storage architecture that manages data as objects with unique identifiers.	Provides durable, scalable cloud storage for Parquet files.
S3 protocol	A RESTful API standard for object storage (Amazon S3 and compatible systems).	Enables HTTP-based remote access to shared databases.
Cloud sharing	Distribution of datasets and services via cloud infrastructure.	Enables seamless sharing of the HEA database among researchers.
Parquet	A columnar storage format with built-in schema and compression.	Stores HEA datasets and supports fast column retrieval.
Columnar storage	A storage layout where column values are stored contiguously.	Matches descriptor query patterns by reading only the required columns.
DuckDB	An in-process analytical database engine.	Queries Parquet files directly without requiring a separate database server.
Embedded analytical engine	A database engine that runs within the application process.	DuckDB uses this model to query Parquet files directly.
Query	A request to retrieve or analyze data.	Supports full database scans, column queries, and reverse queries on HEA data.
Reverse query	A query that finds records whose descriptor values match specified conditions.	Enables searching for HEA compositions within target descriptor ranges.
I/O (Input/Output)	Data transfer between the system and external storage or devices.	I/O (Input/Output)

Add references

[64] Databricks. What is a data lakehouse? https://docs.databricks.com/en/lakehouse (2024). Accessed: 2026-07-24.

[65] Redis. Redis Lists: *The list data type*. https://redis.io/docs/latest/develop/data-types/lists/ (2025). Accessed: 2026-07-24.

[66] Docker. Docker overview (2025). URL https://docs.docker.com/get-started/docker-overview/. Accessed: 2026-07-24.

Question 4: The authors claim a 90% storage reduction and 180 times faster queries compared with traditional frameworks. This is an empty claim unless both the traditional framework and the performance test are exactly defined.

Response: We thank the reviewer for raising this point. The descriptions of “a 90% storage reduction and 180 times faster queries compared with traditional frameworks” are given in the Abstract and the Summary and Outlook section. We fully agree that such performance claims must be accompanied by clear definitions of the test conditions. Therefore, we have made corresponding revisions in the manuscript, specifically to define the comparison baselines (CSV, JSON, and PostgreSQL) and the test conditions.

In Section 3.2 of the original manuscript, we have already provided the hardware specifications, dataset sizes, network conditions, and test scenarios for all performance comparisons. For further clarity, we have also supplemented the manuscript with more detailed definitions of the baseline approaches and the test procedures.

Revised Section:

Revisions in the Manuscript

Page 2: line 34 to line 38 (Abstract)

Our framework cuts runtime–memory cost by 45% versus distributed frameworks (Spark, Dask, Slurm), reduces storage by 90% for the full HEA database versus row-based formats (CSV, JSON), and accelerates subset queries by up to 180 times versus CSV and PostgreSQL.

Page 16: line 500 to line 506 (Section 3.2 Advantages of the framework for the large-scale database)

To demonstrate the storage efficiency of our framework, we also saved all calculated data for the six-component HEAs (Datasets 1 to 4) into two row-oriented formats commonly used in materials data management, CSV and JSON. As shown in Fig. 4c, Parquet required the least storage space at 17.5 TB, achieving up to 90% storage reduction compared with CSV (69 TB) and JSON (178 TB). JSON incurs additional overhead because it stores key names and structural delimiters for every record.

Page 16: line 513 to line 522 (Section 3.2 Advantages of the framework for the large-scale database)

To evaluate the performance of our framework on large-scale database queries, we compared CSV and PostgreSQL against our approach on both full-database and subset queries using the 17.5 TB HEA database. CSV represents the typical file-based workflow, and PostgreSQL serves as a representative row-oriented relational database. Before executing the queries, we converted the dataset into the corresponding formats required by CSV and PostgreSQL. We executed six representative query cases, as detailed in the Table 1 footnote and https://github.com/Wolido/hea_ducklake. Among them, the four subset queries are shown in Fig. 4d, while the remaining two full-database queries are summarized in Table 1.

Page 21: line 648 to line 651 (Section 4 Summary and outlook)

Compared with commonly used row-based formats (CSV, JSON), the storage volume was compressed by up to 90%. For subset queries on the six-component HEA database, our framework achieves speedups up to 180 times over CSV files and PostgreSQL.

Question 5: Stating that a full search across trillions of data in minutes (how many?) needs a specification of the processor, not only the memory usage.

Response: We thank the reviewer for this comment. We agree that this performance claim would be more precise and reproducible with both the exact query time and the processor specification clearly stated. We have revised the abstract to clarify that a full

search across more than 10 trillion data entries is completed in 7 minutes and 38 seconds utilizing less than 4 GB of memory. In Section 3.2, we further specify the processor and detail the query conditions. The full-database search was performed on a compute server equipped with an Intel Xeon Gold 6330 processor (56 cores at 2.00 GHz), 512 GB of RAM, and a 10 GbE interconnect (9000+ Mbps). These hardware and methodological details have been added to the revised manuscript, making the performance claim fully reproducible.

Revised Section:

Revisions in the Manuscript

Page 2: line 38 to line 39 (Abstract)

A full search of the ten-trillion-scale database finishes in 7 minutes and 38 seconds utilizing less than 4 GB of memory.

Page 11: line 350 to line 354 (Section 2.3.2 Implementation of the integrated framework)

This process was supported by 4 upstream and 4 downstream Redis queues, and utilized 500 CPU cores distributed across 10 compute nodes (each core running in a separate Docker container). The compute cluster comprised Intel Xeon Gold 6330 processors (56 physical cores per socket at 2.00 GHz; 50 cores utilized per node), 512 GB of RAM per node, and a 10 GbE interconnect providing over 9000 Mbps of internal bandwidth.

Page 17: line 543 to line 544 (Section 3.2 Advantages of the framework for the large-scale)

It was conducted on a compute server with the same configuration as the descriptor-generation nodes.

Question 6: The authors make the bold statement: “Existing solutions or technical frameworks are inadequate for the unique data structures and computational demands of materials science.” Can the authors really defend this statement in

view of the multitude of successful academic as well as commercial software solutions used in computational materials science?

Response: We thank the reviewer for this critical observation. We agree that the statement in our original manuscript was overly broad. What we intended to convey is that for large-scale materials tasks, modern frameworks are not fully equipped to handle the integrated demands of computing, storage, and data access. Therefore, we proposed an integrated framework that includes a stateless parallel computing model decoupling task distribution, execution, and result collection, a columnar storage format tailored for materials data, and a lakehouse-based access mechanism with small metadata directories for efficient retrieval. To substantiate the advantages of our framework, we have included comparisons with common storage formats (CSV, JSON) and access methods (CSV vs. PostgreSQL) in the manuscript, and have now added a comparison with several modern distributed frameworks (Spark, Dask, and Slurm) as detailed below.

Spark and Dask are execution-layer frameworks. Slurm is a resource scheduler, chosen as a baseline for its widespread use. All tests were performed on the same hardware. The cluster had 10 nodes, each with 56 Intel Xeon Gold 6330 cores at 2.00 GHz, 512 GB RAM, and a 10 GbE interconnect delivering over 9000 Mbps. We used all 56 cores per node instead of the 50 cores used in the original manuscript. This change avoided CPU swapping when using Dask, which would have added time overhead and reduced accuracy. We computed descriptors for over 50 billion compositions across 5,005 six-element HEA families using Spark, Dask, and our framework under this new configuration. For Slurm, we tested only one family with 10,762,647 compositions. Slurm relies mainly on disk I/O rather than in-memory processing. Full-scale computation would generate many random writes. That would cause excessive wear on storage media, especially SSDs, and shorten their lifespan. We therefore used one family as a representative baseline.

Fig. R1 summarizes the runtime and peak memory usage of each framework. Dask failed due to out-of-memory errors after about 9.2 hours, with each worker consuming

memory at a rate of 52.2 GB/h until reaching the 480 GB limit. Slurm finished in 29 seconds with a peak memory of 1.7 GB, but extrapolating to all 5,005 families would require about 40.3 hours, and this low memory footprint came at the cost of heavy disk I/O. Spark completed the task in 21.3 hours with 337.0 GB peak memory. We configured Spark to generate compositions on executors rather than on the driver, keeping the driver's workload limited to element combinations. This matches Spark's coarse-grained design and gives a fair baseline. Fine-grained management, where the driver tracks every stoichiometric composition, would create billions of tasks and quickly exceed the 512 GB node memory. This limitation is intrinsic to Spark's driver-centric model and would be worse for systems with seven or more elements. For Slurm, we kept the default settings without I/O optimization, representing standard HPC usage. Future work could apply our I/O strategies to enhance Slurm as well.

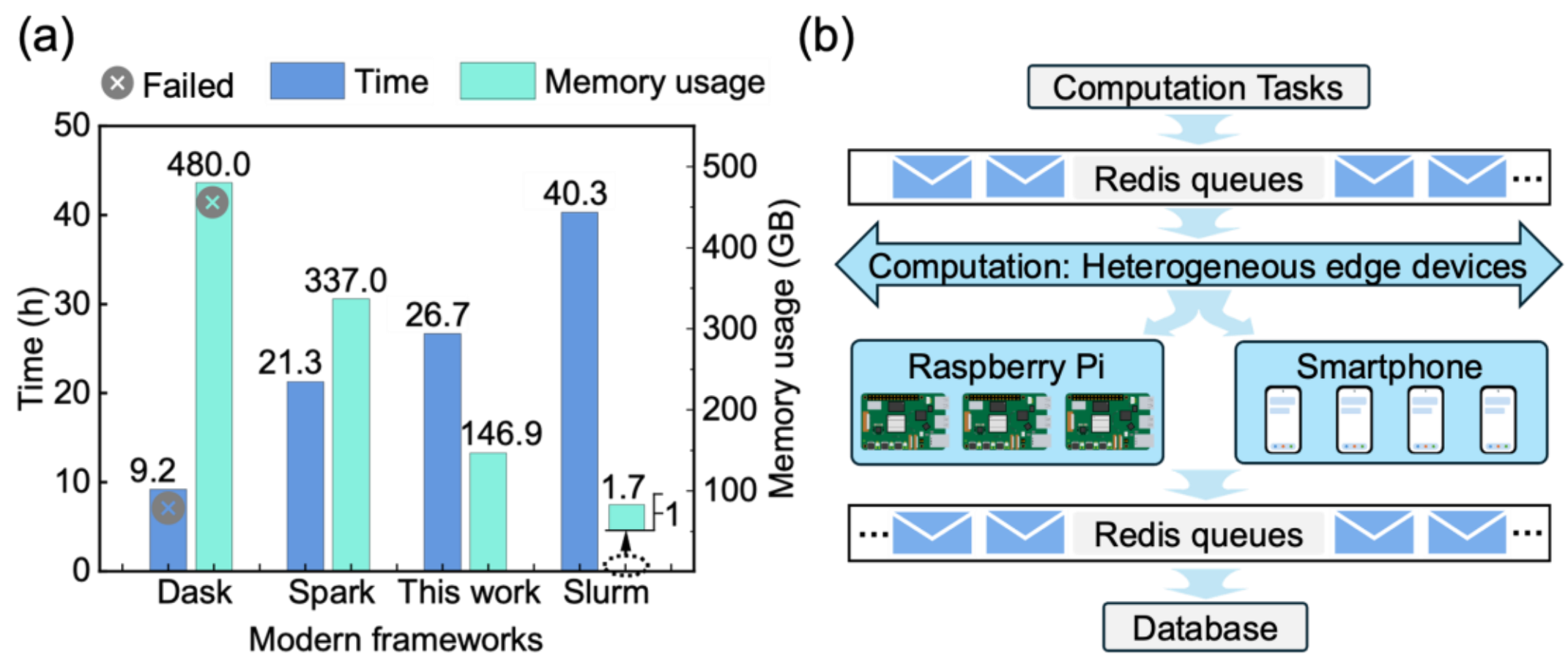

Fig. R1 (a) Performance comparison of Spark, Dask, Slurm, and our framework for the HEA descriptor computation. (b) Schematic illustration of the framework architecture with heterogeneous edge devices as compute nodes.

Our framework originally used a pull-per-task mode. Each Docker worker fetched one HEA composition from a Redis queue, computed descriptors, and pushed back the result. This consumed 30.1 MB per worker and about 1.7 GB per CPU node but took about one week for the full task. For a direct comparison with Spark, we used an alternative batch mode where each worker pulled 200,000 compositions at once. This reduced Redis overhead and finished in about 26.7 hours with a peak memory of 146.9

GB across the cluster. These two configurations demonstrate the flexibility of our framework in balancing memory consumption and computational speed. To measure overall resource efficiency, we used the product of runtime and peak memory. Dask failed due to memory exhaustion, and Slurm was excluded because its performance is I/O-bound. Spark had a product of about 7.18×10^3 GB·h (21.3 h × 337.0 GB). Our framework achieved about 3.97×10^3 GB·h (26.7 h × 146.9 GB), a 45% improvement. Overall, our framework offered better speed and memory efficiency than the other tools.

We further analyze the differences between these frameworks and our approach. Spark requires a dedicated runtime environment with a driver, executors, and a cluster manager. This runtime consumes baseline memory for scheduling, serialization, and shuffle services. That overhead is inherent to Spark's architecture and does not depend on task granularity. Dask workers are long-running processes that persist after each task. Memory pages allocated during execution are typically not returned to the operating system even after garbage collection. As a result, memory usage accumulates gradually over time, as shown in Fig. R2. Slurm behaves differently. Its memory footprint is low, about 1.7 GB in our test, because Slurm relies primarily on disk-based I/O and writes task outputs directly to disk. However, this approach generates many fragmented files and complicates data access and management. In our single-family test, tasks were evenly distributed across 560 cores and produced 560 separate output files. Extrapolating to all 5,005 HEA families would generate about 2.8 million files. Writing such a huge number of small files in bursts would introduce severe system-level bottlenecks. The metadata cache would face intense pressure, as frequent file creation and access would quickly exhaust the inode and dentry caches of the storage system, causing severe latency in metadata operations. In a full-scale run, even listing directory contents would become unresponsive. The post-processing stage would then require aggregating these massive files, which would consume substantial CPU and I/O bandwidth and become a critical bottleneck for overall data throughput.

Our framework operates on a different model. It does not require a dedicated runtime to stay active throughout the computation. The stateless workers consume memory only during active task execution, as shown in **Fig. R2**. This keeps memory

footprints very low in the single-task configuration. Task distribution uses Redis as a lightweight queue, so the system does not require high-speed internal networking among worker nodes. As a result, the framework is well suited for heterogeneous and resource-constrained edge devices. To verify this flexibility, we successfully tested two heterogeneous edge devices as compute nodes, as illustrated in Fig. R1b, which are a Raspberry Pi Zero 2W powered by an ARM Cortex-A53 processor with 512 MB RAM and a smartphone equipped with an ARM Cortex-A520 processor with 16 GB RAM. They successfully connected to the same Redis queue simultaneously, pulled subtask data from it, and completed the calculations. For most edge devices, we recommend running the computation inside Docker containers to ensure fast deployment and environment consistency. For devices that cannot run Docker, such as the smartphone used in this test, the computation code can be executed directly on the device without containerization. These results confirm that the framework can effectively utilize a wide range of computing resources, from dedicated cluster nodes to heterogeneous edge devices.

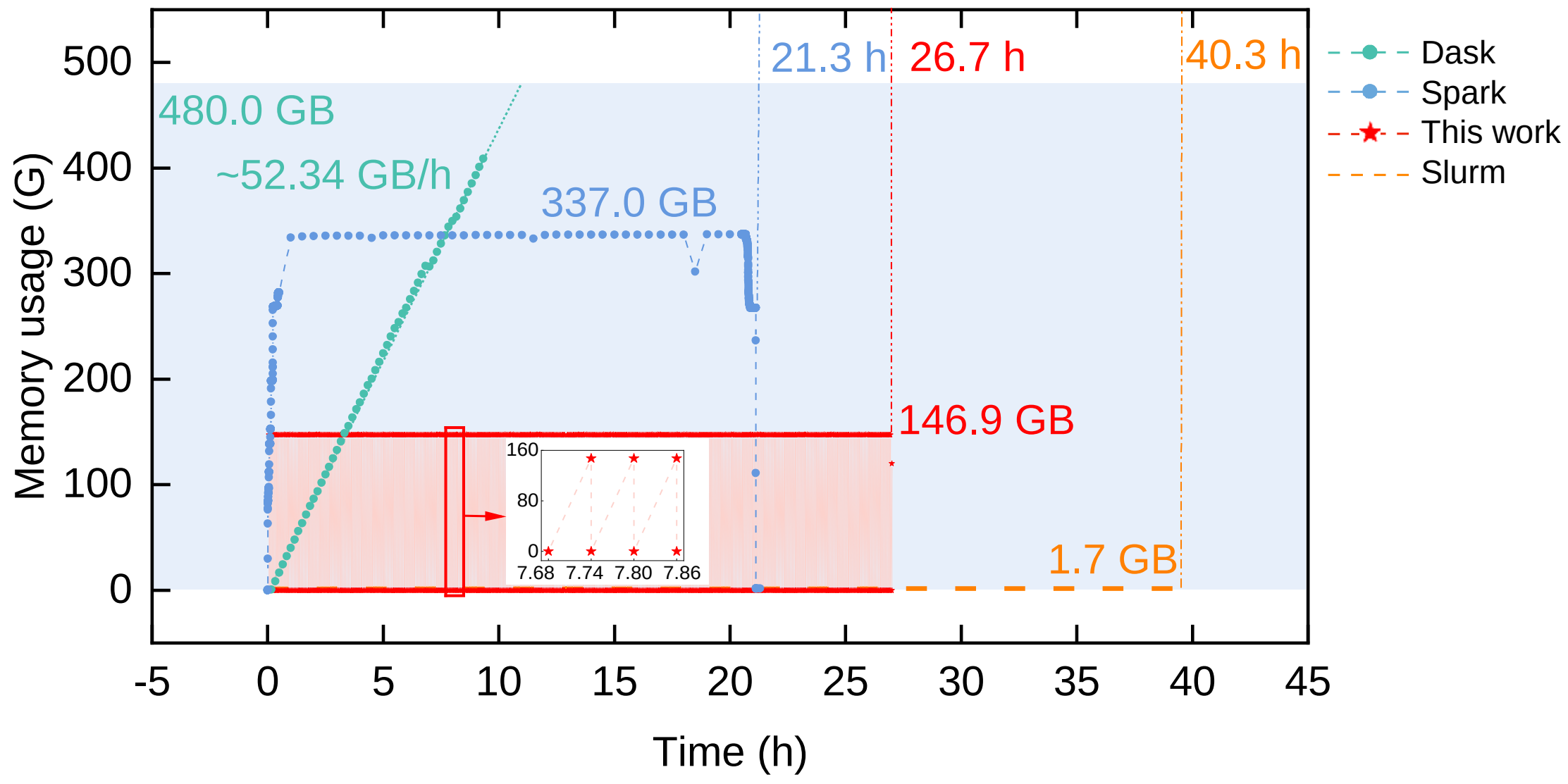

Fig. R2 Memory usage per compute node over runtime for Spark, Dask, Slurm, and our framework during the HEA descriptor computation. The embedded zoomed-in panel displays our framework's memory behavior across three Docker worker runtime cycles.

We have revised the overstated statement in the Introduction and supplied the benchmark comparisons to the revised manuscript and the Supplementary Information.

Revised Section:

(1) Revisions in the Manuscript

Page 2: line 34 to line 38 (Abstract)

Our framework cuts runtime–memory cost by 45% versus distributed frameworks (Spark, Dask, Slurm), reduces storage by 90% for the full HEA database versus row-based formats (CSV, JSON), and accelerates subset queries by up to 180 times versus CSV and PostgreSQL.

Page 3: line 99 to line 100 (Introduction)

However, few existing solutions are specifically designed to tackle these challenges in an integrated manner for large-scale materials tasks.

Page 12 to Page 13: line 407 to line 409 (Section 3.2 Advantages of the framework for the large-scale database)

Using this framework, high-throughput calculation tasks of more than 10 trillion materials descriptors were completed in one week.

Page 13 to Page 16: line 410 to line 491 (Section 3.2 Advantages of the framework for the large-scale database)

To further quantify these advantages, we evaluated the performance of our framework against several representative distributed platforms, including Spark and Dask as execution-layer frameworks, and Slurm as a resource scheduler. Slurm in particular was selected as a baseline, given its widespread adoption in academic and research computing environments. All tests were performed using 10 nodes with 56 cores per node, rather than the 50 cores per node used previously. This adjustment avoided CPU swapping that would otherwise arise when running Dask on partially allocated cores, introducing additional time overhead and compromising measurement accuracy. We executed Spark, Dask, and our framework to compute materials descriptors for over 50 billion compositions across 5,005 six-element HEA families. For Slurm, we restricted

the test to a single family of 10,762,647 compositions to avoid excessive wear on storage media, as Slurm relies primarily on disk-based I/O rather than in-memory processing.

As shown in Fig. 4a, Dask failed to accomplish the task. Memory usage on each worker grew at a quasi-linear rate of about 52.2 GB per hour, and the configured limit of 480 GB was reached after roughly 9.2 hours, resulting in out-of-memory errors (Fig. S7). Slurm finished a single HEA family of 10.7 million compositions in about 29 seconds, with a peak memory footprint of approximately 1.7 GB. Extrapolated to all 5,005 families, the estimated runtime would be around 40.3 hours. Spark completed the computation in approximately 21.3 hours with a peak memory usage of 337.0 GB. For our framework, when each Docker worker pulled one HEA composition at a time, the memory consumption was only 30.1 MB per worker, totaling approximately 1.7 GB across a CPU node (56 cores). While this setup minimizes memory usage and is viable on resource-constrained edge devices, the Redis communication overhead becomes significant at the scale of billions of compositions. To increase throughput and enable a direct comparison with Spark, we adopted an alternative configuration in which each worker pulled 200,000 HEA compositions per batch. This approach reduced the Redis overhead and completed the computation in about 26.7 hours, with a peak cluster-wide memory usage of 146.9 GB. These two configurations illustrate the tunable balance between memory footprint and computational speed that our framework offers. It should be noted that the result of Spark is optimized by shifting composition generation from the driver to the executors, such that the driver only handles element combinations instead of billions of individual compositions. This is consistent with Spark's coarse-grained design and offers a fair performance baseline. By contrast, if the driver were to manage fine-grained tasks involving individual HEA compositions, the task count would climb to billions and push memory usage well beyond the 512 GB node limit, eventually causing job failure. We used the default Slurm configuration without I/O tuning, which is the typical setup in most research. Overall, our framework outperformed Slurm and Dask in efficiency and achieved a runtime comparable to that of Spark while using considerably less memory. To provide a quantitative comparison

of overall resource efficiency, we used the product of runtime and peak memory usage as a combined metric. Dask failed to complete the computation due to memory exhaustion, and Slurm was excluded from this metric because its performance is dominated by disk I/O rather than memory usage. The time-memory product for Spark was approximately 7.18×10^3 GB·h, while our framework achieved approximately 3.97×10^3 GB·h, representing a 45% reduction.

The architecture underlying these performance gains is as follows. Spark requires a dedicated runtime environment that consumes baseline memory for scheduling, serialization, and shuffle services regardless of task granularity. Dask workers are long-running processes that retain memory pages even after garbage collection, causing memory to accumulate over time. Slurm, while memory-efficient, relies primarily on disk-based I/O and writes task outputs directly to disk, generating fragmented files that complicate data aggregation across multiple nodes. In our single-family test, tasks were evenly distributed across 560 cores, producing 560 output files. Extrapolating to the full set of 5,005 HEA families would produce roughly 2.8 million files, placing intense pressure on the metadata cache and exhausting the filesystem caches of the storage system. The post-processing stage would then require aggregating this massive number of files, which would consume substantial CPU and I/O bandwidth and ultimately become a critical bottleneck for overall throughput. In this work, we used Redis queues for task distribution and result collection, with task executions completed in Docker containers. The upstream and downstream nodes of the containers are both Redis queues, providing an isolated and stateless execution environment. Workers consume memory only during active task execution (Fig. S7), and task distribution through Redis eliminates the need for high-speed internal network interconnects among worker nodes. The stateless design of our framework inherently supports elastic scaling of compute nodes and cross-cluster distributed computing, allowing resources to be aggregated across physical boundaries without I/O overhead or memory accumulation. The two test configurations demonstrate the flexibility of our framework in balancing memory usage and computational time. Therefore, it also accommodates heterogeneous and resource-constrained environments, including edge devices. We tested two edge

devices as compute nodes, a Raspberry Pi Zero 2W powered by an ARM Cortex-A53 processor with 512 MB RAM and a smartphone equipped with an ARM Cortex-A520 processor with 16 GB RAM, as shown in Fig. 4b. Both devices successfully connected to the same Redis queue, pulled subtask data, and completed the descriptor calculations. For most edge devices, we recommend running computations inside Docker containers to ensure fast deployment and environment consistency. For devices that cannot run Docker, such as the smartphone used in this test, the computation code can be executed directly. Our framework can also be integrated into existing workflow orchestration systems such as Nextflow, though it remains fully functional and deployable independently without such orchestration. These results confirm that the framework can effectively utilize a wide range of computing resources, from dedicated cluster nodes to edge devices. More details are provided in the Supplementary Information.

Page 15: Revised Fig.4

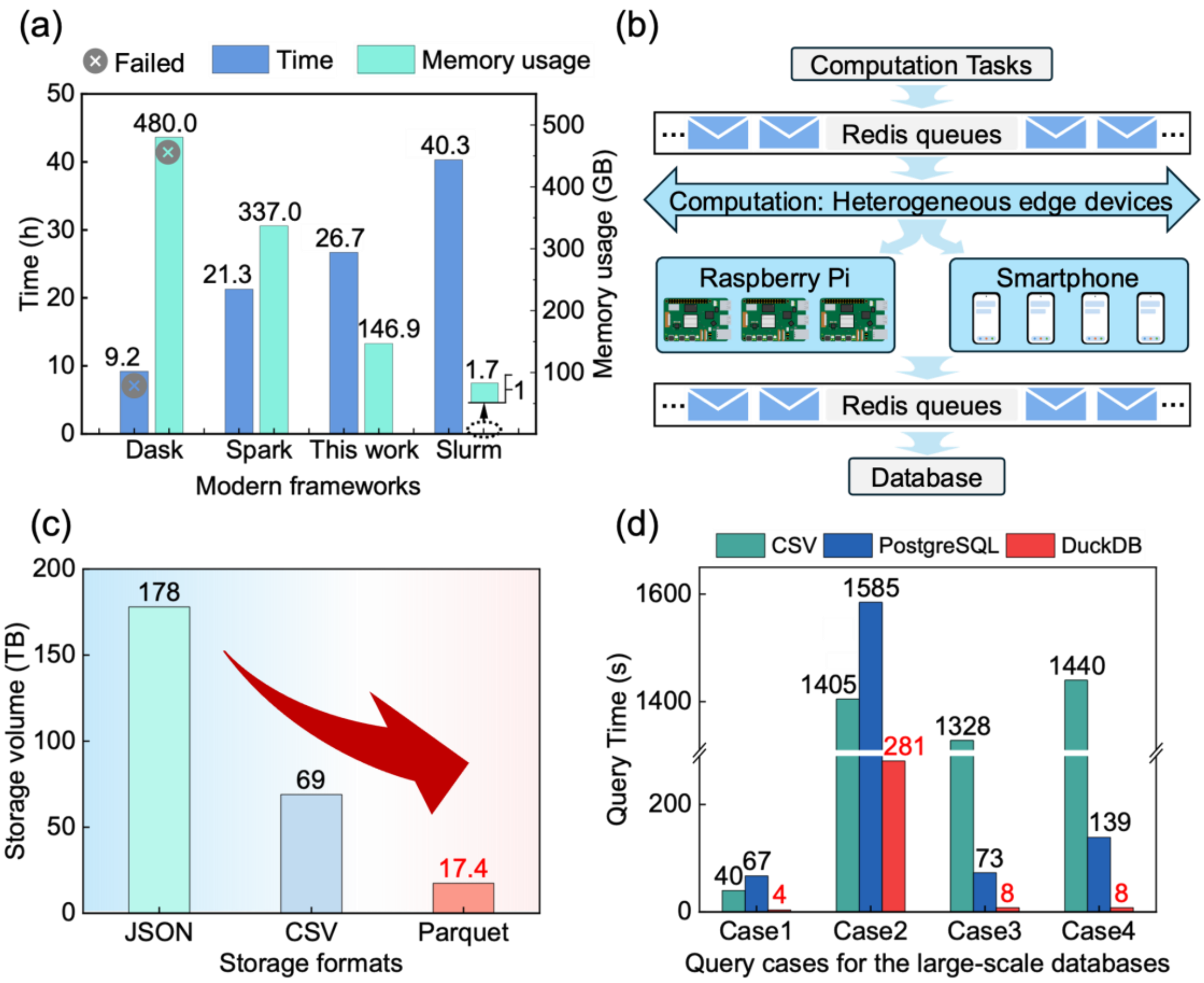

Fig. 4 Performance benchmark of distributed frameworks and data management architectures for large-scale materials data. (a) Runtime and peak memory usage for Spark, Dask, Slurm, and our framework on the HEA descriptor computation. (b) Schematic illustration of the framework architecture with heterogeneous edge devices as compute nodes. (c) Storage volume comparison between columnar Parquet, row-based formats (CSV and JSON), and PostgreSQL. (d) Query speeds for four HEA cases using CSV, PostgreSQL, and DuckDB. The four query cases are defined in Table 1.

Page 21: line 646 to line 648 (Summary and outlook)

Benchmarking against modern distributed frameworks, including Spark, Dask, and Slurm, shows that our framework achieves a 45% reduction in the combined time-memory metric.

Page 22: line 653 to line 655 (Summary and outlook)

We have also validated the framework on heterogeneous edge devices, confirming its adaptability to resource-constrained environments beyond conventional computing clusters.

Page 22: line 681 to line 683 (Summary and outlook)

This edge-computing capability also lays the groundwork for integrating the framework into self-driving laboratory environments for real-time experimental feedback.

(2) Revisions in the Supplementary Information

Page 13: Additional Fig. S7.

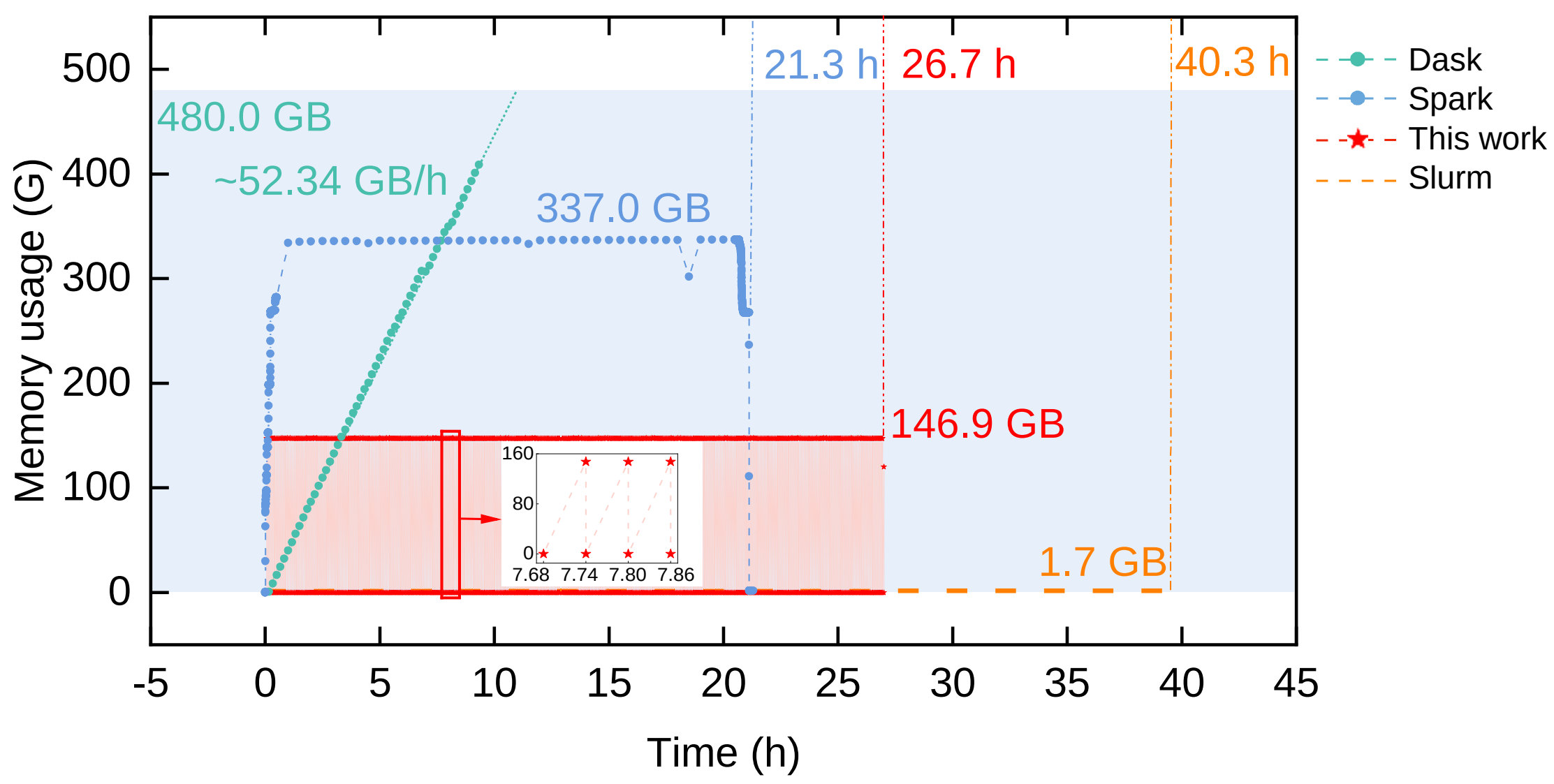

Fig. S7 Memory usage per compute node over runtime for Spark, Dask, Slurm, and our framework during the HEA descriptor computation. The embedded zoomed-in panel displays our framework's memory behavior across three Docker worker runtime cycles.

Page 7: line 125 to line 131 (Section S7.1 Spark configuration)

The Spark test used Apache Spark 3.5.8 with a mapPartitions architecture. Composition generation and descriptor computation were performed locally within each executor. The output was written as Parquet files compressed with Snappy, totaling 23 TB. Spark runs on the Java Virtual Machine, and each executor is a JVM process that manages task execution and data storage. The JVM heap stores task scheduling information, partition metadata, and serialization buffers. Each executor processed one full six-element family, corresponding to approximately 10.7 million compositions.

Page 7: line 133 to line 134 (Section S7.2 Dask configuration)

Dask 2025.1.0 was applied to the performance test. The workload was partitioned into 101 groups of 50 families, each containing approximately 535 million compositions.

Page 7: line 136 to line 138 (Section S7.3 The computations by Slurm)

Our single-family test was executed on Slurm 19.05.3-2, with all composition calculations evenly distributed across 560 CPU cores, generating 560 separate output files.

Question 7: Why do the authors discuss at length all 194 descriptors when at the end only five simple descriptors derived from the valence electron count and atomic radii are used as input features?

Response: We thank the reviewer for raising this point. The 194 descriptors form a reusable feature library covering thermodynamic, kinetic, electronic, atomic-size, mechanical, and crystallographic properties of elements. It can support various materials property modeling tasks beyond this work. The five descriptors in Section 2.3.1 are inputs to a ML model that predicts room-temperature compressive plasticity of as-cast BCC HEAs. They were selected as the optimal subset for this property through feature engineering (Huang et al., Mater. Des. 211, 110177, 2021). In addition, we used three other descriptors from the same library (Ω, δr, and VEC) as phase-screening criteria to identify BCC solid-solution candidates, as described in the manuscript. These uses illustrate that the 194-descriptor library is built as a foundational resource for alloy design, suitable for applications beyond the plasticity model. Its size also offers a practical testbed for benchmarking our framework. The manuscript has been revised to further clarify this point.

Revised Section:

Revisions in the Manuscript

Page 8: line 256 to line 261 (Section 2.3.1 Database contents and structure)

These descriptors form a comprehensive and reusable feature library covering thermodynamic, kinetic, electronic, atomic-size, mechanical, and crystallographic properties. The library supports multiple HEA design tasks, such as the plasticity modeling and phase structure design presented in Section 3.3. It thus provides a foundational resource for our future HEA studies.

Question 8: It is not clear how properties such as mixing enthalpy are computed. Do they involve DFT calculations of binary system? Are they taken from CALPHAD approaches?

Response: We thank the reviewer for raising this question. All 194 descriptors were calculated using the formulas listed in Table S1, combined with the composition ratios and elemental properties. The elemental properties used in this work were compiled from multiple published sources, including DFT calculations, CALPHAD assessments, and experimental measurements. These data have been made publicly available in the GitHub repository (https://github.com/Wolido/hea_ducklake) provided in the original manuscript. In the revised version, we have further supplemented a new table (**Supplementary Datasets**) that provides the literature sources for all elemental properties used, including the mixing enthalpy. The binary mixing enthalpy data are taken from the published work (Takeuchi, A., 2005), where the mixing enthalpies of binary systems were calculated based on Miedema's scheme.

Reference

[R1] Takeuchi, A. & Inoue, A. Classification of Bulk Metallic Glasses by Atomic Size Difference, Heat of Mixing and Period of Constituent Elements and Its Application to Characterization of the Main Alloying Element. *Materials Transactions* **46**, 2817–2829 (2005).

Revised Section:

Revisions in the Manuscript

Page 10: line 311 to line 313 (Section 2.3.1 Database contents and structure)

The elemental properties used for descriptor calculations are given in the GitHub repository (https://github.com/Wolido/hea_ducklake) and Supplementary Datasets.

Question 9: Similarly, the computation of vacancy formation energies involving two elements should be explained.

Response: We thank the reviewer for pointing this issue. We apologize that the formula for vacancy formation energy was not clearly explained in the original manuscript. The calculation of the binary vacancy formation energy follows the method described by Ji and Pan (Mater. Sci. Eng. A, 2008), which is based on the Miedema model.

The binary vacancy formation energy H_{ij} is calculated as follows:

$$H_{ij} = (1\text{-}f_{ij})H_i + f_{ij}H_j(\frac{V_i}{V_j})^{5/6} \quad \text{if } V_i > V_j$$

$$H_{ij} = (1\text{-}f_{ij})H_j + f_{ij}H_i(\frac{V_j}{V_i})^{5/6} \quad \text{if } V_i \leq V_j$$

where V_i and V_j are the molar volumes of elements i and j; H_i and H_j are are the monovacancy formation energies of pure elements i and j; f_{ij} is a weighting factor defined as:

$$f_{ij} = C_j(1+5(C_iC_j)^2) \quad \text{if } V_i > V_j$$

$$f_{ij} = C_i(1+5(C_iC_j)^2) \quad \text{if } V_i \leq V_j$$

with the concentration weights:

$$C_i = \frac{x_i V_i^{2/3}}{x_i V_i^{2/3} + x_j V_j^{2/3}}$$

$$C_j = \frac{x_j V_j^{2/3}}{x_i V_i^{2/3} + x_j V_j^{2/3}}$$

The molar volumes and monovacancy formation energies of pure elements were included in the GitHub repository (https://github.com/Wolido/hea_ducklake) and Supplementary Datasets. We have also added the detailed descriptions to the Supplementary Information and included the reference.

Reference

[R1] Ji, X. & Pan, Y. Predicting alloy compositions of bulk metallic glasses with high glass-forming ability. *Materials Science and Engineering: A* **485**, 154–159 (2008).

Revised Section:

Revisions in the Manuscript

Page 10: line 310 (Section 2.3.1 Database contents and structure)

A full list of all 194 material descriptors, along with more detailed descriptions, is provided in Supplementary Information (Table S1).

Revisions in the Supplementary Information

Page 5 to Page7: line 111 to line 119 (Section S5 The materials descriptors of HEAs)

It should be noted that the binary vacancy formation energy for the *i*-*j* pair, H_{ij}, is calculated as follows.

$$H_{ij} = (1\text{-}f_{ij})H_i + f_{ij}H_j(\frac{V_i}{V_j})^{5/6} \quad \text{if } V_i > V_j \tag{1}$$

$$H_{ij} = (1\text{-}f_{ij})H_j + f_{ij}H_i(\frac{V_j}{V_i})^{5/6} \quad \text{if } V_i \le V_j \tag{2}$$

where V_i and V_j are the molar volumes of elements *i* and *j*, H_i and H_j are the monovacancy formation energies of pure elements *i* and *j*, respectively. The weighting factor f_{ij} is defined as follows.

$$f_{ij} = C_j(1+5(C_iC_j)^2) \quad \text{if } V_i > V_j \tag{3}$$

$$f_{ij} = C_i(1+5(C_iC_j)^2) \quad \text{if } V_i \le V_j \tag{4}$$

where the concentration weights are obtained by:

$$C_i = \frac{x_iV_i^{2/3}}{x_iV_i^{2/3} + x_jV_j^{2/3}} \tag{5}$$

$$C_j = \frac{x_jV_j^{2/3}}{x_iV_i^{2/3} + x_jV_j^{2/3}} \tag{6}$$

Where x_i and x_j being the atomic fractions of elements *i* and *j*. The molar volumes and monovacancy formation energies of pure elements were included in the GitHub repository (https://github.com/Wolido/hea_ducklake) and Supplementary Datasets.

Page 10: Additional reference

[2] Ji, X. & Pan, Y. Predicting alloy compositions of bulk metallic glasses with high glass-forming ability. Materials Science and Engineering: A 485, 154–159 (2008).

Question 10: The approximation of the melting temperature as a weighted average of melting temperatures of pure elements is rather crude, as it ignores, for example, eutectic behavior.

Response: We thank the reviewer for this comment. We agree that using a simple weighted average of pure-element melting temperatures is a crude approximation. This

simplified approach does not account for the effects of microstructure, such as eutectic. It was chosen for its computational efficiency, as accurate determination of melting temperatures for billions of compositions using CALPHAD or first-principles methods would be impractical. Despite its limitations, this approach has been widely used in HEAs studies and provides a reasonable first-order estimate for high-throughput screening purposes. Future work may incorporate more accurate thermodynamic models once the computational cost is reduced through the acceleration capabilities of our framework.

Revised Section:

Revisions in the Manuscript

Page 10: line 311 to line 318 (Section 2.3.1 Database contents and structure)

The current descriptors are relatively simplified, yet they are widely adopted as an effective and computationally efficient approach in the field. With the computational efficiency of our framework, future work will incorporate more accurate descriptors from multi-scale calculations, such as melting temperature from CALPHAD or first-principles methods and shear modulus from first-principles calculations.

Question 11: How would the approach scale for seven- or eight-component systems? Perhaps to this end, the authors could perform tests for five- and four-component systems and analyze the trends.

Response: We thank the reviewer for this question regarding the scalability of our approach to higher-order systems. The number of principal components directly determines the size of the composition space of HEAs and, consequently, the computational workload for our framework. We further discuss the application of our framework to systems with four to eight principal components below.

We calculated the number of HEAs with different principal-component counts from a pool of 15 elements, as shown in **Fig. R3**. In the original manuscript, a six-component system with each element varying from 5 to 35 at% in 1 at% steps yield 10,762,647 compositions. For other component counts, the numbers are 11,461 for four,

553,401 for five, 116,352,103 for seven, and 802,866,768 for eight components. The number of distinct systems is 1,365 for four, 3,003 for five, 5,005 for six, 6,435 for seven, and 6,435 for eight components. Therefore, considering all combinations of 15 elements, the total number of compositions reaches approximately 1.6×10^{7}, 1.7×10^{9}, 5.4×10^{10}, 7.5×10^{11}, and 5.2×10^{12} for four to eight components, respectively. These data illustrate the combinatorial explosion as the number of components increases.

To demonstrate the scalability of our framework to higher-dimensional composition spaces, we selected the seven-component HEA system as a representative case for evaluation. We computed 194 descriptors for the Al-Cr-Mo-Nb-Ta-W-Hf system using the same cluster configuration as in the main text, which consists of 10 nodes, 560 cores, four Redis queues, and Docker workers. The computed descriptors for this single seven-component system were stored as Parquet files, occupying 57 GB of storage. Extending this calculation to all 6,435 seven-component systems would require approximately 358 TB of storage. By extrapolation, a full exploration of the eight-component space would demand over 3,000 TB, where resource constraints become the central challenge. In terms of computational time, the seven-component single-family test completed in approximately 1.2 hours, comparable to the six-component case. For the eight-component space, the total runtime would grow proportionally with the number of families and compositions. However, this time can be substantially reduced either by increasing the number of computing nodes, leveraging the near-linear scalability of our framework, or by adopting the batched task configuration, where each worker pulls multiple compositions per task to reduce Redis overhead and accelerate throughput. As demonstrated for the six-component system (Response to Question 6), this batched approach significantly reduced the total runtime from approximately one week to 26.7 hours. These results indicate that while the eight-component space presents considerable resource demands, our framework offers multiple tunable strategies to manage the trade-off between memory usage and computational speed.

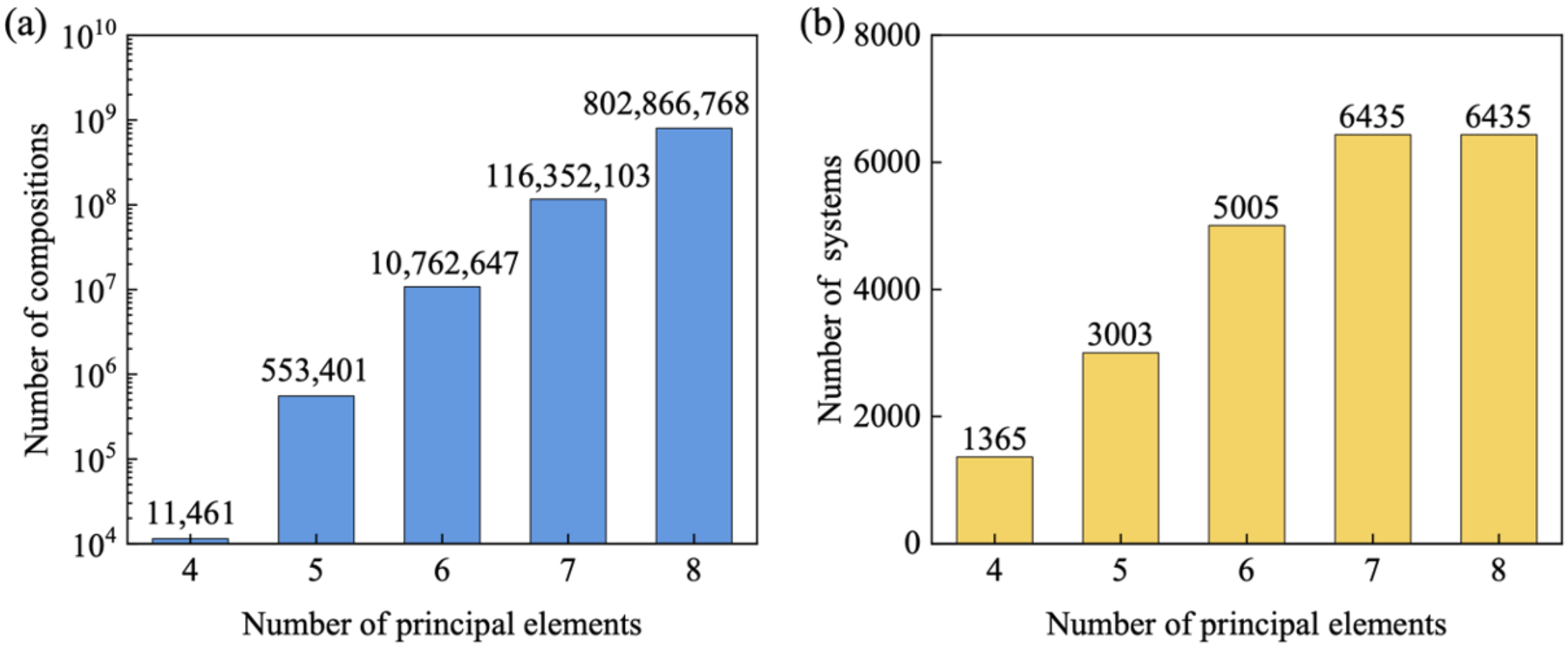

Fig. R3. Comparison of (a) the number of possible compositions per system and (b) the number of systems formed from a 15-element pool for four- to eight-component HEAs.

We have added a discussion of this point in the revised manuscript to clarify the scalability of the framework to higher-order systems.

Revised Section:

Revisions in the Manuscript

Page 17: line 547 to line 563 (Section 3.1 The large-scale database of HEAs)

We further discuss the scalability of our framework across HEA systems with varying numbers of principal components. The total number of compositions reaches approximately 1.6×10^7 for four-component systems, 1.7×10^9 for five-component systems, 5.4×10^{10} for six-component systems, 7.5×10^{11} for seven-component systems, and 5.2×10^{12} for eight-component systems, all drawn from a pool of 15 elements. Four- and five-component systems are computationally and storage-wise straightforward, as their composition spaces remain manageable. In contrast, seven- and eight-component systems bring substantial increases in both computational cost and storage footprint, with these resource demands roughly tens to hundreds of times higher than those of the six-component system. The computational time, however, can be flexibly managed by scaling the number of computing nodes or by adjusting the configurations of our framework such as the number of Redis queues and the batch size per worker. Storage, by contrast, is likely to become the primary bottleneck for higher-order HEA systems. Even with the substantial storage compression achieved by our columnar format, the

eight-component space still demands considerable storage capacity, which can be accommodated through scalable storage configurations or mitigated by adopting adaptive alloy search strategies that narrow the search space.

Question 12: In the Summary and outlook section, the authors may want to point out other areas of materials research for their approach. Also, the limits of their approach should be discussed. One of the fundamental challenges in solid state science is the prediction of crystal structures. In the present case of HEAs, this question is avoided by considering random solid solution in a given lattice (BCC in this work). This is a very serious limitation. What happens if a system develops short-range order or precipitates second phase particles? Futhermore, in a microstructure, segregation to grain boundaries could have a significant effect on the macroscopic properties. Magnetism adds another major complication when atoms like Mn, Fe, Cr, Ni, and Co are present. This does not diminish the merits of the present machine-learning approach, but materials science is more than scaling to trillions of random configurations.

Response: We thank the reviewer for this thoughtful comment. We agree that the original Summary and Outlook section did not adequately discuss the limitations of our framework. We have now supplemented it accordingly, and we address each point raised by the reviewer below.

The current design of our framework is optimized for workloads where computational tasks can be partitioned into independent and repetitive subtasks. This premise is central to the stateless parallel architecture that enables our scalability and efficiency. For tasks involving interdependent or non-uniform subtasks, the applicability of our framework relies on the careful design of a subtask partitioning strategy. We have added a discussion of this limitation in the Summary and Outlook section and noted that adapting the framework to a broader range of computational tasks will be a focus of our future work.

We fully agree with the reviewer's broader observation that materials science extends far beyond scaling to random configurations. We also acknowledge that crystal

structure prediction remains one of the most fundamental challenges in solid-state science. Our intention is not to reduce materials discovery to a combinatorial problem, but rather to provide an enabling infrastructure that, when combined with sound physics and materials knowledge, can accelerate the exploration of scientifically meaningful alloy spaces.

With respect to the prediction and design of HEAs, the present work focuses on BCC systems as a demonstration. The results show that by enabling the construction and exploration of much larger composition spaces, we can identify promising candidates that would otherwise remain undiscovered. This capability will be further explored in future work through strategies such as active learning. Beyond BCC systems, our framework can be applied to other complex HEA scenarios involving microstructural features such as short-range order, second-phase precipitation, and segregation, as well as magnetic effects. We emphasize that the framework itself is not the limiting factor in these extensions. The real challenge lies in formulating a sound materials science strategy for each specific problem. For systems with short-range order, dedicated descriptors capturing local chemical environment could be incorporated into the prediction models. For alloys with precipitates, phase-specific data can be collected to construct phase prediction models. For segregation-prone systems, dedicated datasets using parameters such as segregation energy can be established. For magnetic alloys, spin-polarized first-principles calculations would be required to capture the underlying physics. In all cases, new descriptor libraries can be specifically calculated or compiled, and their integration into our framework will not be constrained by the underlying computational infrastructure. Beyond the alloy design case demonstrated here, our framework can also support other areas of materials research, such as phase stability prediction, diffusion kinetics modeling, and high-temperature mechanical property screening, where large-scale computational campaigns are similarly constrained by data management and computational throughput. In future work, we will leverage our framework to accelerate these multiscale computational tasks and devise appropriate subtask partitioning strategies tailored to each specific problem.

We hope this addresses the reviewer's concerns and clarifies both the scope and limitations of our work.

Revised Section:

Revisions in the Manuscript

Page 22: line 662 to line 681 (Section 4 Summary and outlook)

The framework is designed for computational tasks that can be partitioned into independent and repetitive units. For tasks involving interdependent subtasks or heterogeneous workloads, effective application requires careful design of subtask partitioning strategies. The present demonstration focuses on BCC HEAs, but the framework is not restricted to this context. Extension to more complex systems is feasible with appropriately designed materials strategies. These systems include those exhibiting precipitation, segregation, or magnetism. For precipitation-strengthened alloys, phase-specific prediction models could be constructed using phase data. Descriptors for these models could be derived from empirical formulas, thermodynamic calculations, or first-principles computations. For segregation-prone systems, dedicated datasets could be built using segregation parameters as prediction targets. For magnetic systems, spin-polarized first-principles calculations would be required to capture the underlying physics. Future work will explore these extensions, with an emphasis on thermodynamics and multiscale modeling. It will also involve the development of tailored subtask partitioning strategies. The framework will support in-depth scientific exploration across expanded compositional spaces. Active learning will be integrated to guide the search and accelerate the discovery of novel materials.
